# Pharmacological and Toxicological Screening of Novel Benzimidazole-Morpholine Derivatives as Dual-Acting Inhibitors

**DOI:** 10.3390/molecules22081374

**Published:** 2017-08-19

**Authors:** Nafiz Öncü Can, Ulviye Acar Çevik, Begüm Nurpelin Sağlık, Yusuf Özkay, Özlem Atlı, Merve Baysal, Ümide Demir Özkay, Özgür Devrim Can

**Affiliations:** 1 Department of Analytical Chemistry, Faculty of Pharmacy, Anadolu University, Eskişehir 26470, Turkey; 2 Doping and Narcotic Compounds Analysis Laboratory, Faculty of Pharmacy, Anadolu University, Eskişehir 26470, Turkey; 3 Department of Pharmaceutical Chemistry, Faculty of Pharmacy, Anadolu University, Eskişehir 26470, Turkey; uacar@anadolu.edu.tr (U.A.Ç); bnsaglik@anadolu.edu.tr (B.N.S.); yozkay@anadolu.edu.tr (Y.Ö.); 4 Department of Pharmaceutical Toxicology, Faculty of Pharmacy, Anadolu University, Eskişehir 26470, Turkey; oatli@anadolu.edu.tr (Ö.A.); mbaysal@anadolu.edu.tr (M.B.); 5 Department of Pharmacology, Faculty of Pharmacy, Anadolu University, Eskişehir 26470, Turkey; udemir@anadolu.edu.tr (Ü.D.Ö.); ozgurdt@anadolu.edu.tr (Ö.D.C.)

**Keywords:** benzimidazoles, morpholines, AChE, MAO, COX

## Abstract

The aim of this study was to investigate acetylcholinesterase (AChE), monoamine oxidase A (MAO-A), monoamine oxidase B (MAO-B), cyclooxygenase-1 (COX-1) and cyclooxygenase-2 (COX-2) enzyme inhibitory, and antimicrobial activities of a new series of 2-(4-substituted phenyl)-1-[2-(morpholin-4-yl)ethyl]-1*H*-benzimidazole derivatives, for their possible use as multi-action therapeutic agents. Target compounds (*n* = 15) were synthesized under microwave irradiation conditions in two steps, and their structures were elucidated by FT-IR, ^1^H-NMR, ^13^C-NMR and high resolution mass spectroscopic analyses. Pharmacological screening studies revealed that two of the compounds (**2b** and **2j**) have inhibitory potential on both COX-1 and COX-2 enzymes. In addition, cytotoxic and genotoxic properties of the compounds **2b**, **2j** and **2m** were investigated via the well-known MTT and Ames tests, which revealed that the mentioned compounds are non-cytotoxic and non-genotoxic. As a concise conclusion, two novel compounds were characterized as potential candidates for treatment of frequently encountered inflammatory diseases.

## 1. Introduction

**Figure 1:**
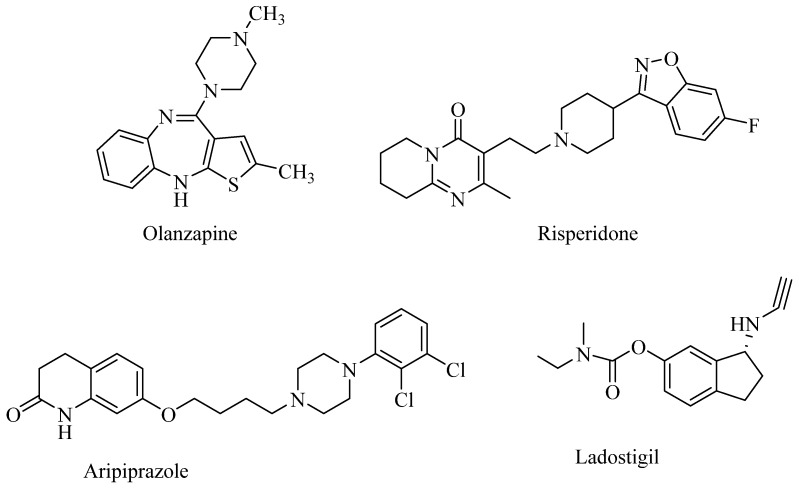
Chemical structures of some agents with dual inhibitory effect.

One of the promising advances in drug discovery is the simultaneous combination of two or more beneficial chemical moieties on the same compound, especially for treatment of a certain disease. Any pharmaceutical benefit that reduces the demand on polypharmacy, e.g., treatment while decreasing side effects, eliminating symptoms, or addition of an adjuvant therapeutic activity is accepted as an ideal starting-point that may lead to the development of novel compounds with multiple pharmacological effects [1]. There is a consensus that multifactorial disorders can originate from many sources, so searching for new hybrid compounds with multiple pharmacological profiles that can be expressed through more than one biochemical pathway would be useful for treating many kinds of diseases [2,3]. Despite the great challenge in the design and optimization of such compounds, this strategy possesses clear advantages over drug mixtures or multicomponent drugs owing to minimization of drug-drug interaction risks [4]. Some available drugs present the capability to modulate more than one bioreceptor, as exemplified by the atypical antipsychotics olanzapine, risperidone and aripiprazole, which act mainly as dopamine and serotonin receptor antagonists and have lower affinity to histamine, cholinergic muscarinic and α-adrenergic receptors [5]. Ladostigil is another example of dual monoamine oxidase B (MAO-B) and acetylcholine esterase (AChE) inhibitor [6] (Figure 1). Despite their great promise in the clinical application, any potential risk of side effects should not be neglected. The dual inhibitors are often characterized by high molecular weight that may reduce the chance of their drug abilities. Therefore, the safety profiles as well as pharmacokinetic properties need to be thoroughly considered in the design of dual inhibitors [7].

**Figure 2:**
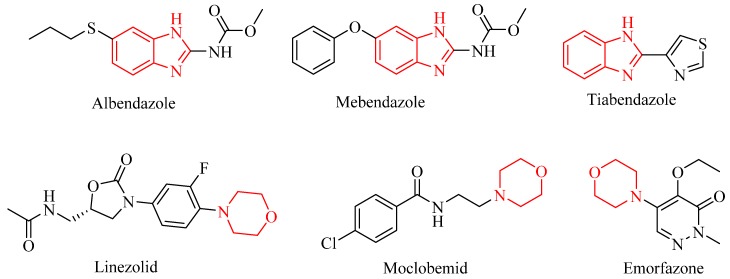
Chemical structures of some drugs containing benzimidazole or morpholine moieties.

Benzimidazole derivatives have a prominent position in medicinal chemistry, which are always used as one of the essential starting materials for discovery of new therapeutics. The origin of the special interest towards benzimidazole derivatives has been the 5,6-dimethyl-1-(α-d-ribofuranosyl)-benzimidazole structure, which is a basic part of vitamin B12 [8]. Furthermore, the benzimidazole ring is a structural bioisostere of some of the nucleobases existing in natural nucleotides, which can interact easily with the biopolymers in living systems [9]; this feature is often accepted as the responsible for its biological importance, either alone or as incorporated into different templates. It has been reported to show many pharmacological activities including antimicrobial, MAO or cyclooxygenase (COX) inhibitory, or anticholinesterase [10,11,12,13,14,15,16,17,18]. Besides, albendazole, mebendazole and tiabendazole are some examples of antimicrobial agents that carry the benzimidazole ring (Figure 2). Morpholine is another heterocyclic organic compound, which is also one of the principle building blocks in organic synthesis; several derivatives of morpholines have received attention in the past, due to their significant and numerous pharmacological activities such as antiinflammatory, antidepressant, MAO inhibitor, AChE inhibitor, neuroprotective, antituberculosis, antimalarial and antiparasitic [19,20,21]. Moreover, antibacterial agent linezolid, the MAO-A inhibitor moclobemid, and the antiinflamatory agent emorfazone are some examples of marketed drugs bearing morpholine moieties (Figure 2).

There are several examples in previously published papers in which the strategy described above was successfully applied by combining benzimidazole and morpholine pharmacophores on the same chemical structure; researchers have designed and synthesized many benzimidazole-morpholine compounds and investigate their pharmacological activities such as anticholinesterase [22,23], antimicrobial [24,25,26], antiinflammatory [27,28,29,30] and MAO inhibitory properties [31,32,33,34,35,36,37,38,39].

The antimicrobial and COX inhibitory potential of benzimidazole and morpholine-based compounds may be favorable in the design of dual COX inhibitory-antibacterial agents because inflammation and infection are conditions which are frequently encountered together. A decrease of fever besides elimination of an infectious microorganism or treatment of inflammation besides removal of pain are dual outcomes of such agents [40,41]. Besides, the inhibition potency of benzimidazole and morpholine derivatives against AChE and MAO enzymes may be beneficial in the design of new anti-Alzheimer’s disease agents. Inhibition of AChE increases neurotransmission in the cholinergic synapses and temporally decreases the cognitive deficit. AChE also contributes in other functions related to neuronal development, differentiation, adhesion and β-amyloid protein processing. Additionally, MAO-B inhibition retards further deterioration of cognitive functions. Thus, discovery of a dual inhibitor of these enzymes are expected to have potential for the treatment of Alzheimer’s disease [42].

It is clear that, the benzimidazole-morpholine combination has the potential to serve as a pharmaceutical source for therapy. With the aim of producing safer and more active hybrid compounds, the synthesis and pharmacological evaluation of some novel molecules that include bioactive benzimidazole and morpholine moieties have been presented in this study.

## 2. Results and Discussion

### 2.1. Chemistry

**Scheme 1:**
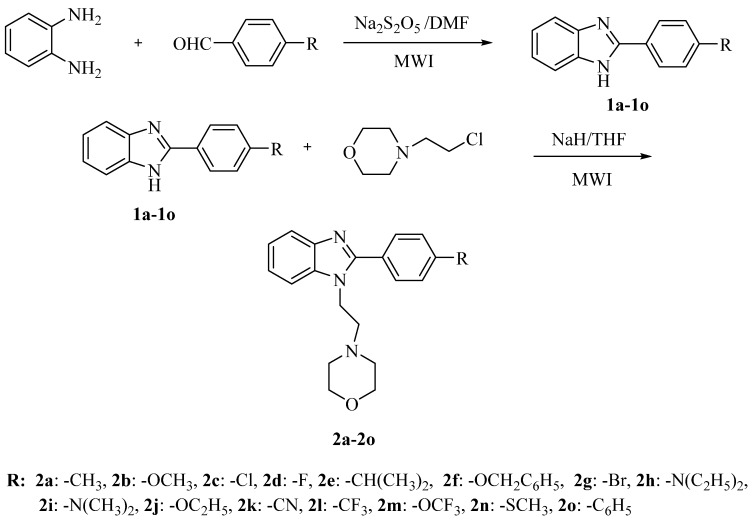
Synthesis route to the target compounds **2a–2o**.

Synthesis of the target compounds **2a–2o** is outlined in Scheme 1. Initially, 1,2-phenylenediamine was reacted with various sodium bisulfite adducts of aldehydes under microwave irradiation conditions to obtain the previously reported 2-(4-substitutedphenyl)-1*H*-benzimidazoles **1a–1o** [43,44,45,46,47,48,49,50]. In the second step, compounds **1a–1o** were treated with 2-(morpholin-4-yl)ethyl chloride in the presence of NaH, and subjected to microwave irradiation again. In the synthesis, various substituents that may have an impact on biological activity were selected to impart different electronic and hydrophobic natures to the target compounds.

Structure elucidations of the final compounds **2a–2o** were realized by FT-IR, ^1^H-NMR, ^13^C-NMR and HRMS methods. The aromatic C-H and aliphatic C-H stretching bands were observed at 3063–3011 cm^−1^ and 2986–2945 cm^−1^, respectively in the IR spectra; C=N stretching bands were recorded at 1618–1599 cm^−1^. The out of plane bands, which were assigned to 1,4-disubstituted benzene, were at 868–818 cm^−1^. The protons at the positions 3 and 4 of morpholine structure were recorded at about 2.20 ppm as multiplet, in the ^1^H-NMR spectra. In addition, the protons of ethylene near the morpholine and benzimidazole were observed as a triplet at about 2.50 ppm and 4.40 ppm, respectively. The protons at the positions 1 and 2 of morpholine were assigned as multiplet at about 3.40 ppm. Aromatic protons are appeared between 7.10 ppm and 7.90 ppm, as expected.

In the ^13^C-NMR spectra, the signals of the ethylene spacer between the benzimidazole and morpholine units appeared between 42.03 ppm and 42.36 ppm, and the other carbons of that chain was recorded between 57.39 ppm and 57.55 ppm. Morpholine carbons had two different peaks at 53.78–53.83 ppm and 66.31–66.50 ppm. The benzimidazole carbon at the position 2 gave a peak between 157.27 ppm and 154.69 ppm, depending on substituent group. The other benzimidazole-carbon peaks were recorded between 111.01 ppm and 143.30 ppm. The chemical shifts of the substituent groups and coupling constant of the fluorinated derivatives were consistent with the theoretical values. HRMS findings were in accordance with the theoretical molecular formula of the compounds **2a–2o**.

### 2.2. Biological Activity Screening

The antimicrobial abilities of the compounds against different human pathogens were evaluated. *Escherichia coli* ATCC 35218, *Escherichia coli* ATCC 25922, *Pseudomona aeuroginosa* ATCC 27853, and *Klebsiella pneumoniae* ATCC 700603 were used as Gram-negative bacteria. As Gram-positive bacteria *Staphylococcus aureus* ATCC 25923, *Enterococcus faecalis* ATCC29212, and *Listeria monocytogenes* ATCC 7644 were tested. *Candida albicans* ATCC 90028, *Candida glabrata* ATCC 90030, *Candida krusei* ATCC 6258 and *Candida parapsilosis* ATCC 22019 yeasts were also used in antimicrobial activity tests. Chloramphenicol and ketoconazole were used as the reference drugs. Antimicrobial activity results are given in Table 1.

Table 1 reveals that the microbial strains tested herein are very resistant to compounds **2a–2o**; and only *Pseudomona aeuroginosa* ATCC 27853 was sensitive to some of the compounds. The compound **2e**, which bears an isopropyl substituent, showed equal activity (MIC_50_ = 250 µg/mL) to the reference drug chloramphenicol against *Pseudomona aeuroginosa* ATCC 27853. The MIC_50_ values (MIC_50_ = 125 µg/mL) of compounds **2c** and **2m**, which bear trifluoromethoxy and chloro substituents, was 2-fold lower than that of chloramphenicol. The most active compound in the series was 2-(4-methoxyphenyl)-1-[2-(morpholin-4-yl)ethyl]-1*H*-benzimidazole (**2b**), which was a methoxy-substituted derivative and showed a 16-fold greater activity than the reference drug with a MIC_50_ value of 0.0156 µg/mL. These findings show that synthesized compounds do not have broad antibacterial spectrum and suggest that incorporation of methoxy substituent enhances the antibacterial activity against *Pseudomona aeuroginosa* ATCC 27853.

The inhibitory potencies of the synthesized compounds against AChE, MAO-A, MAO-B, COX-1 and COX-2 enzymes were studied according to previously verified protocols [51,52,53,54]. In all of the assays, the compounds were initially tested at two concentrations (10^−3^ M and 10^−4^ M); in case of an inhibition more than 50% for any of the compounds, further studies were performed to determine the IC_50_, covering a wider range of concentrations (10^−5^ M and 10^−9^ M).

Inhibitory activity results of the synthesized compounds on AChE, MAO-A and MAO-B enzymes are summarized in Supplementary Table S1; donepezil, moclobemide and selegiline were used as the reference drugs for AChE, MAO-A and MAO-B inhibition tests, respectively. Relatively low inhibition against the tested enzymes was observed, when compared to the reference drugs; besides, it can be underlined that the compounds possessed better inhibitory profile against MAO-B, in comparison to AChE and MAO-A. Since none of the compounds showed more than 50% inhibition, further assays were not carried out to determine IC_50_ values. This indicates that the synthesized compounds have no potential as drug candidates for AChE- and MAO-related disorders.

The inhibitory potencies of compounds **2a–2o** on COX-1 and COX-2 enzymes are given in Supplementary Table S2; ibuprofen and nimesulide were used as non-selective COX and selective COX-2 inhibitors in these tests, respectively. The compounds **2a**, **2b**, **2f**, **2h**, **2j** and **2m** displayed more than 50% inhibition at the initial concentrations (10^−3^ M and 10^−4^ M); consequently, they were assayed at lower concentrations between 10^−5^ M and 10^−9^ M, and IC_50_ values were calculated (Table 2). It has been determined that compounds **2b** and **2j** have promising inhibitory activity against both COX-1 and COX-2 enzymes. IC_50_ values of the compound **2b** was 8.096 µM and 8.369 µM versus COX-1 and COX-2 enzymes, respectively; their IC_50_ values were also comparable with those of the reference drugs ibuprofen and nimesulide. Compound **2j** possessed 14.18 and 13.09 µM IC_50_ values on COX-1 and COX-2, respectively.

In terms of structure activity relationships, it is noted that the most active compounds **2b** and **2j** carry methoxy- and ethoxy- substituents, respectively, at the 4th position of the phenyl on the benzimidazole ring; in addition, when the nature of the substituents was examined, it can be easily recognized that only compounds **2b**, **2f**, **2j** and **2m** included alkyloxy groups. Besides **2b** and **2j**, the compounds **2f** and **2m** also showed potential activity in initial assays, and were subjected to further tests. Thus, it may be suggested that the substituents including oxygen atoms may cause more inhibition than the other substituents; this phenomenon may be explained by the hydrogen accepting ability of alkyloxy groups.

### 2.3. Toxicological Studies

It is a well-known fact that a drug candidate should not only possess a beneficial pharmacological activity, but also has to display a low toxicological profile; from this point of view, the MTT cell viability and Ames genotoxicity tests were applied. The cytotoxicity results of the most active compounds (**2b**, **2j** and **2m**) against COX enzymes are presented in Table 3. IC_50_ values of the compounds **2b**, **2j** and **2m** against NIH/3T3 was found as 316 μM, 316 μM and 100 μM, respectively. IC_50_ of the mentioned compounds against NIH/3T3 is about 5–40 folds higher than their IC_50_ against COX enzymes. Thus, it can be stated that the compounds are non-toxic at their effective concentrations against COX-1 and COX-2.

The Ames assay is a widely used method that use bacteria to test if a compound causes mutations on the DNA of the test microorganism. More formally, it is a biological assay to assess the mutagenic potential of a chemical compound. A positive result indicates that the tested chemical is mutagenic and hence may act as a carcinogen, since cancer is frequently related to mutation. It is a quick and convenient test to evaluate the carcinogenic potential of a compound [55]. Accordingly, this assay was performed to investigate the genotoxicity of compounds **2b**, **2j** and **2m**. In Ames MPF assay, more than 25 positive wells were observed with our positive controls, and negative control wells also showed less than eight positive wells in the presence and absence of S9 with TA98 and TA100; these results are in compliance with previous studies and the requirements for the validation of the Ames MPF [55]. The results are presented in Table 3.

**Figure 3:**
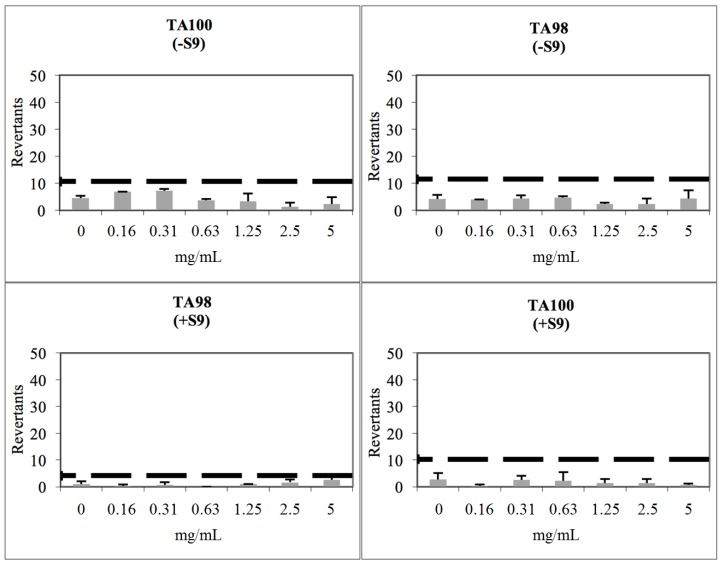
Dose-Response curve of compound **2b** against TA98 and TA100 in the presence and absence of S9 according to Ames MPF test (the thick black line corresponds to mutagenicity threshold).

Compound **2b** showed a baseline of 5.77 with TA98 in the absence of S9 and 2.10 in the presence of S9. None of the tested concentrations reached the mentioned values above the baseline, and also did not show any significance. Therefore, compound **2b** was classified as non-mutagenic against TA98 in the presence/absence of metabolic activation (S9, Figure 3). Compound **2b** had a baseline of 5.35 with TA100 in the absence of S9 and 5.14 in the presence of S9. Furthermore, fold inductions over baseline were less than 1.5 in each concentration level of the compounds, and the results did not show a dose-response tendency. Therefore, compound **2b** was concluded as non-genotoxic against TA100 with/without metabolic activation (Figure 3).

**Figure 4:**
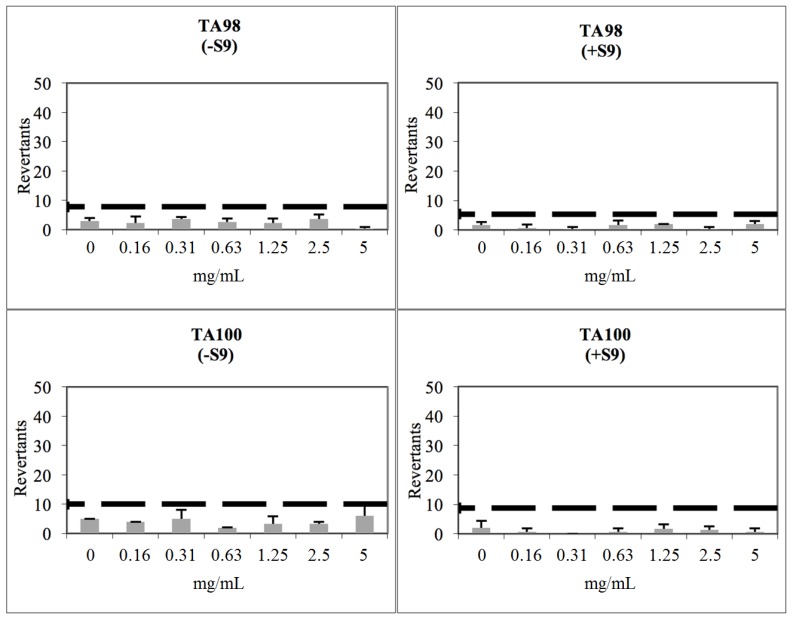
Dose-Response curve of compound **2j** against TA98 and TA100 in the presence and absence of S9 according to Ames MPF test (the thick black line corresponds to mutagenicity threshold).

Compound **2j** showed a baseline of 2.70 and 3.89 against TA98 with/without S9, respectively. Fold inductions over baseline did not reach values more than 1.5 or 2 and, statistically dissimilar results did not reveal a dose-response tendency. According to these findings, compound **2j** accepted as non-mutagenic against TA98 (Figure 4). Compound **2j** was found to show a baseline of 4.37 and 5.00 with/without S9 against TA100. Mentioned fold-increases over the baseline according to the criteria were not observed with compound **2j**, and significant results did not reach these values, showing a non-monotonic dose-response tendency. Compound **2j** was also found to be non-mutagenic against TA100 in the presence/absence of metabolic activation (Figure 4).

**Figure 5:**
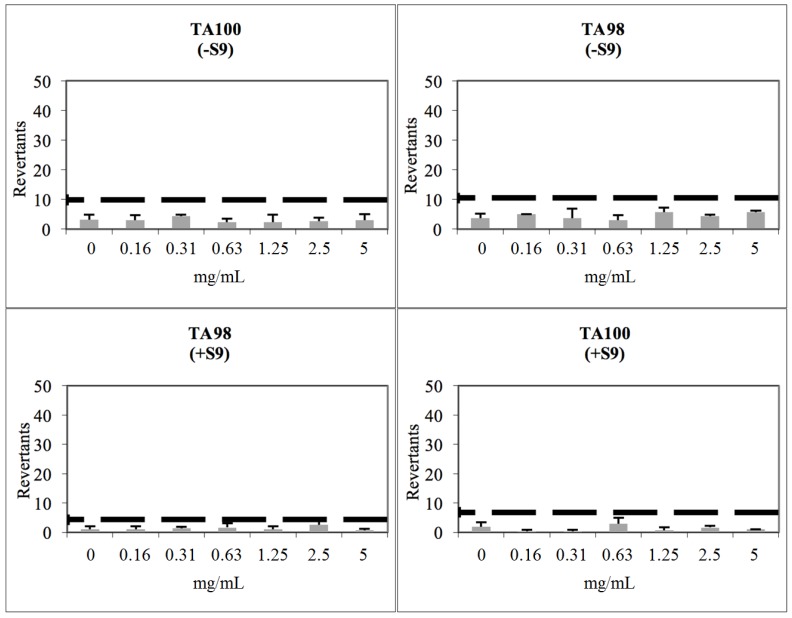
Dose-Response curve of compound **2m** against TA98 and TA100 in the presence and absence of S9 according to Ames MPF test (the thick black line corresponds to mutagenicity threshold).

Compound **2m** was found to show a baseline of 5.26 and 2.17 in the absence and presence of S9 against TA98, respectively. None of the concentrations reached 1.5 or 2-fold increases over the baseline, according to the criteria. Significantly different results did not show a dose-response tendency and they were also lower than the mentioned fold-increases. Compound **2m** showed a baseline of 3.43 and 4.94 with/without S9, respectively against TA100. Fold inductions over baseline were less than 1.5 at each concentration level of the compounds, and there was not any significant difference. Compound **2m** was accepted as non-mutagenic against TA98 and TA100 with and without metabolic activation (Figure 5).

According to the Ames MPF results, compounds were classified as non-mutagenic. As well as cytotoxicity results, genotoxicity findings also increased the value of compounds **2b** and **2j** as possible COX inhibitors.

### 2.4. Prediction of ADME and Drug Likeness

Sufficient pharmacological activity and low toxicological profile are not the only prerequisites for a compound to become a drug candidate; in addition, acceptable pharmacokinetic profile is also required. Due to importance of such characteristics, in the current study, ADME properties of the synthesized compounds **2a–2o** were also investigated via in-silico routes, and related calculations were realized by using online Molinspiration software [56]. Drug likeness score (DLS) was calculated for all of the compounds **2a–2o** and reference drugs using Molsoft’s software [57]. The theoretical calculations of ADME parameters such as molecular weight (MW), logP, topological polar surface are (tPSA), number of hydrogen donors (nON) and acceptors (nOHNH), volume, and DLS are presented in Table 4, along with the violations of Lipinski’s rule. Considering Lipinski’s rule of five, all synthesized compounds have nON smaller than 5, nOHNH smaller than 10 and polar surface area lesser than 140 Å. Besides, MWs of all of the compounds are in accordance with the value smaller than 500 g/mol. Synthesized compounds have logP values of less than 5 except for compounds **2e**, **2l** and **2o**. According to theoretical data, the majority of the compounds were found to obey Lipinski’s rules; only the compounds **2e**, **2l** and **2o** showed minor deviation in one parameter. Furthermore, high DLSs of 1.12 and 0.88 were calculated for the most active compounds **2b** and **2m**. Thus, it can be stated that synthesized compounds have good pharmacokinetics profile, which improves their biological acceptability.

## 3. Materials and Methods

### 3.1. General Information

All of the chemicals used in the study were purchased either from Sigma-Aldrich Corp. (St. Louis, MO, USA) or Merck KGaA (Darmstadt, Germany), and used without further chemical or biological purification. Microwave syntheses were realized by using a Monowave 300 high-performance microwave reactor (Anton-Paar, Graz, Austria). Melting points of the synthesized compounds were determined by using a MP90 series automatic melting point determination system (Mettler-Toledo, Columbus, OH, USA) and were presented as uncorrected. ^1^H- and ^13^C-NMR spectra were recorded in DMSO-*d*_6_ by a Bruker digital FT-NMR spectrometer (Bruker Bioscience, Billerica, MA, USA) at 500 MHz and 75 MHz, respectively (splitting patterns in the NMR spectra were designated as follows: s: singlet; d: doublet; t: triplet; m: multiplet; coupling constants (*J*) were reported in Hertz). The IR spectra of the compounds were recorded using an IRAffinity-1S Fourier transform IR (FTIR) spectrometer and high resolution mass spectrometric studies were performed using a liquid chromatography mass spectrometry-ion trap-time of flight (LCMS-IT-TOF) instrument, both from Shimadzu (Kyoto, Japan). Chemical purities of the compounds were checked by classical TLC applications performed on silica gel 60 F254 (Merck KGaA); LCMS-IT-TOF chromatograms were also used for the same purpose. The pipetting in the COX-1, COX-2 and AChE assays were performed by using BioTek Precision XS robotic system (BioTek Instruments, Inc., Winooski, VT, USA). Fluorescence intensities were measured by BioTek-Synergy H1 multimode microplate reader (BioTek Instruments, Inc). A model UV-1800 double beam UV-visible spectrophotometer (Shimadzu) was used for spectrophotometric determinations. Statistical calculations were performed via Prism 5 software from GraphPad (La Jolla, IL, USA). Water (ultra-pure, with total organic carbon less than 5 ppb and resistivity of at least 18 MOhm·cm^−1^) was produced in our laboratory using Milli-Q Synthesis A10 system from Millipore SAS (Molsheim, France) and steam-sterilized before use.

### 3.2. Chemistry

#### 3.2.1. Microwave-Assisted Synthesis of 2-Substituted-1*H*-benzimidazole Derivatives **1a–1o**

An appropriate aldehyde derivative (0.03 mol), sodium bisulfite (5.7 g, 0.03 mol) and DMF (10 mL) were added into a 30-mL glass vial of microwave synthesis reactor. The vial was located into the reactor and heated at 240 °C under 10 bars of internal pressure for 5 min; consequently, it was cooled down to room temperature (RT), 1,2-phenylenediamine (3.24 g, 0.03 mol) was added and the mixture was subjected to above-mentioned conditions again. After cooling to RT, the mixture was poured into iced-water, the precipitated product was washed using water, dried at RT and recrystallized from ethanol.

#### 3.2.2. Microwave-Assisted Synthesis of 2-Substituted-1-[2-(morpholin-4-yl)ethyl]-1*H*-benzimidazole Derivatives **2a–2o**

The corresponding 2-substituted-1*H*-benzimidazole derivative **1a–1o** (0.0025 mol), NaH (0.072 g, 0.003 mol) and THF (10 mL) were added into a 30-mL glass vial of microwave synthesis reactor. After addition of 2-(morpholin-4-yl)ethyl chloride (1 mL), the vial was located into the reactor and heated at 170 °C under 10 bars of internal pressure for 30 min. The reaction vial was removed and cooled to RT, and the mixture was poured into iced-water. The precipitated product was washed using water, dried at RT and recrystallized from ethanol.

*2-(4-Methylphenyl)-1-[2-(morpholin-4-yl)ethyl]-1H-benzimidazole* (**2a**). Yield: 84%. m.p. 117.7–119.1 °C. FTIR (ATR, cm^−1^): 3017 (aromatic C-H), 2986 (aliphatic C-H), 1610 (C=N), 828 (*para*-substituted benzene). ^1^H-NMR (DMSO-*d*_6_, ppm) δ: 2.19–2.21 (4H, m, morpholine -CH_2_-), 2.41 (3H, s, -CH_3_), 2.51 (2H, t, *J* = 6.40 Hz, -CH_2_-), 3.37–3.39 (4H, m, morpholine -CH_2_-), 4.39 (2H, t, *J* = 6.40 Hz, -CH_2_-), 7.22–7.27 (2H, m, benzimidazole H_5_-H_6_), 7.29 (2H, d, *J* = 8.00 Hz, phenyl H_3_,H′_5_,), 7.69–7.71 (2H, m, benzimidazole H_4_,H_7_), 7.81 (2H, d, *J* = 8.00 Hz, phenyl H_2_,H′_6_). ^13^C-NMR (DMSO-*d*_6_, ppm): δ: 21.40, 42.13, 53.78, 57.43, 66.42, 111.33, 119.51, 122.31, 122.69, 128.30, 129.66, 136.10, 139.69, 143.13, 154.00. HRMS (*m*/*z*): [M + H]^+^ calcd. for C_20_H_23_N_3_O: 322.1914 found: 322.1901.

*2-(4-Methoxyphenyl)-1-[2-(morpholin-4-yl)ethyl]-1H-benzimidazole* (**2b**). Yield: 81%. m.p. 132.7–133.9 °C. FTIR (ATR, cm^−1^): 3019 (aromatic C-H), 2966 (aliphatic C-H), 1611 (C=N), 847 (*para*-substituted benzene). ^1^H-NMR (DMSO-*d*_6_, ppm) δ: 2.20–2.22 (4H, m, morpholine -CH_2_-), 2.50 (2H, t, *J* = 6.50 Hz, -CH_2_-), 3.38–3.40 (4H, m, morpholine -CH_2_-), 3.85 ( 3H, s, -OCH_3_), 4.38 (2H, t, *J* = 6.50 Hz, -CH_2_-), 7.11 (2H, d, *J* = 8.00 Hz, phenyl H_3_,H′_5_,), 7.21–7.26 (2H, m, benzimidazole H_5_-H_6_), 7.61–7.65 (2H, m, benzimidazole H_4_,H_7_), 7.76 (2H, d, *J* = 8.00 Hz, phenyl H_2_,H′_6_). ^13^C-NMR (DMSO-*d*_6_, ppm): δ: 42.15, 53.79, 55.78, 57.39, 66.44, 111.25, 114.53, 119.38, 122.27, 122.57, 123.32, 131.21, 136.09, 143.12, 153.88, 160.68. HRMS (*m*/*z*): [M + H]^+^ calcd. for C_20_H_23_N_3_O_2_: 338.1863 found: 338.1844.

*2-(4-Chlorophenyl)-1-[2-(morpholin-4-yl)ethyl]-1H-benzimidazole* (**2c**). Yield: 69%. m.p. 133.3–134.0 °C. FTIR (ATR, cm^−1^): 3055 (aromatic C-H), 2953 (aliphatic C-H), 1602 (C=N), 837 (*para*-substituted benzene). ^1^H-NMR (DMSO-*d*_6_, ppm) δ: 2.18–2.19 (4H, m, morpholine -CH_2_-), 2.58 (2H, t, *J* = 6.50 Hz, -CH_2_-), 3.36–3.37 (4H, m, morpholine -CH_2_-), 4.40 (2H, t, *J* = 6.50 Hz, -CH_2_-), 7.23–7.28 (2H, m, benzimidazole H_5_-H_6_), 7.62–7.69 (4H, m, benzimidazole H_4_,H_7_, phenyl H_2_,H′_6_), 7.86–7.88 (2H, phenyl H_3_,H′_5_). ^13^C-NMR (DMSO-*d*_6_, ppm) δ: 42.24, 53.82, 57.47, 66.39, 111.49, 119.70, 122.54, 123.02, 129.20, 130.08, 131.59, 134.92, 136.13, 143.08, 152.82. HRMS (*m*/*z*): [M + H]^+^ calcd. for C_19_H_20_N_3_OCl: 342.1368 found: 342.1348.

*2-(4-Fluorophenyl)-1-[2-(morpholin-4-yl)ethyl]-1H-benzimidazole* (**2d**). Yield: 73%. m.p. 91.1–92.0 °C. FTIR (ATR, cm^−1^): 3055 (aromatic C-H), 2972 (aliphatic C-H), 1605 (C=N), 845 (*para*-substituted benzene). ^1^H-NMR (DMSO-*d*_6_, ppm) δ: 2.15–2.17 (4H, m, morpholine -CH_2_-), 2.57 (2H, t, *J* = 6.50 Hz, -CH_2_-), 3.35–3.36 (4H, m, morpholine -CH_2_-), 4.38 (2H, t, *J* = 6.50 Hz, -CH_2_-), 7.23–7.30 (2H, m, benzimidazole H_5_-H_6_), 7.38–7.42 (2H, m, benzimidazole H_4_,H_7_), 7.63–7.67 (2H, m, phenyl H_2_,H′_6_), 7.86–7.88 (2H, phenyl H_3_,H′_5_). ^13^C-NMR (DMSO-*d*_6_, ppm) δ: 42.13, 53.79, 57.43, 66.39, 111.42, 116.15 (d, ^2^*J_CF_* = 21.6 Hz), 119.61, 122.45, 122.89, 127.70 (d, ^4^*J_CF_* = 3.1 Hz), 132.16 (d, ^3^*J_CF_* = 8.5 Hz), 136.03, 143.03, 153.07, 163.26 (d, ^1^*J_CF_* = 245.1 Hz). HRMS (*m*/*z*): [M + H]^+^ calcd. for C_19_H_20_N_3_OF: 326.1663; found: 326.1645.

*2-(4-Isopropylphenyl)-1-[2-(morpholin-4-yl)ethyl]-1H-benzimidazole* (**2e**). Yield: 76%. m.p. 123.1–124.0 °C. FTIR (ATR, cm^−1^): 3050 (aromatic C-H), 2967 (aliphatic C-H), 1611 (C=N), 843 (*para*-substituted benzene). ^1^H-NMR (DMSO-*d*_6_, ppm) δ: 1.26 (6H, d, *J* = 7.00 Hz, -CH_3_), 2.16–2.18 (4H, m, morpholine -CH_2_-), 2.51 (2H, t, *J* = 6.50 Hz, -CH_2_-), 2.58 (1H, s, -CH-), 3.33–3.35 (4H, m, morpholine -CH_2_-), 4.41 (2H, t, *J* = 6.50 Hz, -CH_2_-), 7.41–7.44 (2H, benzimidazole H_5_-H_6_), 7.49 (2H, m, d, *J* = 8.00 Hz, phenyl H_3_,H′_5_), 7.65–7.73 (2H, m, benzimidazole H_4_,H_7_), 7.75 (2H, d, *J* = 8.00 Hz, phenyl H_2_,H′_6_). ^13^C-NMR (DMSO-*d*_6_, ppm) δ: 24.22, 33.80, 42.03, 53.78, 57.52, 66.40, 111.31, 119.52, 122.31, 122.69, 127.00, 128.73, 129.80, 136.06, 143.15, 150.44, 154.03.HRMS (*m*/*z*): [M + H]^+^ calcd. for C_22_H_27_N_3_O: 350.2227; found: 350.2199.

*2-(4-Benzyloxyphenyl)-1-[2-(morpholin-4-yl)ethyl]-1H-benzimidazole* (**2f**). Yield: 63%. m.p. 160.9–161.9 °C. FTIR (ATR, cm^−1^): 3051 (aromatic C-H), 2961 (aliphatic -C-H), 1611 (C=N), 831 (*para*-substituted benzene), 744 (monosubstituted benzene), 702 (monosubstituted benzene).^1^H-NMR (DMSO-*d*_6_, ppm) δ: 2.20 (4H, s, morpholine -CH_2_-), 2.59 (2H, t, *J* = 6.50 Hz, -CH_2_-), 3.38 (4H, s, morpholine -CH_2_-), 4.38 (2H, t, *J* = 6.50 Hz, -CH_2_-), 5.13 (2H, s, -OCH_2_-), 7.18–7.26 (5H, m, benzyloxy -CH-), 7.35–7.40 (2H, m, benzimidazole H_5_-H_6_), 7.48 (2H, d, *J* = 8.50 Hz, phenyl H_2_,H′_6_), 7.61–7.65 (2H, m, benzimidazole H_4_,H_7_), 7.76 (2H, d, *J* = 8.50 Hz, phenyl H_3_,H′_5_). ^13^C-NMR (DMSO-*d*_6_, ppm) δ: 42.13, 53.78, 57.39, 66.43, 66.80, 111.26, 115.40, 119.40, 122.27, 122.57, 123.56, 128.22, 128.39, 128.94, 131.22, 136.08, 137.26, 143.13, 153.85, 159.74. HRMS (*m*/*z*): [M + H]^+^ calcd. for C_26_H_27_N_3_O_2_: 414.2176; found: 414.2156.

*2-(4-Bromophenyl)-1-[2-(morpholin-4-yl)ethyl]-1H-benzimidazole* (**2g**). Yield: 80%. m.p. 166.4–167.3 °C. FTIR (ATR, cm^−1^): 3053 (aromatic C-H), 2953 (aliphatic C-H), 1608 (C=N), 837 (*para*-substituted benzene). ^1^H-NMR (DMSO-*d*_6_, ppm) δ: 2.18 (4H, s, morpholine -CH_2_-), 2.58 (2H, t, *J* = 6.50 Hz, -CH_2_-), 3.39 (4H, s, morpholine -CH_2_-), 4.40 (2H, t, *J* = 6.50 Hz, -CH_2_-), 7.25–7.30 (2H, m, benzimidazole H_5_-H_6_), 7.66–7.68 (2H, m, phenyl H_2_,H′_6_), 7.79–7.81 (4H, m, benzimidazole H_4_,H_7_, phenyl H_3_,H′_5_). ^13^C-NMR (DMSO-*d*_6_, ppm) δ: 42.25, 53.82, 57.48, 66.38, 111.52, 119.69, 122.55, 123.04, 123.66, 130.44, 131.82, 132.13, 136.14, 143.06, 152.89. HRMS (*m*/*z*): [M + H]^+^ calcd. for C_19_H_20_N_3_OBr: 386.0862; found: 386.083.

*2-(4-Diethylaminophenyl)-1-[2-(morpholin-4-yl)ethyl]-1H-benzimidazole* (**2h**). Yield: 79%. m.p. 140.2–141.4 °C. FTIR (ATR, cm^−1^): 3047 (aromatic C-H), 2970 (aliphatic C-H), 1609 (C=N), 818 (*para*-substituted benzene). ^1^H-NMR (DMSO-*d*_6_, ppm) δ: 1.13 (6H, t, *J* = 7.00 Hz, -CH_3_), 2.27–2.29 (4H, m, morpholine -CH_2_-), 2.63 (2H, s, -CH_2_-), 3.41–3.44 (8H, m, morpholine -CH_2_-, -CH_2_-), 4.38 (2H, t, *J* = 6.50 Hz, -CH_2_-), 6.78–6.80 (2H, m, phenyl H_2_,H′_6_), 7.20 (2H, s, benzimidazole H_5_-H_6_), 7.59–7.63 (4H, m, benzimidazole H_4_,H_7_, phenyl H_3_,H′_5_). ^13^C-NMR (DMSO-*d*_6_, ppm) δ: 12.85, 42.23, 44.15, 53.83, 57.44, 66.49, 110.94, 111.39, 116.81, 118.99, 122.02, 122.06, 130.82, 136.25, 143.30, 148.56, 154.69. HRMS (*m*/*z*): [M + H]^+^ calcd. for C_23_H_30_N_4_O: 379.2492; found: 379.2465.

*2-(4-Dimethylaminophenyl)-1-[2-(morpholin-4-yl) ethyl]-1H-benzimidazole* (**2i**). Yield: 72%. m.p. 162.0–163.3 °C. FTIR (ATR, cm^−1^): 3053 (aromatic C-H), 2955 (aliphatic C-H), 1607 (C=N), 824 (*para*-substituted benzene). ^1^H-NMR (DMSO-*d*_6_, ppm) δ: 2.28 (4H, s, morpholine -CH_2_-), 2.50 (2H, s, -CH_2_-), 2.99 (6H, s, -CH_3_), 3.42–3.44 (4H, m, morpholine -CH_2_-), 4.38 (2H, t, *J* = 6.50 Hz, -CH_2_-), 6.84 (2H, d, *J* = 8.85 Hz, phenyl H_2_,H′_6_), 7.19–7.22 (2H, m, benzimidazole H_5_-H_6_), 7.57–7.61 (2H, m, benzimidazole H_4_,H_7_), 7.66 ( 2H, d, *J* = 8.85 Hz, phenyl H_3_,H′_5_). ^13^C-NMR (DMSO-*d*_6_, ppm) δ: 40.28, 42.28, 53.83, 57.39, 66.50, 111.01, 112.11, 117.85, 119.06, 122.06, 122.15, 130.53, 136.25, 143.27, 151.36 154.59. HRMS (*m*/*z*): [M + H]^+^ calcd. for C_21_H_26_N_4_O: 351.2179; found: 351.2172.

*2-(4-Ethoxyphenyl)-1-[2-(morpholin-4-yl) ethyl]-1H-benzimidazole* (**2j**). Yield: 76%. m.p. 83.4–85.5 °C. FTIR (ATR, cm^−1^): 3063 (aromatic C-H), 2972 (aliphatic C-H), 1612 (C=N), 854 (*para*-substituted benzene). ^1^H-NMR (DMSO-*d*_6_, ppm) δ: 1.37 (3H, t, *J* = 6.95 Hz, -CH_3_), 2.21 (4H, s, morpholine -CH_2_-), 2.50 (2H, s, -CH_2_-), 3.39 (4H, s, morpholine -CH_2_-), 4.12 (2H, q, *J* = 6.95 Hz, -CH_2_-), 4.38 (2H, t, *J* = 6.50 Hz, -CH_2_-), 7.08 (2H, d, *J* = 8.75 Hz, phenyl H_2_,H′_6_), 7.21–7.26 (2H, m, benzimidazole H_5_-H_6_), 7.61–7.65 (2H, m, benzimidazole H_4_,H_7_), 7.75 (2H, d, *J* = 8.75 Hz, phenyl H_3_,H′_5_). ^13^C-NMR (DMSO-*d*_6_, ppm) δ: 15.05, 42.13, 53.79, 57.39, 63.74 66.44, 111.24, 114.94, 119.37, 122.25, 122.55, 123.17, 131.21, 136.09, 143.13, 153.92, 159.95. HRMS (*m*/*z*): [M + H]^+^ calcd. for C_21_H_25_N_3_O_2_: 352.2020; found: 352.1985.

*2-(4-Cyanophenyl)-1-[2-(morpholin-4-yl)ethyl]-1H-benzimidazole* (**2k**). Yield: 83%. m.p. 134.9–136.2 °C. FTIR (ATR, cm^−1^): 3059 (aromatic C-H), 2952 (aliphatic C-H), 2226 (C≡N), 1612 (C=N), 844 (*para*-substituted benzene). ^1^H-NMR (DMSO-*d*_6_, ppm) δ: 2.20 (4H, s, morpholine -CH_2_-), 2.58 (2H, t, *J* = 6.50 Hz, -CH_2_-), 3.38 (4H, s, morpholine -CH_2_-), 4.43 (2H, t, *J* = 6.50 Hz, -CH_2_-), 7.28–7.33 (2H, m, benzimidazole H_5_-H_6_), 7.70–7.72 (2H, m, phenyl H_2_,H′_6_), 8.08–8.06 (4H, m, benzimidazole H_4_,H_7_, phenyl H_3_,H′_5_). ^13^C-NMR (DMSO-*d*_6_, ppm) δ: 42.36, 53.82, 57.51, 66.34, 111.69, 112.48, 118.98, 119.96, 122.80, 123.45, 130.64, 133.05, 135.77, 136.23, 143.10, 152.27. HRMS (*m*/*z*): [M + H]^+^ calcd. for C_20_H_20_N_4_O: 333.1710; found: 333.1682.

*2-(4-Trifluoromethylphenyl)-1-[2-(morpholin-4-yl)ethyl]-1H-benzimidazole* (**2l**). Yield: 89%. m.p. 124.3–125.2 °C. FTIR (ATR, cm^−1^): 3052 (aromatic C-H), 2959 (aliphatic C-H), 1618 (C=N), 852 (*para*-substituted benzene). ^1^H-NMR (DMSO-*d*_6_, ppm) δ: 2.15 (4H, s, morpholine -CH_2_-), 2.57 (2H, t, *J* = 6.50 Hz, -CH_2_-), 3.30 (4H, s, morpholine -CH_2_-), 4.45 (2H, t, *J* = 6.50 Hz, -CH_2_-), 7.26–7.33 ( 2H, m, benzimidazole H_5_-H_6_), 7.71 (2H, d, *J* = 7.50 Hz, phenyl H_2_,H′_6_), 7.93–7.95 (2H, m, benzimidazole H_4_,H_7_), 8.09 (2H, d, *J* = 7.50 Hz, phenyl H_3_,H′_5_). ^13^C-NMR (DMSO-*d*_6_, ppm) δ: 42.28, 53.82, 57.55, 66.31, 111.61, 119.89, 122.69, 123.29, 124.62 (*q*, ^1^*J_CF_* = 259.2 Hz), 125.98 (*q*, ^3^*J_CF_* = 3.64 Hz), 130.10 (*q*, ^2^*J_CF_* = 31.9 Hz), 130.66, 135.33, 136.19, 143.10, 152.49. HRMS (*m*/*z*): [M + H]^+^ calcd. for C_20_H_20_N_3_OF_3_: 376.1631; found: 376.1605

*2-(4-Trifluoromethoxyphenyl)-1-[2-(morpholin-4-yl)ethyl]-1H-benzimidazole* (**2m**). Yield: 71%. m.p. 122.5–123.6 °C. FTIR (ATR, cm^−1^): 3055 (aromatic C-H), 2945 (aliphatic C-H), 1614 (C=N), 868 (*para*-substituted benzene). ^1^H-NMR (500 MHz, DMSO-*d*_6_, ppm) δ: 2.12–2.16 (4H, m, morpholine -CH_2_-), 2.55–2.59 (2H, m, -CH_2_-), 3.30 (4H, s, morpholine -CH_2_-), 4.29–4.45 (2H, m, -CH_2_-), 7.22–7.33 (2H, m, benzimidazole H_5_-H_6_), 7.54–7.59 (2H, m, phenyl H_2_,H′_6_), 7.65–7.61 (4H, m, benzimidazole H_4_,H_7_), 7.95–8.00 (2H, m, phenyl H_3_,H′_5_). ^13^C-NMR (DMSO-*d*_6_, ppm) δ: 42.11, 53.79, 57.53, 66.32, 111.48, 119.73, 120.54 (*q*, ^1^*j_C-F_ =* 255.3 Hz), 121.62, 122.55, 123.05, 130.58, 131.95, 136.06, 143.07, 149.52 (*q*, ^2^*j_C-F_ =* 1.5 Hz) 152.68. HRMS (*m*/*z*): [M + H]^+^ calcd. for C_20_H_20_N_3_O_2_F_3_: 392.1580; found: 392.1557

*2-(4-Methylthiophenyl)-1-[2-(morpholin-4-yl)ethyl]-1H-benzimidazole* (**2n**). Yield: 77%. m.p. 153.2–154.1 °C. FTIR (ATR, cm^−1^): 3041 (aromatic C-H), 2951 (aliphatic C-H), 1599 (C=N), 826 (*para*-substituted benzene). ^1^H-NMR (DMSO-*d*_6_, ppm) δ: 2.22 (4H, s, morpholine -CH_2_-), 2.50 (3H, s, -SCH_3_), 2.61 (2H, t, *J* = 6.50 Hz, -CH_2_-), 3.40 (4H, s, morpholine -CH_2_-), 4.41 (2H, t, *J* = 6.50 Hz, -CH_2_-), 7.23–7.26 (2H, m, benzimidazole H_5_-H_6_), 7.43 (2H, d, *J* = 8.50 Hz, phenyl H_2_,H′_6_), 7.64–7.66 (2H, m, benzimidazole H_4_,H_7_), 7.78 (2H, d, *J* = 8.50 Hz, phenyl H_3_,H′_5_). ^13^C-NMR (DMSO-*d*_6_, ppm) δ: 14.77, 42.25, 53.82, 57.44, 66.43, 111.35, 119.53, 122.39, 122.77, 125.94, 127.29, 130.13, 136.17, 140.93, 143.14, 153.55. HRMS (*m*/*z*): [M + H]^+^ calcd. for C_20_H_23_N_3_O_2_S: 354.1635; found: 354.1593.

*2-(1,1’-Biphenyl)-1-[2-(morpholin-4-yl)ethyl]-1H-benzimidazole* (**2o**). Yield: 83%. m.p. 109.4–111.2 °C. FTIR (ATR, cm^−1^): 3049 (aromatic C-H), 2955 (aliphatic C-H), 1610 (C=N), 845 (*para*-substituted benzene), 746 (monosubstituted benzene), 694 (monosubstituted benzene). ^1^H-NMR (DMSO-*d*_6_, ppm) δ: 2.20 (4H, s, morpholine -CH_2_-), 2.51 (2H, t, *J* = 6.50 Hz, -CH_2_-), 3.48 (4H, s, morpholine -CH_2_-), 4.41 (2H, t, *J* = 6.50 Hz, -CH_2_-), 7.25–7.33 (5H, m), 7.40–7.53 (5H, m), 7.66–7.88 (2H, m), 7.67–7.88 (6H, m). ^13^C-NMR (DMSO-*d*_6_, ppm) δ: 42.25, 53.82, 57.53, 66.42, 111.38, 119.69, 122.47, 122.88, 127.24, 128.39, 129.51, 130.19, 130.35, 136.23, 139.69, 141.55, 143.29, 153.63. HRMS (*m*/*z*): [M + H]^+^ calcd. for C_25_H_25_N_3_O: 384.2070; found: 384.2047.

### 3.3. Antimicrobial Assay

Microbiological studies were performed according to CLSI reference M07-A9 broth microdilution method for bacterial strains [58] and EUCAST definitive method EDef 7.1 for *Candida* species [59]. Synthesized compounds were tested for their in vitro growth inhibitory activity against *Escherichia coli* ATCC 35218, *Escherichia coli* ATCC 25922, *Staphylococcus aureus*, ATCC 25923, *Pseudomona aeuroginosa* ATCC 27853, *Enterococcus faecalis* ATCC29212, *Klebsiella pneumoniae* ATCC700603 *Listeria monocytogenes* ATCC7644, *Candida albicans* ATCC90028, *Candida glabrata* ATCC 90030, *Candida krusei* ATCC 6258, *Candida parapsilosis* ATCC 22019.

The cultures were obtained from Mueller-Hinton broth (Difco) for the bacterial strains and the yeasts were maintained in Roswell Park Memorial Institute (RPMI) medium, after an overnight incubation at 37 °C for both. The inocula of the test microorganisms were adjusted to match an equivalent turbidity of a 0.5 McFarland standard, which was determined using a spectrophotometer; the final inoculum size was determined to be 0.5–2.5 × 10^5^ cfu/mL for antibacterial and antifungal assays. The tests were carried out for both mediums at pH = 7 and two-fold serial dilutions were applied. The last well on the microplates, which was containing only the inoculated broth, was kept as control, and the last well with no growth of microorganism was recorded to represent the minimum inhibitory concentration (MIC_50_) in μg/mL. For both of the antibacterial and antifungal assays, the test compounds and reference drugs were firstly dissolved in DMSO, and further dilutions were performed to the desired concentrations of 1000, 500, 250, 125, 62.5, 31.25, 15.6, 7.8, 3.9 and 1.95 μg/mL using Mueller–Hinton broth and RPMI medium. The completed plates were incubated for 24 h, and at the end of the incubation, resazurin (20 µg/mL) was added into each well to control the growth in the wells. Final plates including microorganism strains were incubated for 2 h. MIC_50_ values were determined using microplate reader at 590 nm excitation and 560 nm emission wavelengths; MIC_50_ readings were performed twice for entire compounds. Chloramphenicol and ketoconazole were used as reference drugs.

### 3.4. COX-1 and COX-2 Inhibition Assays

Inhibitory potency of the compounds on COX-1 and COX-2 enzymes was determined by using fluorimetric COX-1 and COX-2 inhibition screening kits from BioVision (Milpitas, CA, USA). The experimental protocol as described in the guides provided by the supplier was followed [51,52]. All of the pipetting were performed using a robotized system to increase speed, accuracy and precision. Fluorescence intensities of the samples were kinetically measured at 25 °C for 5–10 min by monitoring emissions at 587 nm after excitation at 535 nm. Two appropriate points (T1 and T2) in the linear range of the plot were chosen and the corresponding fluorescence values (RFU1 and RFU2) were obtained. The slope for all samples including Enzyme Control (EC) was calculated by dividing the net ΔRFU (RFU2 − RFU1) values by the time ΔT (T2 − T1). Percentage of relative inhibition was calculated by using the following equation:

% Relative inhibition = (Slope of EC − Slope of S/Slope of EC) × 100

where S is the term representing the tested compound [51,52]. The initial in vitro assays were performed at 10^−3^ M and 10^−4^ M concentrations for all of the test compounds; the ones which showed inhibition above 50% were assayed by the same protocol within a wider range of concentrations (10^−5^ M and 10^−9^ M) to determine their IC_50_ against COX-1 and COX-2 enzymes. The IC_50_ values were calculated using the plots of the enzyme activity against concentrations by applying regression analyses.

### 3.5. AChE Inhibition Assay

Inhibition potency of the test compounds against AChE was determined using Ellman’s method [53]. Enzyme solutions were prepared in gelatin solution (1%, *w*/*v*), at the concentration of 2.5 units/mL. The synthesized compounds, and donepezil, which was used for reference, were prepared at 10^−3^ M and 10^−4^ M concentrations using 2% DMSO solution for initial measurements. AChE solution (20 µL/well) and test solution (20 µL/well) were added to phosphate buffer solution (140 µL/well, pH 8.0 ± 0.1), and incubated at 25 °C for 5 min. The reaction was started by addition of the chromogenic reagent 5,5-dithio-bis(2-nitrobenzoic acid) (DTNB, 20 µL/well, 10 mM) and the substrates acetylthiocholine iodide (ATCI, 10 µL/well, 75 µM) to the enzyme-inhibitor mixture. The production of the yellow anion was recorded by multimode microplate reader for 10 min at 412 nm. As a control, an identical solution of the enzyme without the inhibitor was processed. The control and the inhibitor readings were corrected with blank-readings. All processes were assayed in four independent determinations.

### 3.6. MAO Inhibition Assay

Enzyme activity assays were performed according to the procedure reported by Matsumoto et al. [54] with slight modifications. In order to prevent the destructive effect of light on MAO enzymes, the assays were performed in 96-well black plates. Phosphate buffer solution (0.1 M, pH = 7.4) was used for preparation of stock solutions (5 mg/mL) of both human recombinant MAO-A and MAO-B enzymes. Enzyme stock solutions were further diluted using assay buffer to achieve a final concentration of 0.006 mg/mL for MAO-A and 0.015 mg/mL for MAO-B. Kynuramine was dissolved in sterile deionized water to prepare stock solution (25 mM) and then diluted using assay buffer to get final concentration of 40 μM for MAO-A enzyme and 20 μM for MAO-B enzyme. Synthesized compounds and reference drugs, which were moclobemide for MAO-A and selegiline for MAO-B, were diluted to 10^−3^ M and 10^−4^ M concentrations (100 μL/well) using 2% DMSO. The addition of the test compound solution was followed by addition of either MAO-A or MAO-B enzyme (50 μL/well) solutions. After 10 min of incubation at 37 °C, kynuramine (50 μL/well) was added and enzyme-substrate reaction was initiated. The plate was incubated for 20 min at 37 °C and then reaction was terminated by addition of 2 N NaOH (75 μL/well). The fluorimetric reads from the top was performed at 310 nm excitation and 380 nm emission wavelengths.

### 3.7. Determination of Cytotoxicity

Cytotoxicity was tested using NIH/3T3 mouse embryonic fibroblast cell line (ATCC^®^ CRL-1658™, London, UK). NIH/3T3 cells were incubated according to the supplier’s recommendations. NIH/3T3 cells were seeded as 1 × 10^4^ cells into each well of 96-well plates. MTT assay was performed as previously described [60,61]. The compounds were tested between 1 mM and 0.000316 mM concentrations (1.0, 0.316, 0.10, 0.0316, 0.01, 0.00316, 0.001, 0.000316 mM). The IC_50_ values were determined by plotting a dose-response curve of inhibition % versus tested concentrations of the compound [62].

### 3.8. Determination of Genotoxicity

The genotoxicity of selected compounds (**2b**, **2j**, **2m**) was determined by Ames assay using Ames MPF 98/100 mutagenicity assay sample kit (Xenometrix AG, Allschwil, Switzerland) as previously described elsewhere [63]. *Salmonella typhimurium* strains, TA98 (frameshift mutations) and TA100 (base-pair substitutions) were used in the assay. The concentration of the compounds was between 16 and 5000 μg/mL, in accordance with the guideline [64]. Compounds were prepared in six different concentrations (5.0, 2.5, 1.25, 0.625, 0.3125, 0.156 mg/mL) in DMSO. Mutagenic potential was determined in the absence or presence of Aroclor™-1254 induced male Sprague–Dawley rat liver microsomal enzyme (S9) mix (Xenometrix AG). Positive controls without S9 mix were 2-nitrofluorene (2.0 μg/mL) and 4-nitroquinoline *N*-oxide (0.1 μg/mL), whereas 1.0 μg/mL and 2.5 μg/mL of 2-aminoanthracene solutions were used as positive controls with S9 against TA98 and TA100, respectively. Solvent control was prepared with 4% DMSO. The end of an experiment was determined by the change of the indicator medium colour to yellow, which was originating from the decrease of pH due to revertant bacteria. Yellow wells were counted as positive, and compared with the negative control. Fold induction over the negative control and fold induction over the baseline were calculated (Fold induction over the negative control is accepted as the ratio of the mean number of positive wells for the dose concentration divided by the mean number of positive wells for the zero dose (negative) control. Fold induction over the baseline is accepted as the ratio of the mean number of positive wells for the dose concentration divided by zero dose baseline. The zero-dose baseline is obtained by adding one standard deviation to the mean number of positive wells of the zero dose control. If the baseline is less than 1, the value is set to 1 for calculation).

Mutagenicity was determined according to the criteria reported previously [55]. For a baseline value of ≤3, significant increases between 2 and 3-fold of the baseline were classified as weak mutagen, and increases ≥3-fold of the baseline were classified as mutagen. For a baseline value of >3, significant increases between 1.5 and 2.5-fold of the baseline were classified as weak mutagen, and increases ≥2.5-fold of the baseline, were classified as mutagen. As a rule, at least two adjacent doses with significant increases or a significant increase at the highest dose level should be observed for a mutagenic compound. All of the doses were compared according to Student’s *t*-test at *p* < 0.05 for statistical significance. Compounds that did not possessed any of the characteristics mentioned above were classified as non-mutagenic.

### 3.9. Prediction of ADME Properties and Drug Likeness

Some physicochemical parameters, which were used to evaluate ADME properties of the compounds (**2a–2o**) were calculated via online molecular property and bioactivity score calculation software of Molinspiration Cheminformatics (ver. 2017, Molinspiration Cheminformatics, Slovensky Grob, Slovak Republic) [56]. Drug-likeness score of the compounds was assigned by an online drug-likeness and molecular property prediction software (ver. 2017, Molsoft, San Diego, CA, USA) [57].

## 4. Conclusions

In the present study, synthesis and structural verification of some novel benzimidazole-morpholine compounds were described, and their biological and pharmaceutical value was evaluated by applying multiple biological, pharmacological and toxicological tests. Briefly, it can be concluded that the synthesized compounds did not exceptionally inhibit AChE, MAO-A and MAO-B enzymes. On the other hand, compounds **2b**, **2c**, **2e** and **2m** presented acceptable antibacterial activity especially against *Pseudomona aeuroginosa* ATCC 27853. Furthermore, a promising activity was observed for compounds **2b** and **2j** against COX-1 and COX-2 enzymes, meanwhile compound **2b** was the most active derivative in both antimicrobial and COX inhibition assays. It is a common knowledge that microbial infections generally accompanied with fever and inflammation; thus, among the compounds in the series 2-(4-methoxyphenyl)-1-[2-(morpholin-4-yl)ethyl]-1*H*-benzimidazole (**2b**) seems to be most powerful candidate for the treatment of a such disease, with dual activity against *Pseudomona aeuroginosa* ATCC and COX enzymes.

## Figures and Tables

**Table 1:** Antimicrobial activity results of test compounds **2a–2o** (MIC_50_ values are given in mg/mL).

Compound	A	B	C	D	E	F	G	H	I	J	K
**2a**	>1	>1	>1	>1	>1	>1	>1	>1	>1	>1	>1
**2b**	>1	>1	>1	0.0156	>1	>1	>1	>1	>1	>1	>1
**2c**	>1	>1	>1	0.125	>1	>1	>1	>1	>1	>1	>1
**2d**	>1	>1	>1	0.500	>1	>1	>1	>1	>1	>1	>1
**2e**	>1	>1	>1	0.250	>1	>1	>1	>1	>1	>1	>1
**2f**	>1	>1	>1	0.500	>1	>1	>1	>1	>1	>1	>1
**2g**	>1	>1	>1	>1	>1	>1	>1	>1	>1	>1	>1
**2h**	>1	>1	>1	>1	>1	>1	>1	>1	>1	>1	>1
**2i**	>1	>1	>1	>1	>1	>1	>1	>1	>1	>1	>1
**2j**	>1	>1	>1	>1	>1	>1	>1	>1	>1	>1	>1
**2k**	>1	>1	>1	>1	>1	>1	>1	>1	>1	>1	>1
**2l**	>1	>1	>1	>1	>1	>1	>1	>1	>1	>1	>1
**2m**	>1	>1	>1	0.125	>1	>1	>1	>1	>1	>1	>1
**2n**	>1	>1	>1	0.500	>1	>1	>1	>1	>1	>1	>1
**2o**	>1	>1	>1	0.500	>1	>1	>1	>1	>1	>1	>1
**Ref-1**	>1	>1	>1	>1	>1	>1	>1	0.0039	0.0312	0.0019	0.0625
**Ref-2**	0.0312	>1	0.0156	0.250	0.0625	0.250	0.0312	>1	>1	>1	>1

**A**: *Escherichia coli* ATCC 35218; **B**: *Escherichia coli* ATCC 25922; **C**: *Staphylococcus aureus* ATCC 25923; **D**: *Pseudomona aeuroginosa* ATCC 27853; **E**: *Enterococcus faecalis* ATCC 29212; **F**: *Klebsiella pneumoniae* ATCC 700603; **G**: *Listeria monocytogenes* ATCC 7644; **H**: *Candida albicans* ATCC 90028; **I**: *Candida glabrata* ATCC 90030; **J**: *Candida krusei* ATCC 6258; **K**: *Candida parapsilopsis* ATCC 22019; **Ref-1**: Ketoconazole; **Ref-2**: Chloramphenicol.

**Table 2:** Inhibitory potencies of selected test compounds (**2a**, **2b**, **2f**, **2h**, **2j** and **2m**) at lower concentrations against COX-1 and COX-2 enzymes.

Comp.	COX 1 Inhibition %						COX 2 Inhibition %					
	10^−5^ M	10^−6^ M	10^−7^ M	10^−8^ M	10^−9^ M	IC_50_ µM	10^−5^ M	10^−6^ M	10^−7^ M	10^−8^ M	10^−9^ M	IC_50_ µM
**2a**	47.39 ± 1.08	40.12 ± 0.92	35.68 ± 0.97	30.46 ± 0.83	28.81 ± 0.79	56.08	43.72 ± 1.07	40.32 ± 1.10	38.26 ± 0.92	36.20 ± 0.87	32.44 ± 0.91	58.66
**2b**	59.37 ± 1.24	42.75 ± 1.02	37.08 ± 0.92	26.77 ± 0.87	19.56 ± 0.76	8.096	57.85 ± 1.28	43.70 ± 1.12	38.12 ± 0.97	27.45 ± 0.87	21.35 ± 0.74	8.369
**2f**	45.38 ± 0.99	42.08 ± 0.87	34.23 ± 0.76	32.68 ± 0.78	29.88 ± 0.68	43.23	46.98 ± 1.13	40.22 ± 1.24	35.62 ± 0.91	31.46 ± 0.84	28.74 ± 0.63	44.92
**2h**	46.29 ± 1.09	40.88 ± 0.99	35.62 ± 0.81	30.77 ± 0.93	24.23 ± 0.72	42.97	47.54 ± 1.08	42.60 ± 1.16	31.75 ± 0.99	28.35 ± 0.82	23.04 ± 0.68	51.19
**2j**	51.41 ± 1.15	44.45 ± 1.07	37.66 ± 0.76	27.46 ± 0.82	23.44 ± 0.61	14.18	52.54 ± 1.35	43.40 ± 1.27	33.98 ± 1.06	26.85 ± 0.86	20.54 ± 0.76	13.09
**2m**	47.36 ± 1.16	42.02 ± 0.88	36.65 ± 0.92	26.19 ± 0.79	20.01 ± 0.62	21.94	46.11 ± 1.13	41.23 ± 1.05	34.48 ± 0.97	28.69 ± 0.83	22.30 ± 0.52	22.66
**Ref-1**	74.67 ± 1.28	45.08 ± 1.16	28.23 ± 0.91	22.66 ± 0.82	14.76 ± 0.60	2.435	69.51 ± 1.28	36.38 ± 0.91	29.42 ± 0.84	24.03 ± 0.76	17.08 ± 0.58	5.327
**Ref-2**	68.39 ± 1.26	41.91 ± 0.98	29.05 ± 0.92	20.37 ± 0.81	18.84 ± 0.63	3.810	78.69 ± 1.19	48.28 ± 1.09	32.97 ± 0.96	21.30 ± 0.88	14.12 ± 0.61	1.683

**Ref-1:** Ibuprofen; **Ref-2:** Nimesulide.

**Table 3:** IC_50_ values of selected test compounds (**2b**, **2j** and **2m**) against NIH3T3 cell lines and results of Ames MPF test.

Comp.	Cytotoxicity NIH3T3 Cells IC_50_ (µM)	Concentration (mg/mL) for Ames Test	REVERTANTS Fold Increase (over Baseline)			
			TA98		TA100	
			S9+	S9−	S9+	S9−
**2b**	316	0.156	0.16	0.69	0.06	1.31 ***
		0.3125	0.32	0.75	0.52	1.37 ***
		0.625	0.00	0.81	0.45	0.69
		1.25	0.48	0.40	0.26	0.62
		2.5	0.80	0.40	0.26	0.25 ***
		5	1.27	0.75	0.13	0.44 *
**2j**	316	0.156	0.25	0.60	0.15	0.80
		0.3125	0.12 *	0.94	0.00	1.00
		0.625	0.62	0.68	0.15	0.40
		1.25	0.74	0.60	0.38	0.67
		2.5	0.12	0.94	0.31	0.67 ***
		5	0.74 *	0.09 ***	0.15	1.20
**2m**	100	0.156	0.46	0.95	0.10	0.61
		0.3125	0.62	0.70	0.10	0.88
		0.625	0.77	0.57	0.88	0.47
		1.25	0.46	1.08 *	0.19	0.47
		2.5	1.23	0.82	0.49	0.54
		5	0.31	1.08 *	0.29	0.61

* *t*-test *p* value (unpaired, one-sided) < 0.05. *** *t-*test *p* value (unpaired, one-sided) < 0.001.

**Table 4:** Some physicochemical parameters of test compounds **2a–2o** and reference drugs used in prediction of ADME profiles.

Compound	MW	logP	tPSA	nON	nOHNH	Volume	Vio	DLS
**2a**	321.42	3.94	30.30	4	0	308.95	0	0.74
**2b**	337.42	3.54	39.53	5	0	317.94	0	1.12
**2c**	341.84	4.17	30.30	4	0	305.93	0	1.21
**2d**	325.39	3.65	30.30	4	0	297.32	0	1.10
**2e**	349.48	5.00	30.30	4	0	342.34	1	1.15
**2f**	375.39	4.38	30.30	4	0	323.69	0	0.69
**2g**	386.29	4.30	30.30	4	0	310.28	0	0.89
**2h**	378.52	4.34	33.54	5	0	371.90	0	0.61
**2i**	350.47	3.59	33.54	5	0	338.30	0	0.59
**2j**	351.45	3.92	39.53	5	0	334.74	0	1.01
**2k**	332.41	3.24	54.09	5	0	309.25	0	0.63
**2l**	413.52	5.14	39.53	5	0	389.59	1	0.92
**2m**	391.39	4.46	39.53	5	0	332.67	0	0.88
**2n**	353.49	3.92	30.30	4	0	327.08	0	0.92
**2o**	383.50	5.28	30.30	4	0	363.80	1	0.68
**Donepezil**	379.50	4.10	38.78	4	0	367.89	0	1.76
**Moclobemide**	268.74	1.69	41.57	4	0	240.70	0	1.36
**Selegiline**	187.29	2.64	3.24	1	0	202.64	0	1.03
**Chloramphenicol**	323.13	0.73	115.38	7	3	249.16	0	0.89
**Ketoconazole**	531.44	3.77	69.08	8	0	452.47	1	1.32
**Ibuprofen**	206.28	3.46	37.30	2	1	211.19	0	0.96
**Nimesulide**	308.31	2.81	101.23	7	1	248.17	0	−1.40

**MW:** Molecular weight; **tPSA:** Topological polar surface are; **nON:** Number of hydrogen donors; **nOHNH:** Number of hydrogen acceptors; **Vio:** Violence; **DLS:** Drug likeness score.

## Supplementary Materials

The following are available online. Table S1: Percentage of inhibition of test compounds **2a–2o** against AChE, MAO-A and MAO-B enzymes, Table S2: Inhibitory potencies of test compounds **2a–2o** at higher concentrations against COX-1 and COX-2 enzymes. The ^13^C-NMR, ^1^H-NMR, FTIR and HRMS spectrums of compounds **2a–2o**.
