# Supplementary material for: Pharmacological and Toxicological Screening of Novel Benzimidazole-Morpholine Derivatives as Dual-Acting Inhibitors
Source: Molecules. 2017 Aug 19;22(8):1374. doi: 10.3390/molecules22081374 (PMC6152416; doi:10.3390/molecules22081374)
Supplement: Supplementary file 1 [file molecules-22-01374-s001.pdf]

Table S1. Percentage of inhibition of test compounds **2a-2o** against AChE, MAO-A and MAO-B enzymes.

**Compound**	**AChE Inhibition (%)**		**MAO-A Inhibition (%)**		**MAO-B Inhibition (%)**	
	**10^{-3} M**	**10^{-4} M**	**10^{-3} M**	**10^{-4} M**	**10^{-3} M**	**10^{-4} M**
**2a**	21.80 ±0.95	21.80 ±0.95	24.29 ±1.08	19.15 ±0.87	25.27 ±0.96	16.63 ±0.74
**2b**	5.19 ±0.21	5.19 ±0.21	29.44 ±0.88	24.61 ±0.79	32.44 ±1.06	31.75 ±0.92
**2c**	5.48 ±0.19	5.48 ±0.19	31.58 ±1.12	26.17 ±1.06	41.12 ±1.21	33.54 ±1.07
**2d**	16.20 ±0.74	16.20 ±0.74	39.79 ±1.08	33.11 ±0.89	45.29 ±1.20	38.96 ±0.93
**2e**	17.51 ±0.76	17.51 ±0.76	24.29 ±0.92	17.31 ±0.80	35.47 ±1.30	26.65 ±1.15
**2f**	15.75 ±0.64	15.75 ±0.64	32.14 ±0.90	20.65 ±0.86	39.32 ±1.08	31.14 ±1.00
**2g**	13.04 ±0.52	13.04 ±0.52	30.85 ±0.94	22.29 ±0.97	38.63 ±1.11	30.87 ±0.84
**2h**	10.49 ±0.48	10.49 ±0.48	36.71 ±1.03	23.16 ±0.94	45.58 ±1.27	36.16 ±1.20
**2i**	7.54 ±0.33	7.54 ±0.33	26.91 ±0.92	19.20 ±0.77	22.17 ±0.95	14.43 ±0.60
**2j**	7.20 ±0.28	7.20 ±0.28	28.47 ±1.04	19.42 ±0.85	41.73 ±1.77	32.44 ±1.16
**2k**	20.45 ±0.98	20.45 ±0.98	31.99 ±1.00	26.86 ±0.93	43.22 ±1.08	34.29 ±1.09
**2l**	33.88 ±1.09	33.88 ±1.09	38.51 ±1.20	30.16 ±1.08	47.66 ±1.17	38.09 ±0.95
**2m**	32.90 ±0.99	32.90 ±0.99	34.29 ±1.07	16.39 ±0.72	25.84 ±0.98	12.43 ±0.42
**2n**	12.21 ±0.58	12.21 ±0.58	32.73 ±1.18	24.05 ±0.92	34.75 ±0.85	32.24 ±0.89
**2o**	6.33 ±0.29	6.33 ±0.29	31.85 ±1.05	26.19 ±0.86	31.12 ±0.91	23.54 ±0.87
**Ref-1**	99.48 ±1.92	99.48 ±1.92	-	-	-	-
**Ref-2**	-	-	94.12 ±2.76	82.14 ±2.69	-	-
**Ref-3**					98.94 ±2.06	94.73 ±1.97

Ref-1: Donepezil; **Ref-2:** Moclobemide; **Ref-3:** Selegiline.

Table S2. Inhibitory potencies of test compounds **2a-2o** at higher concentrations against COX-1 and COX-2 enzymes.

Compound	COX-1 Inhibition (%)		COX-2 Inhibition (%)	
	10^{-3} M	10^{-4} M	10^{-3} M	10^{-4} M
**2a**	**75.08 ±1.28**	**71.23 ±1.28**	**58.28 ±1.16**	**56.23 ±1.25**
**2b**	**90.12 ±1.39**	**87.62 ±1.19**	**79.41 ±1.24**	**84.23 ±1.24**
**2c**	24.55 ±0.94	22.15 ±0.82	17.85 ±0.67	20.99 ±0.72
**2d**	31.87 ±0.82	29.36 ±0.90	21.08 ±0.88	28.46 ±0.69
**2e**	26.47 ±0.83	19.88 ±0.82	14.26 ±0.52	22.61 ±0.61
**2f**	**75.37 ±1.20**	**79.08 ±1.37**	**61.23 ±1.25**	**60.20 ±1.18**
**2g**	29.47 ±0.86	21.74 ±0.83	16.30 ±0.42	19.03 ±0.74
**2h**	**75.21 ±1.06**	**78.21 ±1.27**	**56.20 ±1.08**	**57.08 ±1.00**
**2i**	30.08 ±1.05	22.62 ±0.86	18.47 ±0.51	25.76 ±1.08
**2j**	**85.07 ±1.49**	**88.68 ±1.47**	**80.66 ±1.26**	**79.23 ±1.37**
**2k**	28.63 ±0.86	28.13 ±0.86	26.39 ±0.76	20.46 ±0.73
**2l**	36.87 ±0.90	33.46 ±0.84	21.00 ±0.71	34.62 ±0.70
**2m**	**79.15 ±1.20**	**78.61 ±1.26**	**64.22 ±1.09**	**59.67 ±1.24**
**2n**	39.42 ±1.18	32.78 ±0.86	30.08 ±0.88	33.46 ±0.97
**2o**	27.63 ±0.96	34.00 ±0.89	30.76 ±0.62	21.48 ±0.81
**Ref-1**	98.15 ±1.56	98.23 ±1.49	88.15 ±1.37	89.36 ±1.43
**Ref-2**	96.53 ±1.38	97.82 ±1.32	89.57 ±1.28	84.22 ±1.23

Ref-1: Ibuprofen; **Ref-2:** Nimesulide.

^{13}C-NMR spectrum of *2-(4-Methylphenyl)-1- [2-(morpholin-4-yl)ethyl]-1H-benzimidazole* (***2a***)

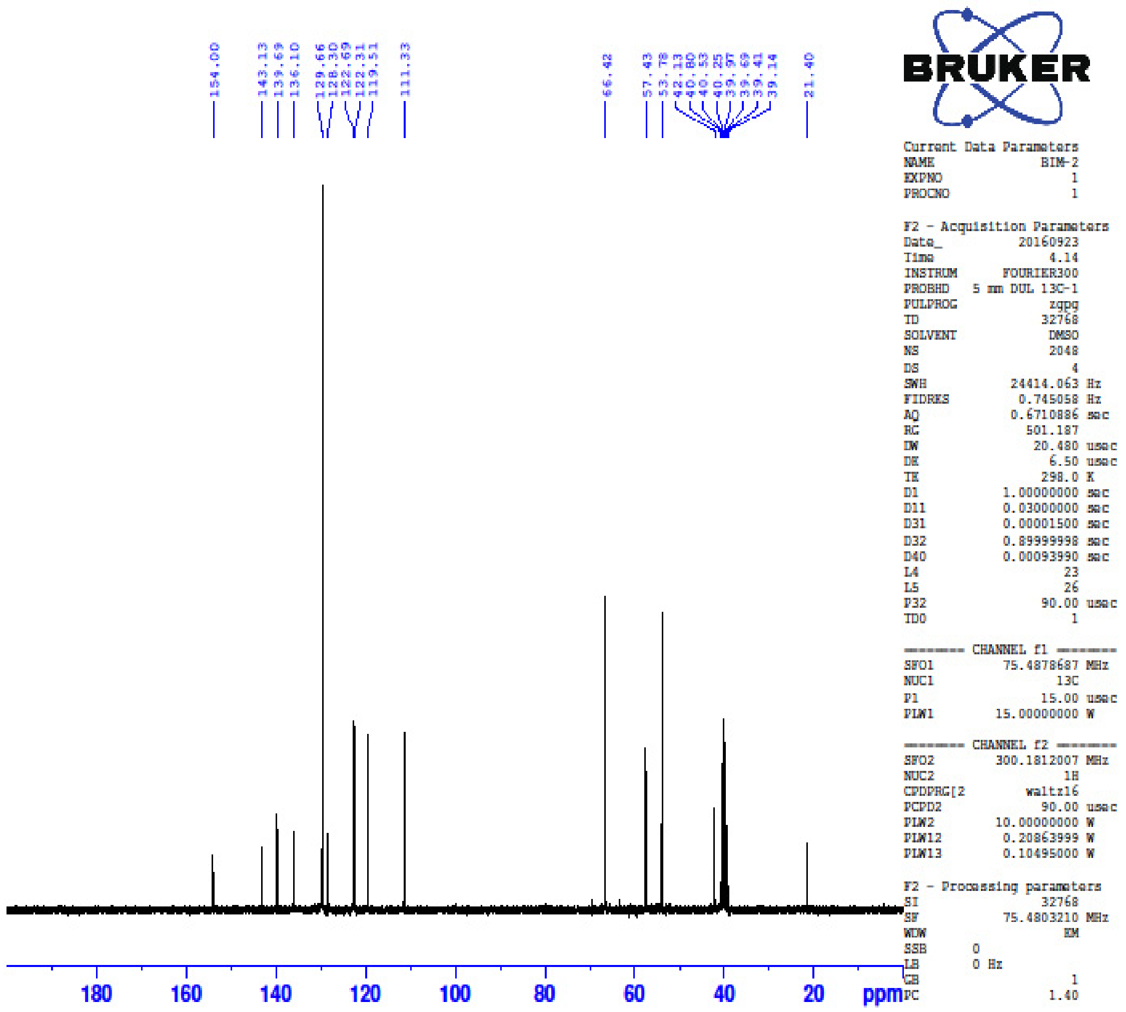

^{1}H-NMR spectrum of *2-(4-Methylphenyl)-1- [2-(morpholin-4-yl)ethyl]-1H-benzimidazole (**2a**)*

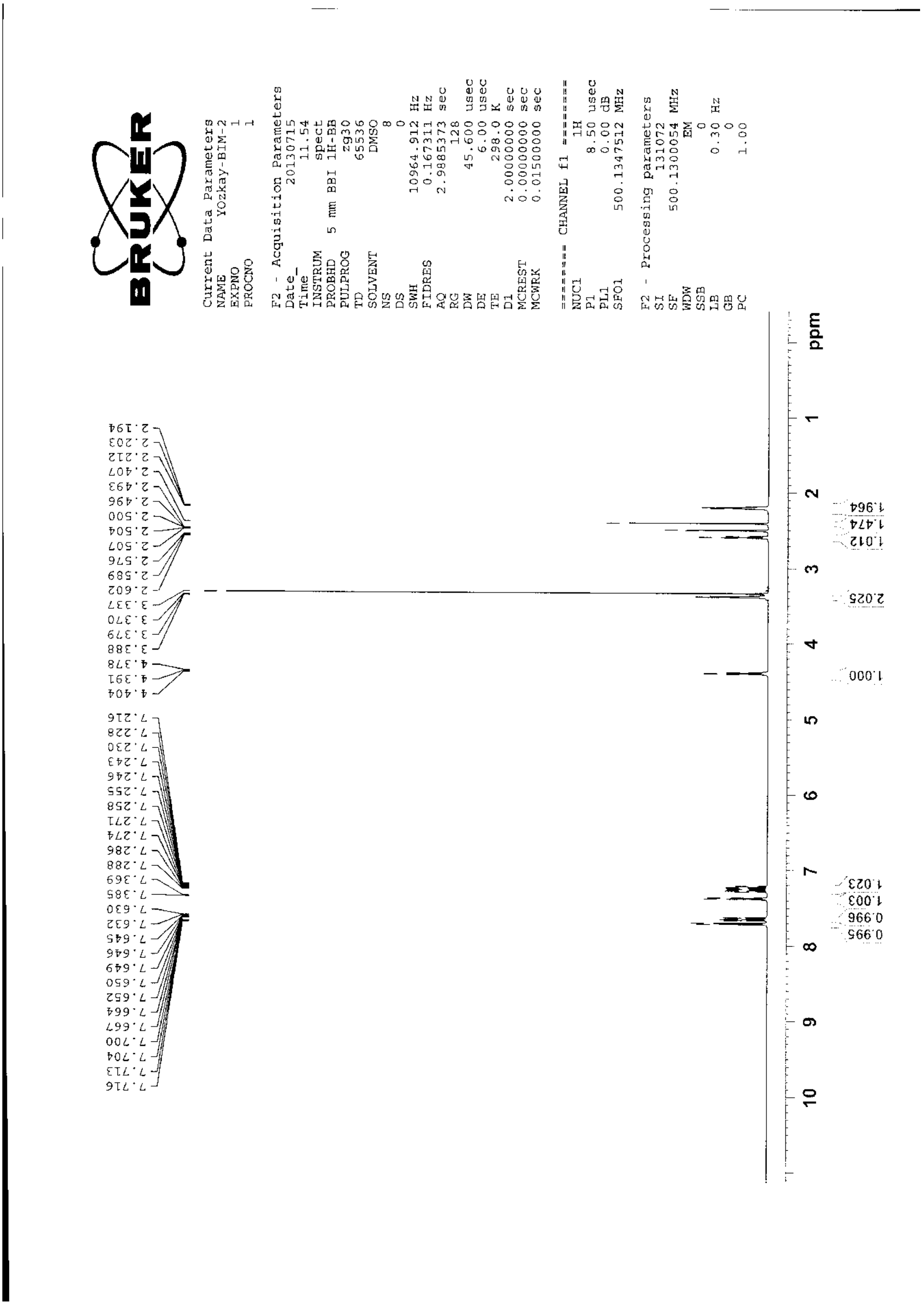

FTIR spectrum of *2-(4-Methylphenyl)-1- [2-(morpholin-4-yl)ethyl]-1H-benzimidazole (**2a**)*

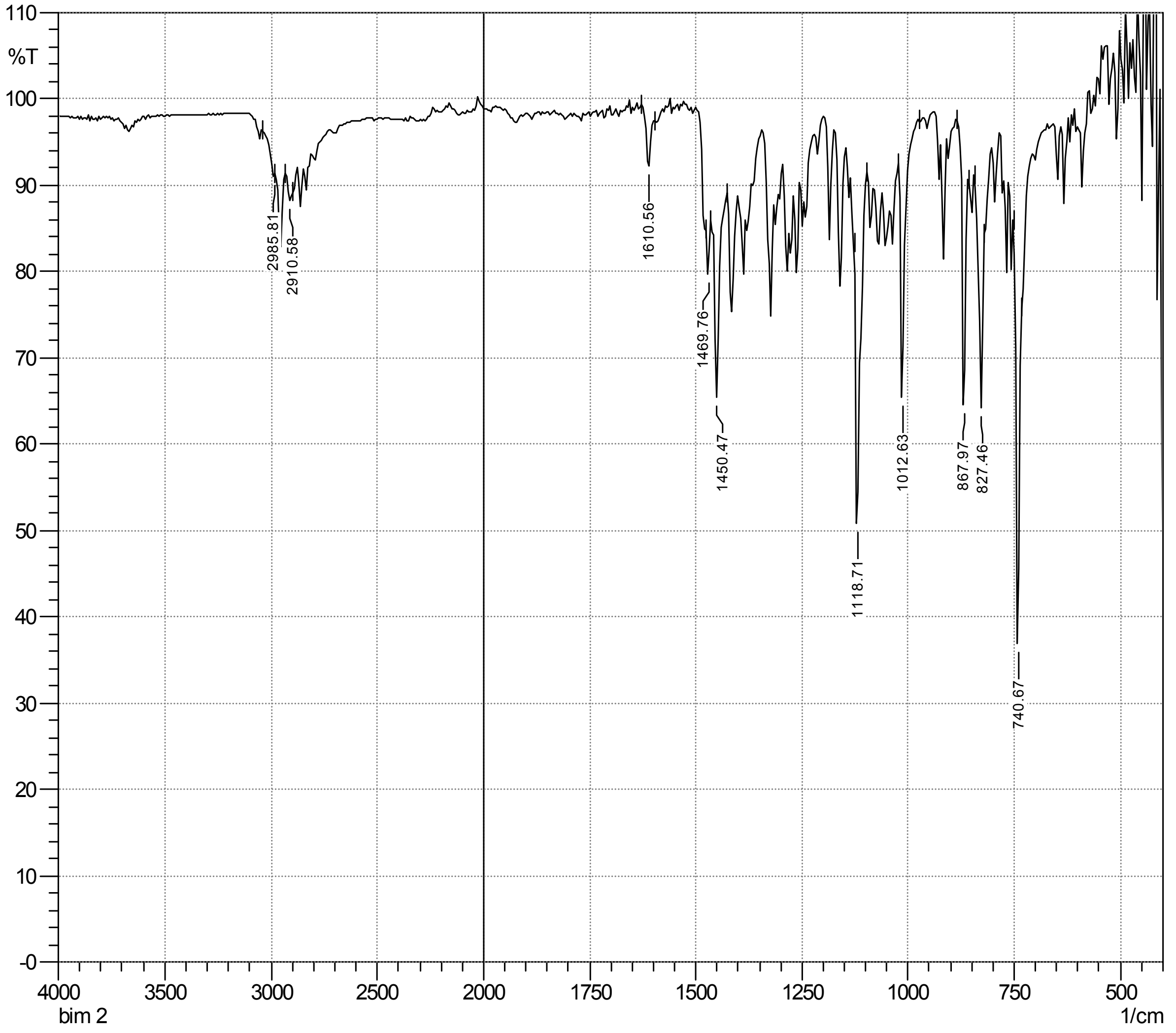

HRMS spectrum of *2-(4-Methylphenyl)-1- [2-(morpholin-4-yl)ethyl]-1H-benzimidazole* (***2a***)

Data File: C:\ LabSolutions\ Data\Analiz\Bim serisi\ Bim-2_09.lcd

Elmt	Val.	Min	Max	Elmt	Val.	Min	Max	Use Adduct
H	1	14	30	O	2	1	5	H
C	4	12	30	S	2	0	0	
N	3	3	4	Cl	1	0	0	

Error Margin (ppm): 10
HC Ratio: unlimited
Max Isotopes: 3
MSn Iso RI (%): 10.00

DBE Range: -2.0 - 1000.0
Apply N Rule: yes
Isotope RI (%): 1.00
MSn Logic Mode: AND

Electron Ions: both
Use MSn Info: no
Isotope Res: 10000
Max Results: 500

Event#: 1 MS(E+) Ret. Time : 4.613 -> 4.640 Scan# : 693 -> 697

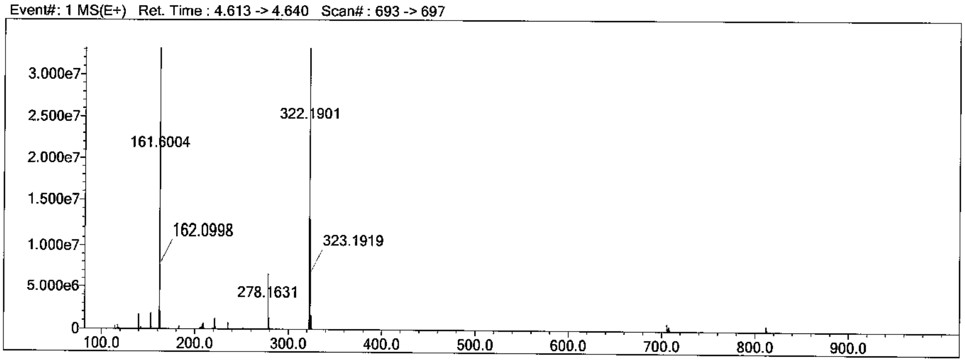

Measured region for 322.1901 m/z

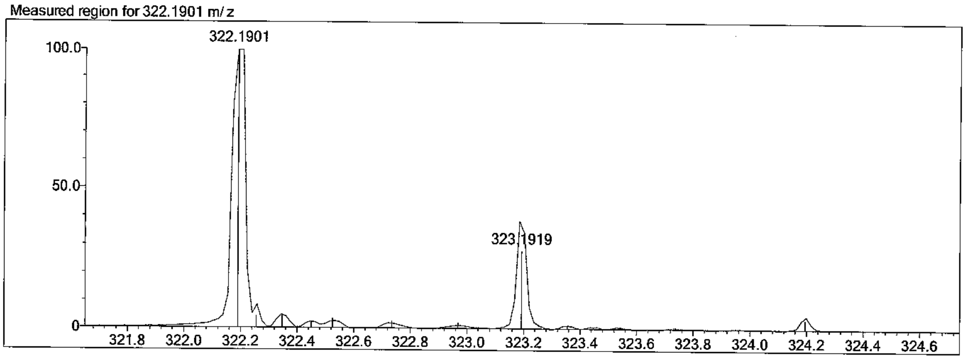

C20 H23 N3 O [M+H]+ : Predicted region for 322.1914 m/z

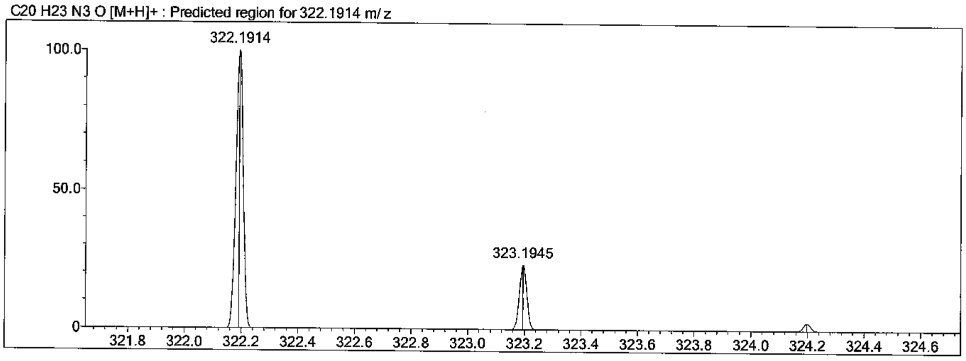

Rank	Score	Formula (M)	Ion	Meas. m/z	Pred. m/z	Df. (mDa)	Df. (ppm)	Iso	DBE
1	65.89	C20 H23 N3 O	[M+H]+	322.1901	322.1914	-1.3	-4.03	71.29	11.0

^{13}C-NMR spectrum of *2-(4-Methoxyphenyl)-1-[2-(morpholin-4-yl)ethyl]-1H-benzimidazole* (***2b***)

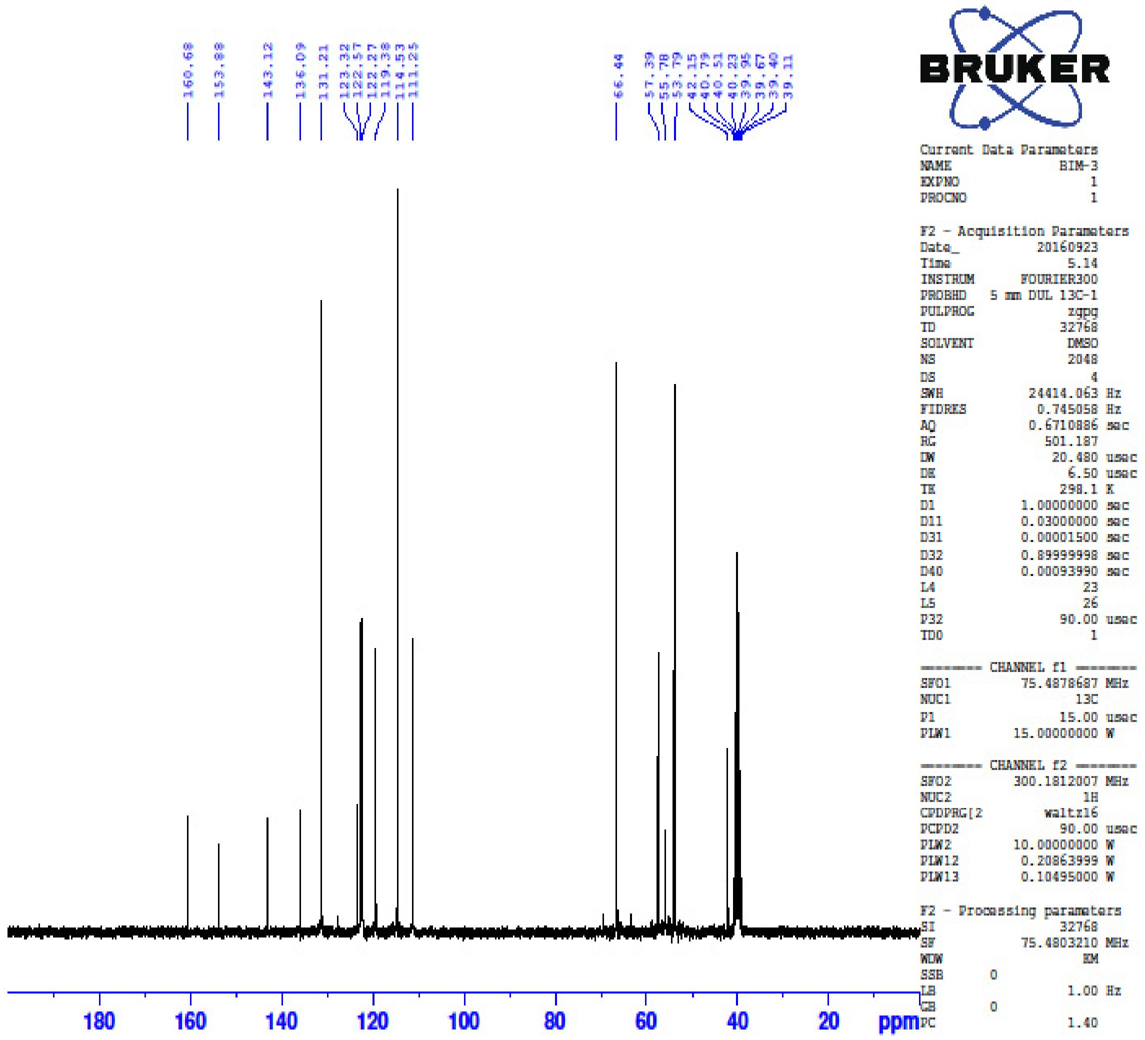

^{1}H-NMR spectrum of *2-(4-Methoxyphenyl)-1-[2-(morpholin-4-yl)ethyl]-1H-benzimidazole* (***2b***)

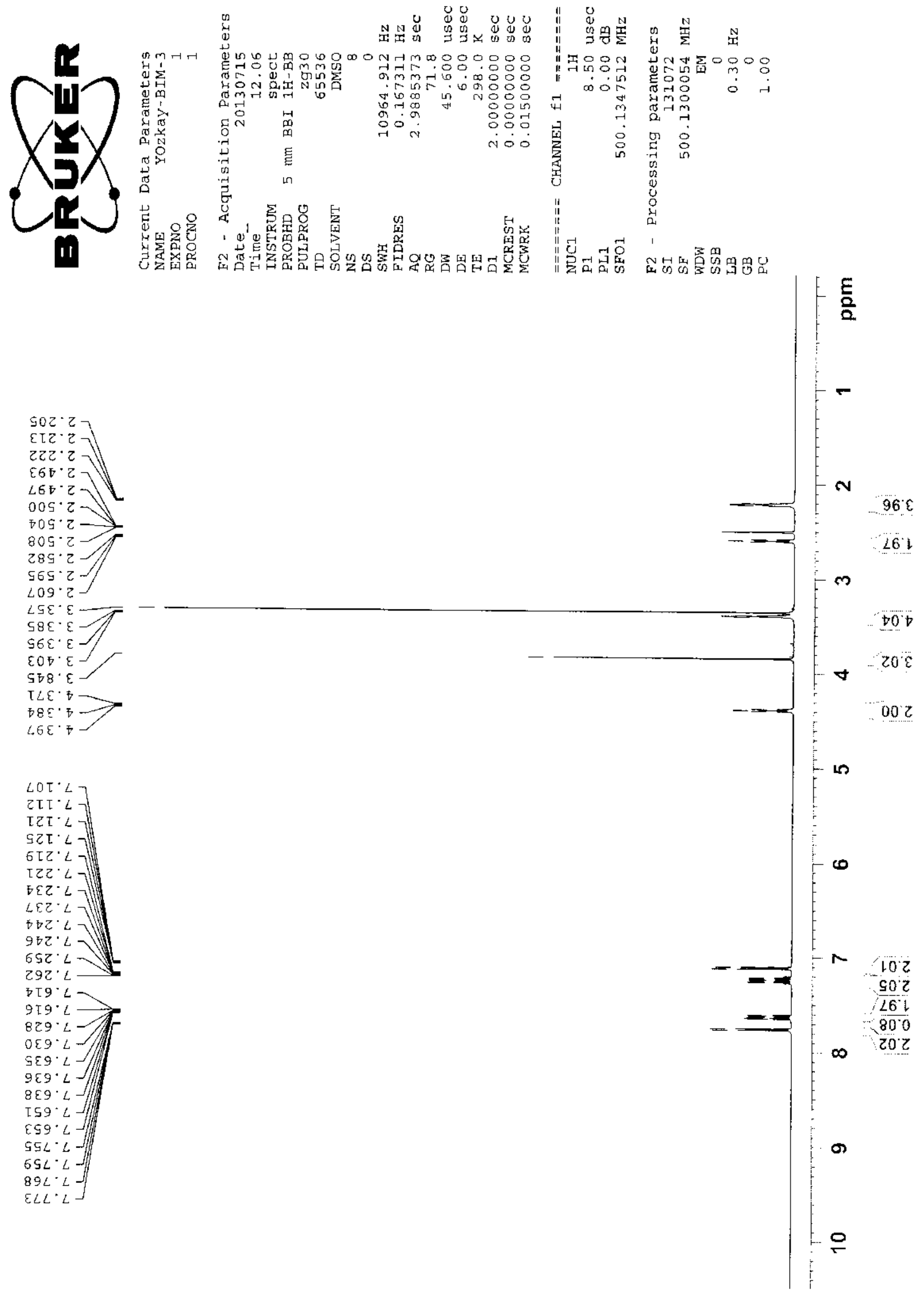

FTIR spectrum of *2-(4-Methoxyphenyl)-1-[2-(morpholin-4-yl)ethyl]-1H-benzimidazole* ***(2b)***

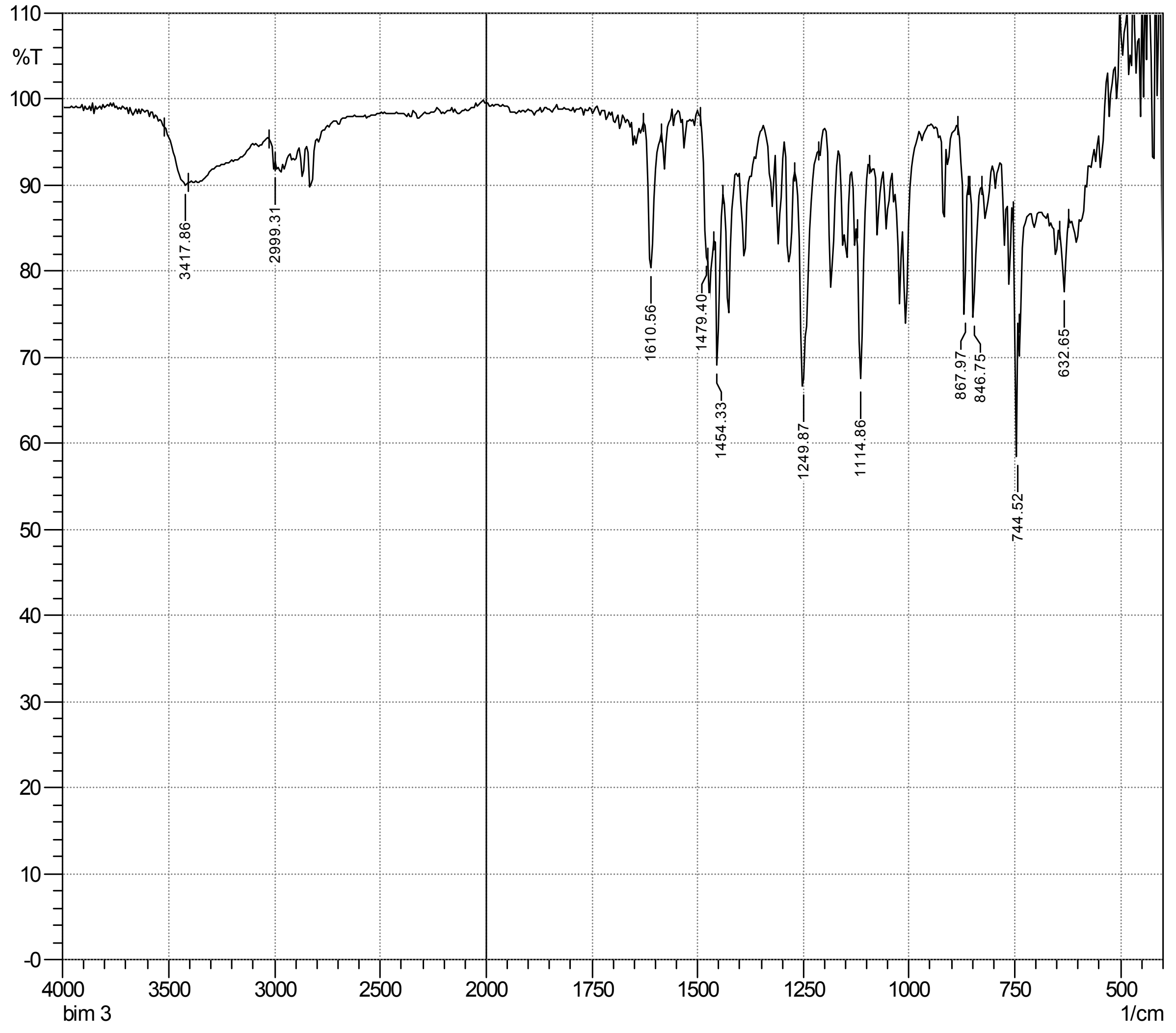

HRMS spectrum of *2-(4-Methoxyphenyl)-1-[2-(morpholin-4-yl)ethyl]-1H-benzimidazole* (**2b**)

Formula Predictor Report - Bim-3_11.lcd

Data File: C:\ LabSolutions\ Data\Analiz\Bim serisi\ Bim-3_11.lcd

Elmt	Val.	Min	Max	Elmt	Val.	Min	Max	Use Adduct
H	1	14	30	O	2	1	5	H
C	4	12	30	S	2	0	0	
N	3	3	4	Cl	1	0	0	

Error Margin (ppm):	10	DBE Range:	-2.0 - 1000.0	Electron Ions:	both
HC Ratio:	unlimited	Apply N Rule:	yes	Use MSn Info:	no
Max Isotopes:	3	Isotope RI (%):	1.00	Isotope Res:	10000
MSn Iso RI (%):	10.00	MSn Logic Mode:	AND	Max Results:	500

Event#: 1 MS(E+) Ret. Time : 4.467 -> 4.480 Scan# : 671 -> 673

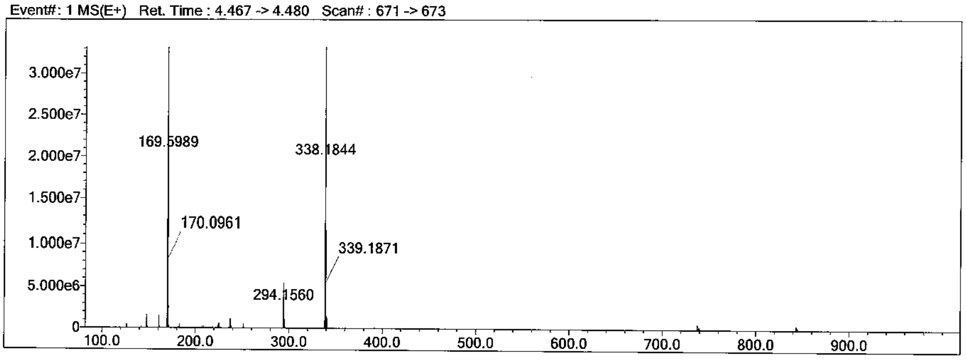

Measured region for 338.1844 m/z

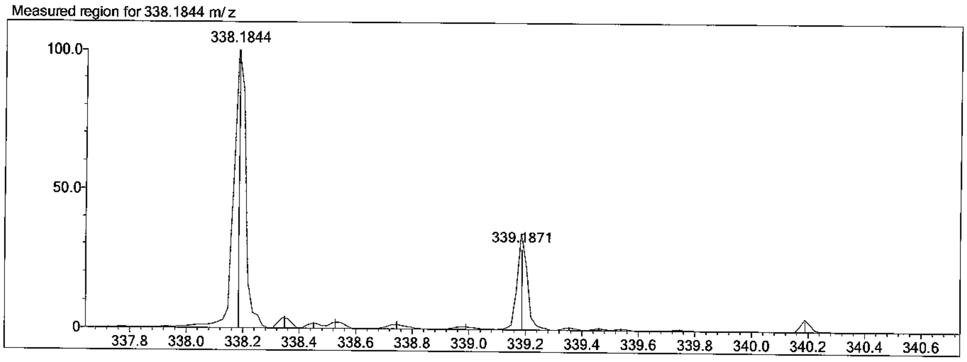

C20 H23 N3 O2 [M+H]+ : Predicted region for 338.1863 m/z

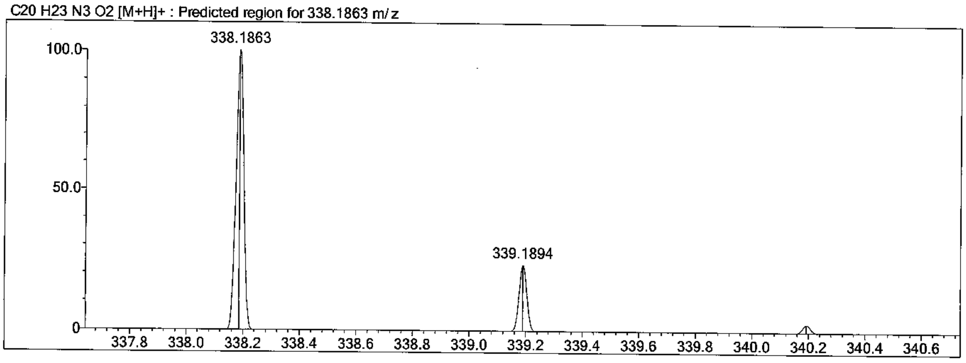

Rank	Score	Formula (M)	Ion	Meas. m/z	Pred. m/z	Df. (mDa)	Df. (ppm)	Iso	DBE
1	63.81	C20 H23 N3 O2	[M+H]+	338.1844	338.1863	-1.9	-5.62	76.15	11.0

^{13}C-NMR spectrum of *2-(4-Chlorophenyl)-1-[2-(morpholin-4-yl)ethyl]-1H-benzimidazole* (**2c**)

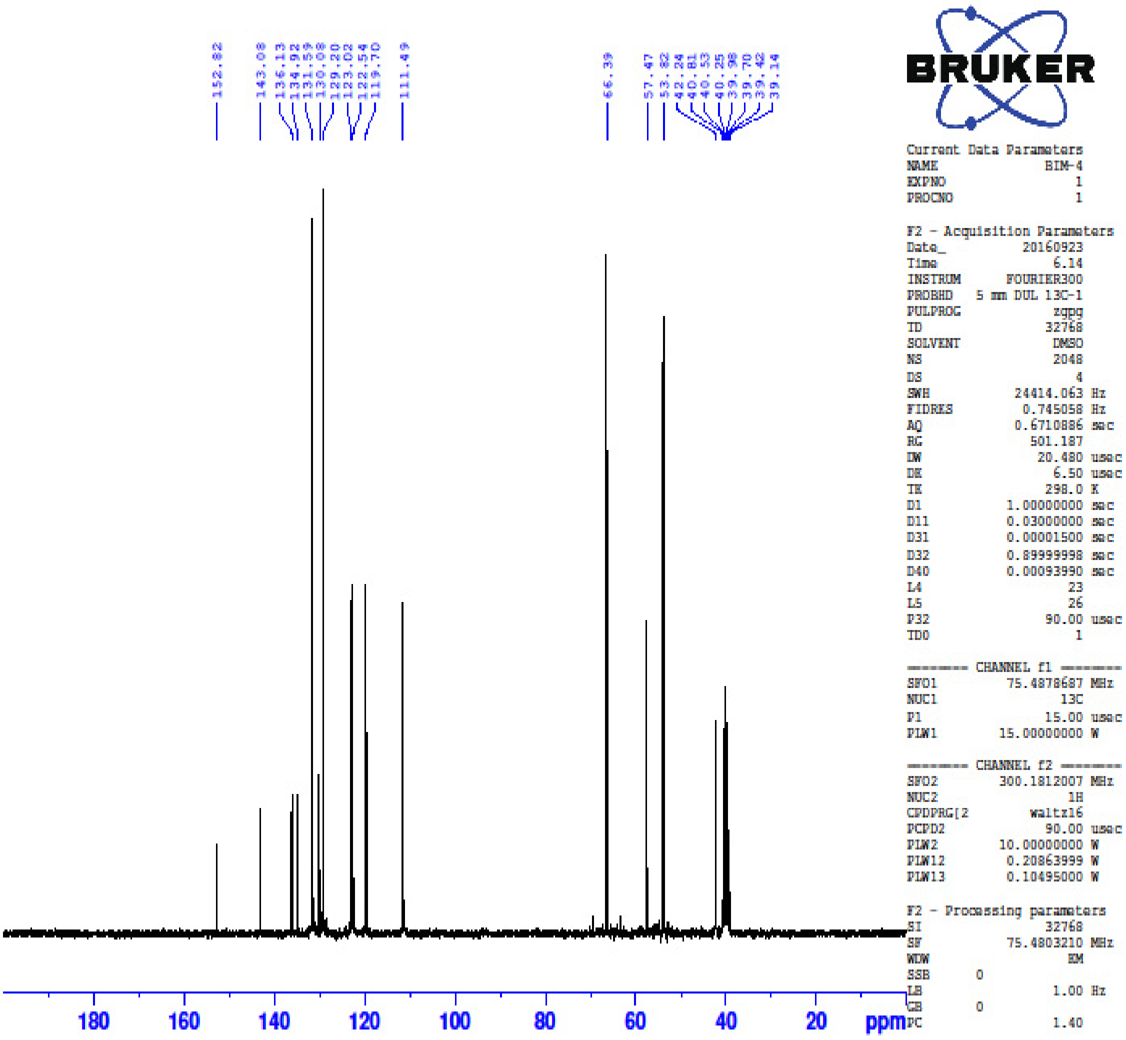

^{1}H-NMR spectrum of *2-(4-Chlorophenyl)-1-[2-(morpholin-4-yl)ethyl]-1H-benzimidazole* (***2c***)

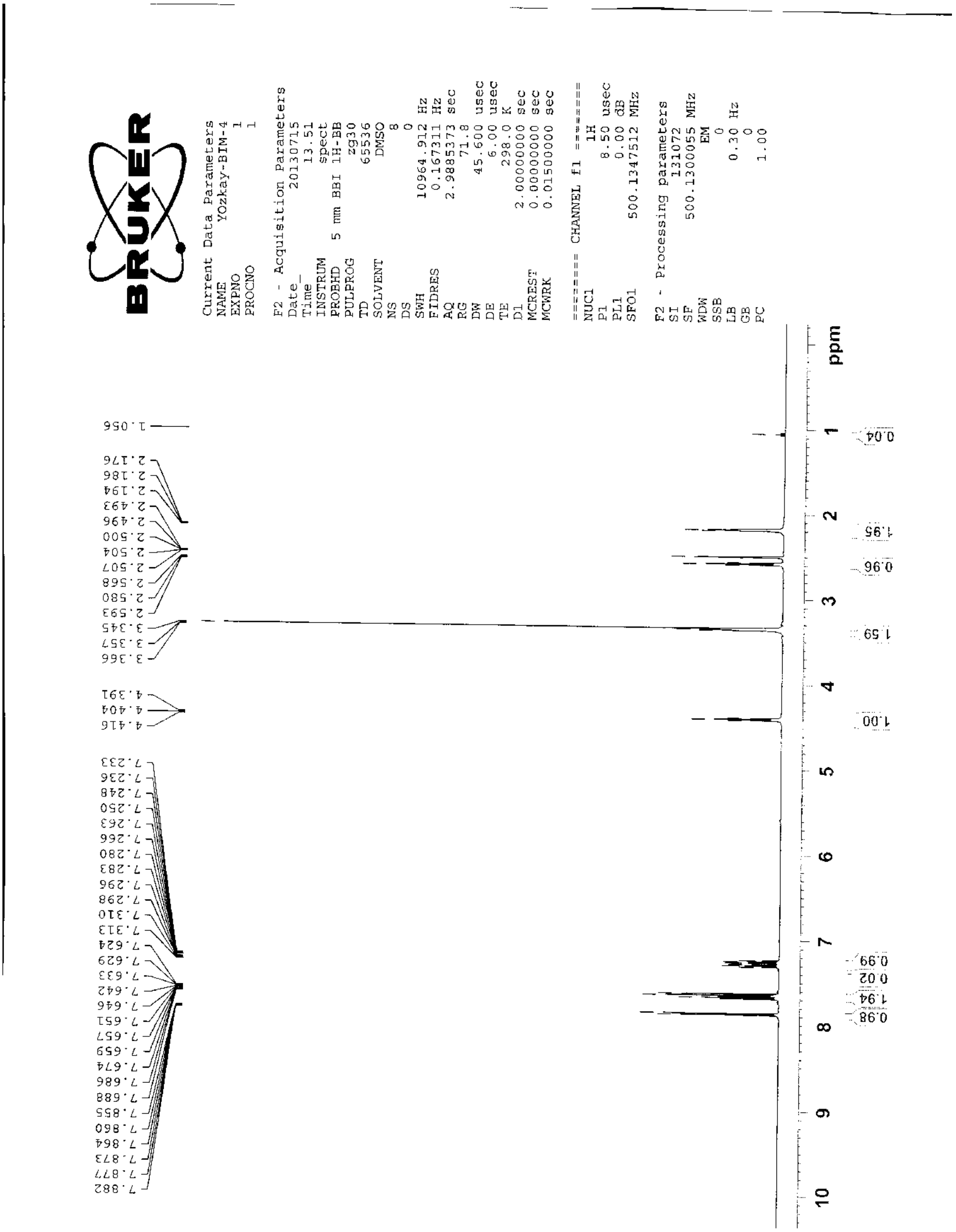

FTIR spectrum of *2-(4-Chlorophenyl)-1-[2-(morpholin-4-yl)ethyl]-1H-benzimidazole* (**2c**)

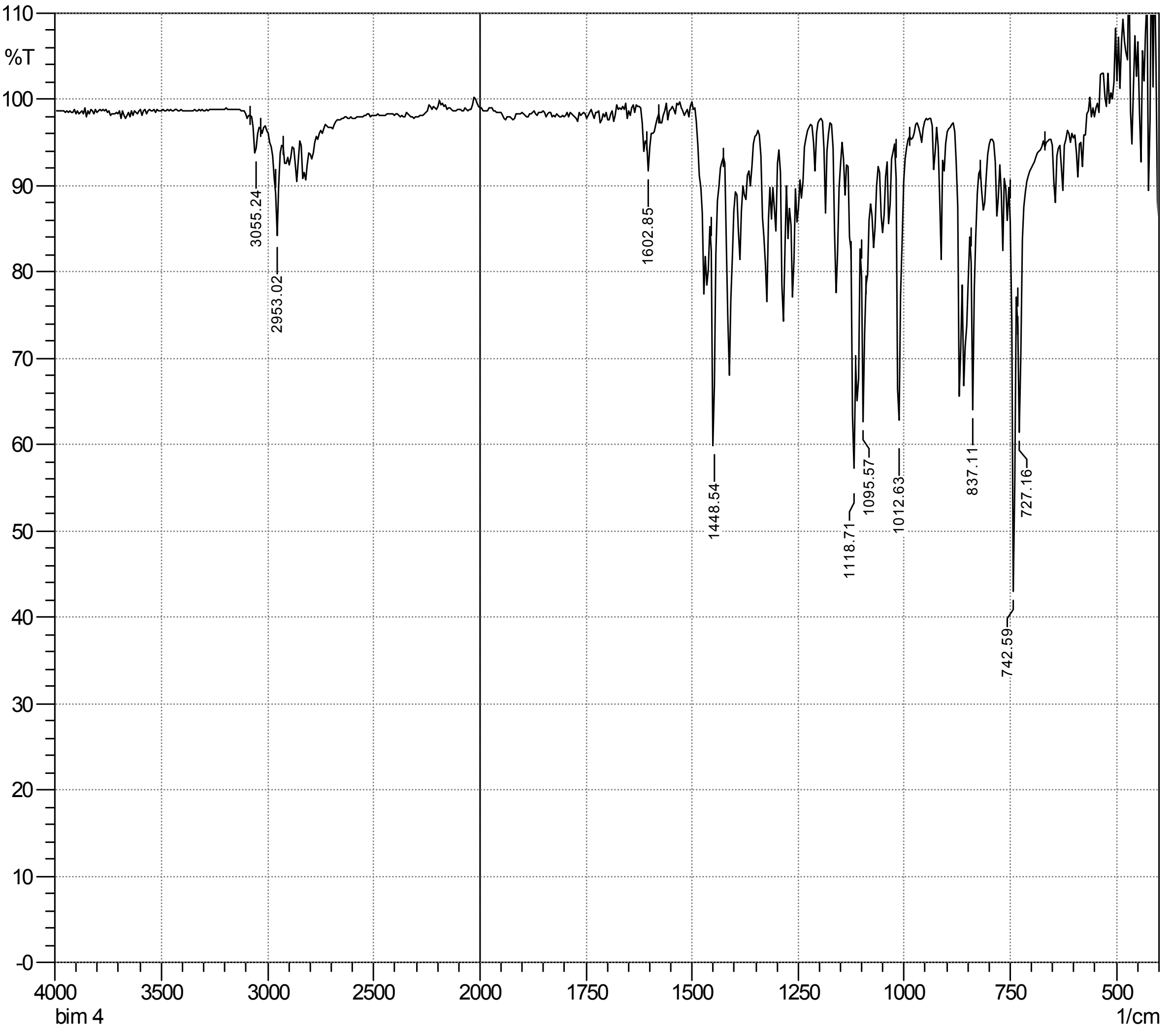

HRMS spectrum of *2-(4-Chlorophenyl)-1-[2-(morpholin-4-yl)ethyl]-1H-benzimidazole* (**2c**)

Data File: C:\ LabSolutions\ Data\Analiz\Bim serisi\ Bim-4_12.lcd

Elmt	Val.	Min	Max	Elmt	Val.	Min	Max	Use Adduct
H	1	14	30	O	2	1	5	H
C	4	12	30	S	2	0	0	
N	3	3	4	Cl	1	0	1	

Error Margin (ppm):	10	DBE Range:	-2.0 - 1000.0	Electron Ions:	both
HC Ratio:	unlimited	Apply N Rule:	yes	Use MSn Info:	no
Max Isotopes:	3	Isotope RI (%):	1.00	Isotope Res:	10000
MSn Iso RI (%):	10.00	MSn Logic Mode:	AND	Max Results:	500

Event#: 1 MS(E+) Ret. Time : 4.973 -> 5.120 Scan# : 747 -> 769

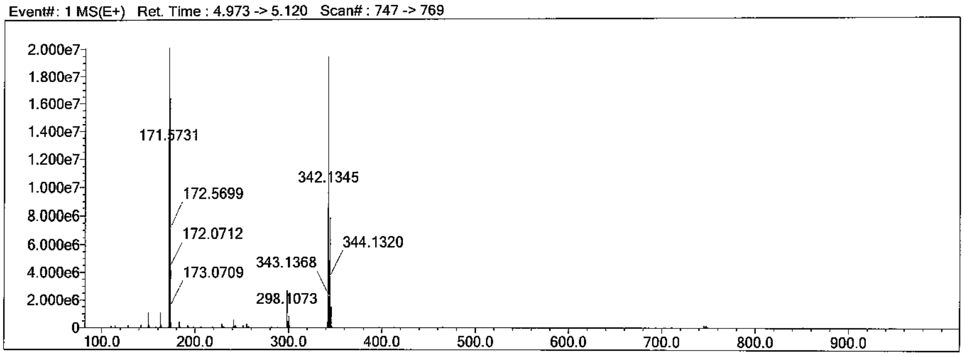

Measured region for 342.1345 m/z

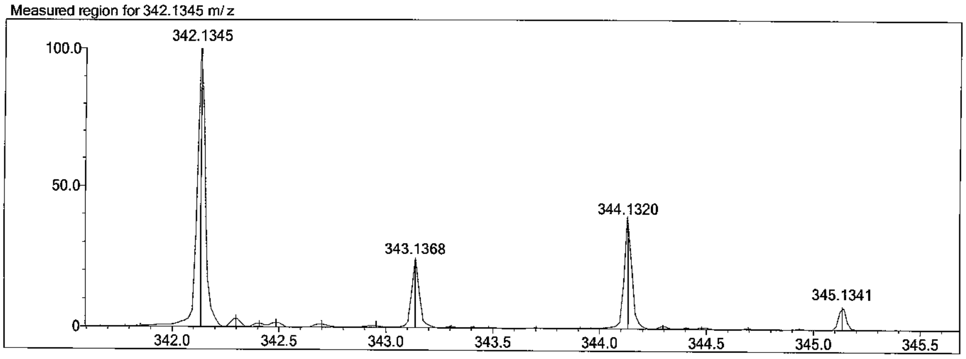

C19 H20 N3 O Cl [M+H]+ : Predicted region for 342.1368 m/z

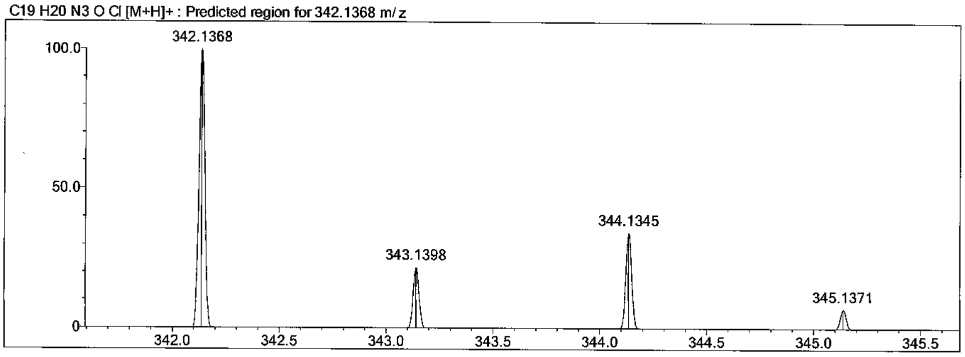

Rank	Score	Formula (M)	Ion	Meas. m/z	Pred. m/z	Df. (mDa)	Df. (ppm)	Iso	DBE
1	56.76	C19 H20 N3 O Cl	[M+H]+	342.1345	342.1368	-2.3	-6.72	77.97	11.0

^{13}C-NMR spectrum of *2-(4-Fluorophenyl)-1-[2-(morpholin-4-yl)ethyl]-1H-benzimidazole* (**2d**)

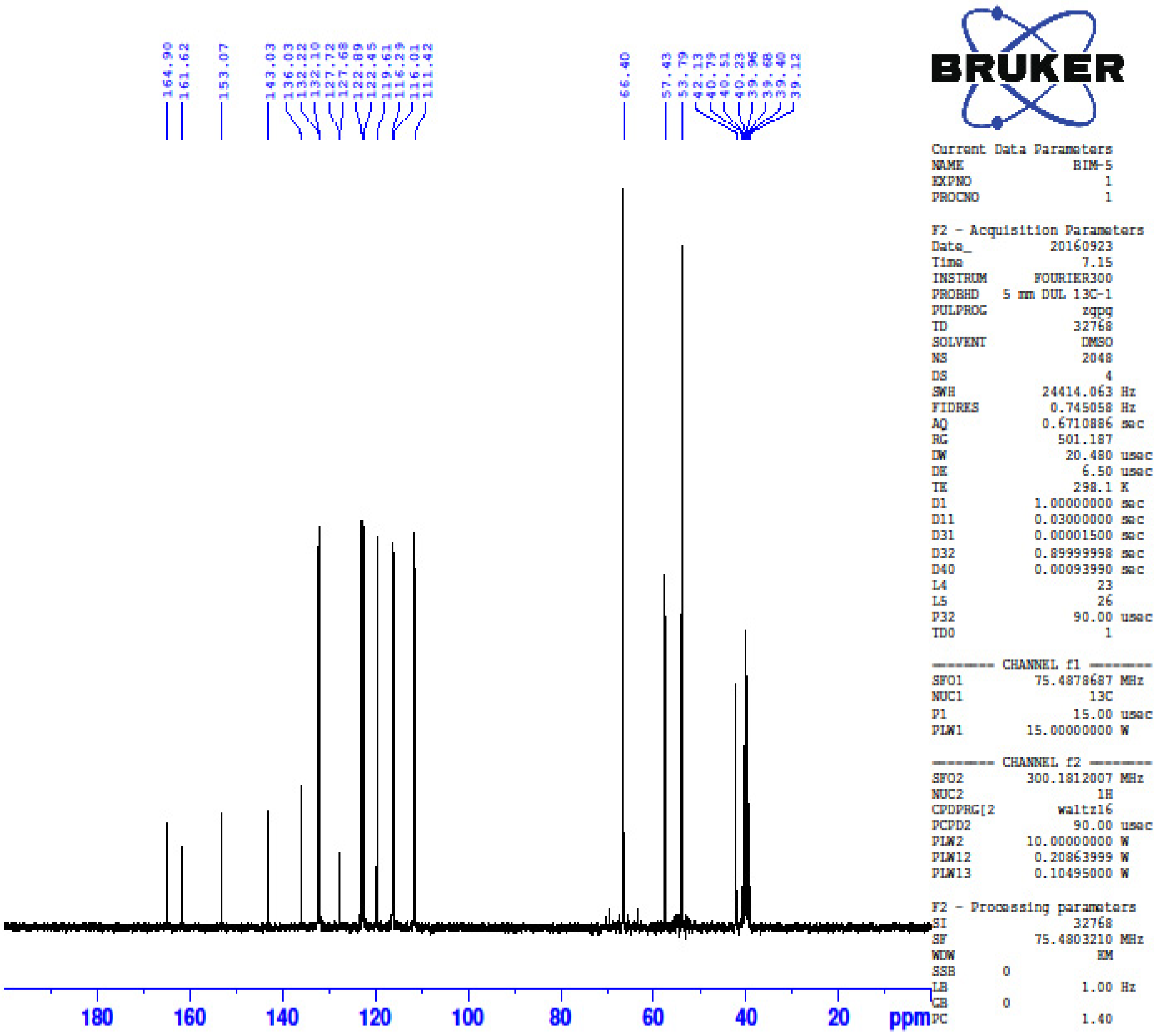

^{1}H-NMR spectrum of *2-(4-Fluorophenyl)-1-[2-(morpholin-4-yl)ethyl]-1H-benzimidazole* ***(2d)***

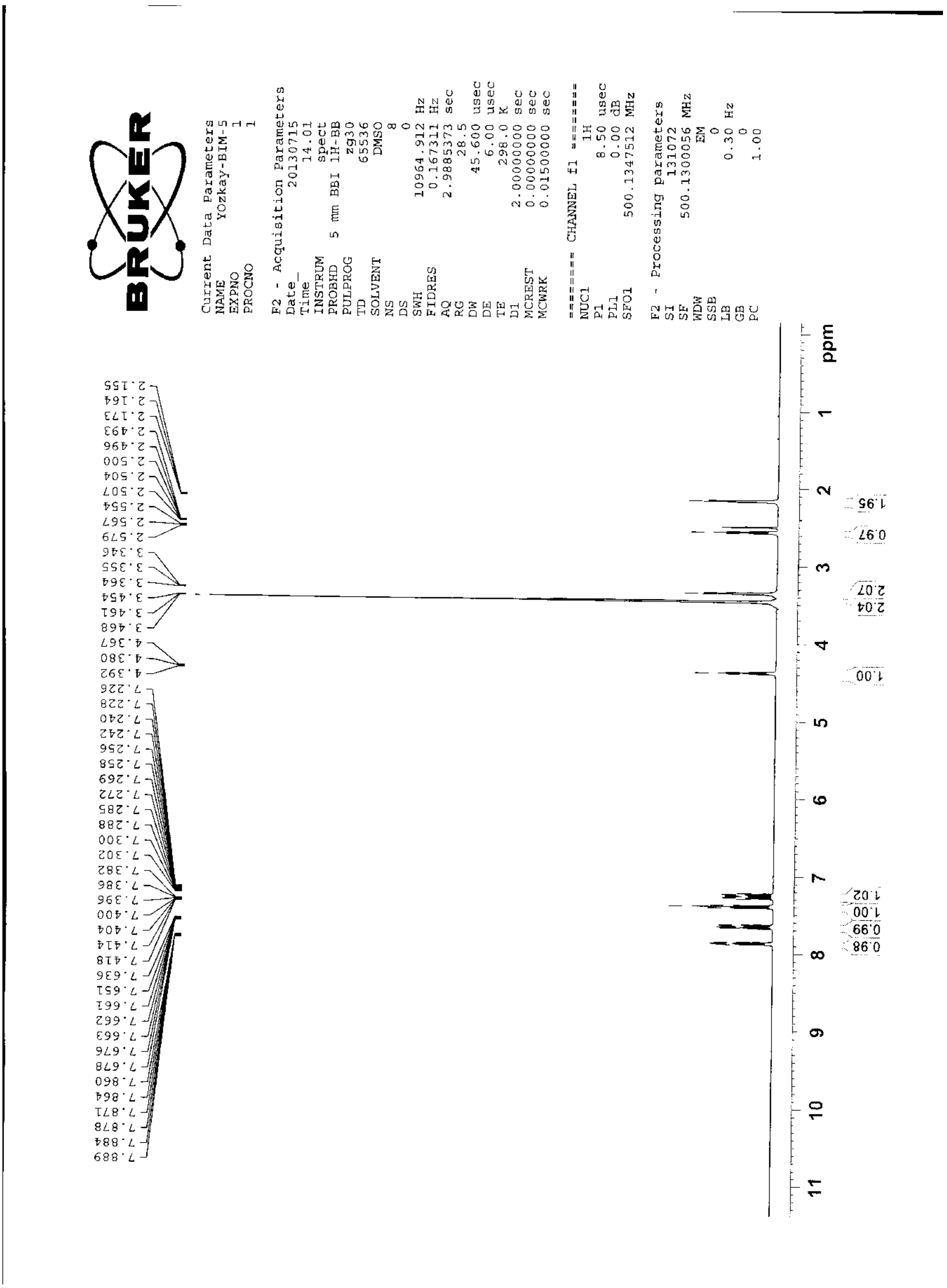

FTIR spectrum of *2-(4-Fluorophenyl)-1-[2-(morpholin-4-yl)ethyl]-1H-benzimidazole* (***2d***)

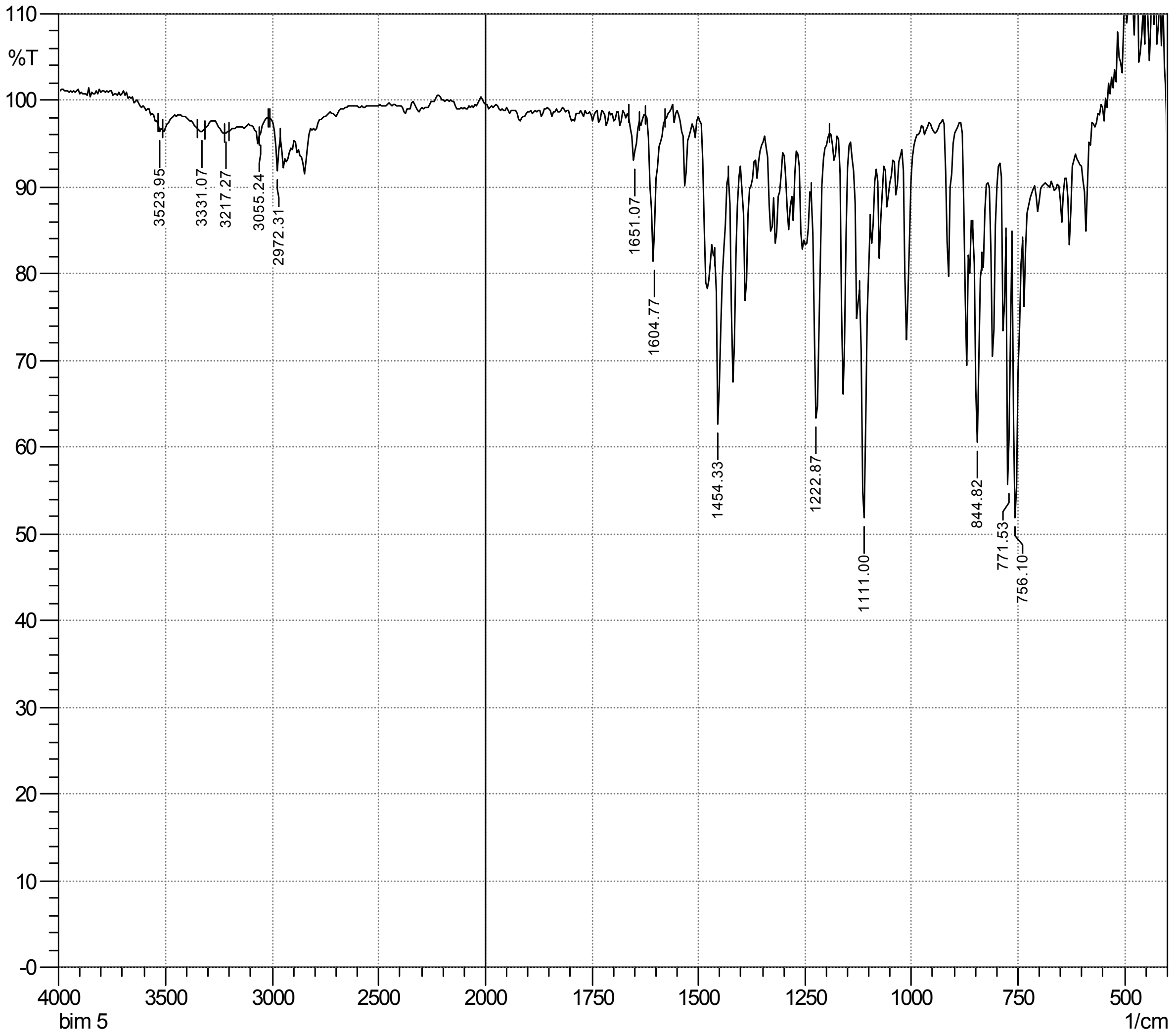

HRMS spectrum of *2-(4-Fluorophenyl)-1-[2-(morpholin-4-yl)ethyl]-1H-benzimidazole* (***2d***)

Formula Predictor Report - Bim-5_13.lcd

Data File: C:\ LabSolutions\ Data\Analiz\Bim serisi\ Bim-5_13.lcd

Elmt	Val.	Min	Max	Elmt	Val.	Min	Max	Elmt	Val.	Min	Max	Use Adduct
H	1	14	30	O	2	1	5	Cl	1	0	0	H
C	4	12	30	F	1	0	1					
N	3	3	4	S	2	0	0					

Error Margin (ppm):	10	DBE Range:	-2.0 - 1000.0	Electron Ions:	both
HC Ratio:	unlimited	Apply N Rule:	yes	Use MSn Info:	no
Max Isotopes:	3	Isotope RI (%):	1.00	Isotope Res:	10000
MSn Iso RI (%):	10.00	MSn Logic Mode:	AND	Max Results:	500

Event#: 1 MS(E+) Ret. Time : 4.587 -> 4.600 Scan# : 689 -> 691

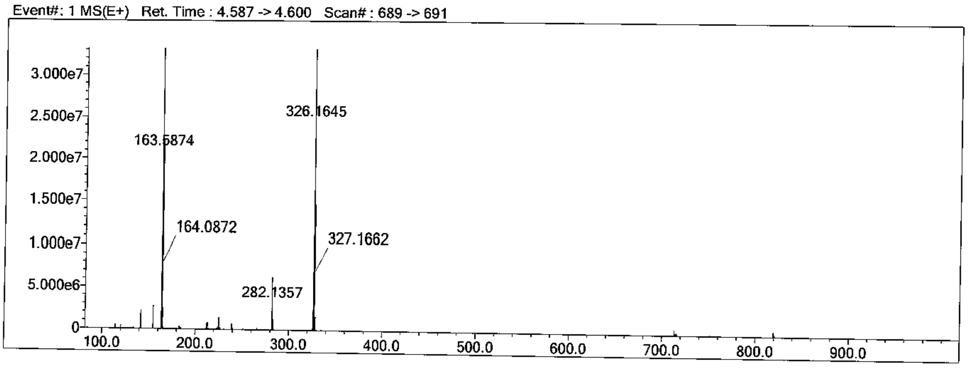

Measured region for 326.1645 m/z

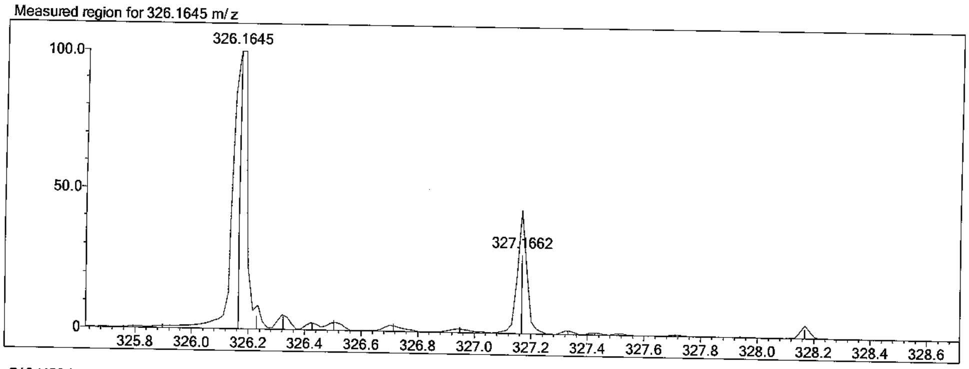

C19 H20 N3 O F [M+H]+ : Predicted region for 326.1663 m/z

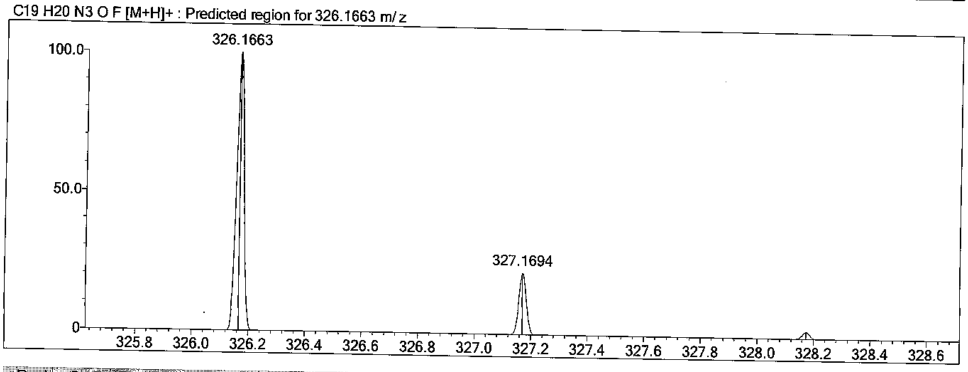

Rank	Score	Formula (M)	Ion	Meas. m/z	Pred. m/z	Df. (mDa)	Df. (ppm)	Iso	DBE
1	51.00	C19 H20 N3 O F	[M+H]+	326.1645	326.1663	-1.8	-5.52	60.14	11.0

^{13}C-NMR spectrum of *2-(4-Isopropylphenyl)-1-[2-(morpholin-4-yl)ethyl]-1H-benzimidazole* (***2e***)

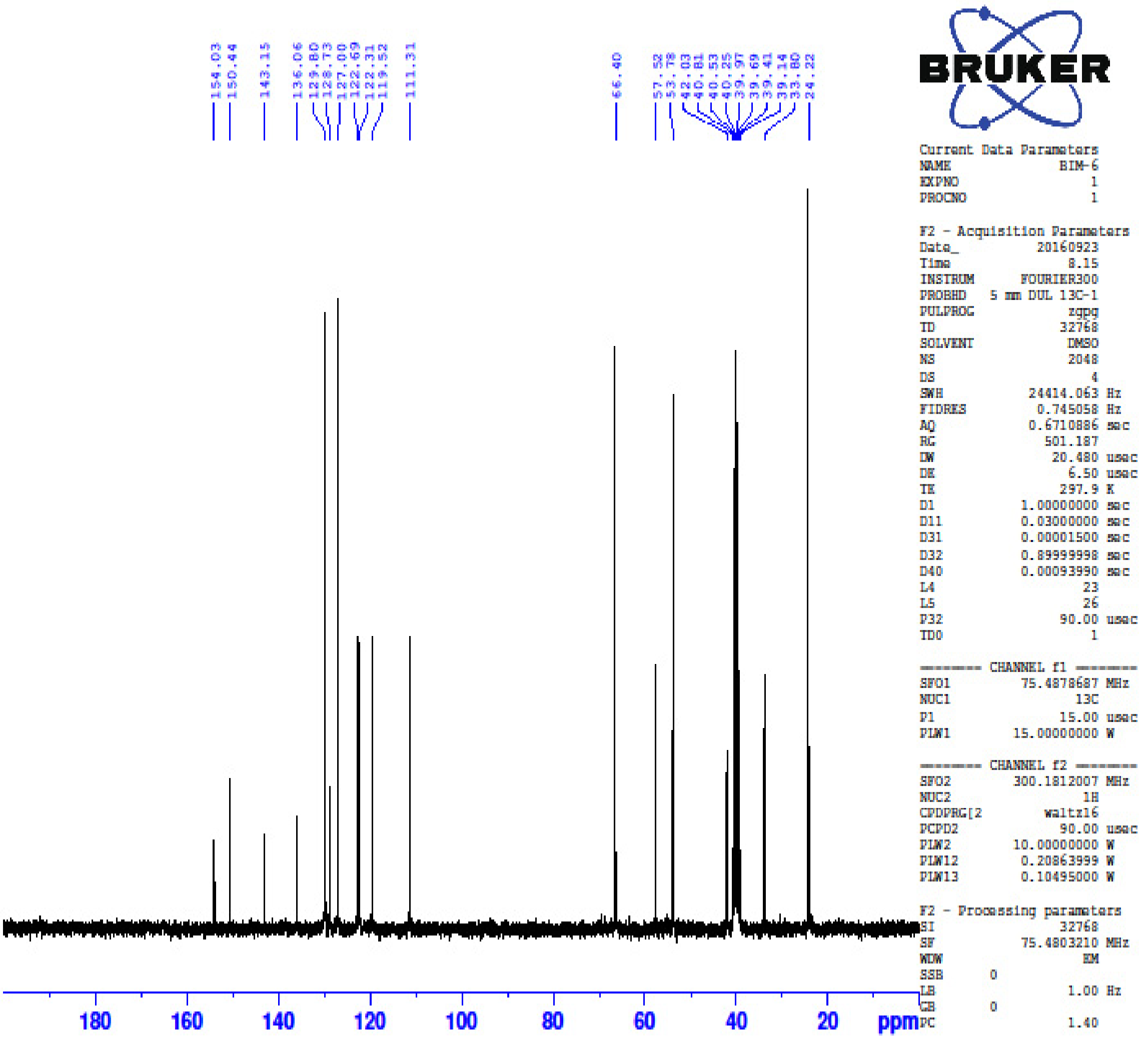

^{1}H-NMR spectrum of *2-(4-Isopropylphenyl)-1-[2-(morpholin-4-yl)ethyl]-1H-benzimidazole* (***2e***)

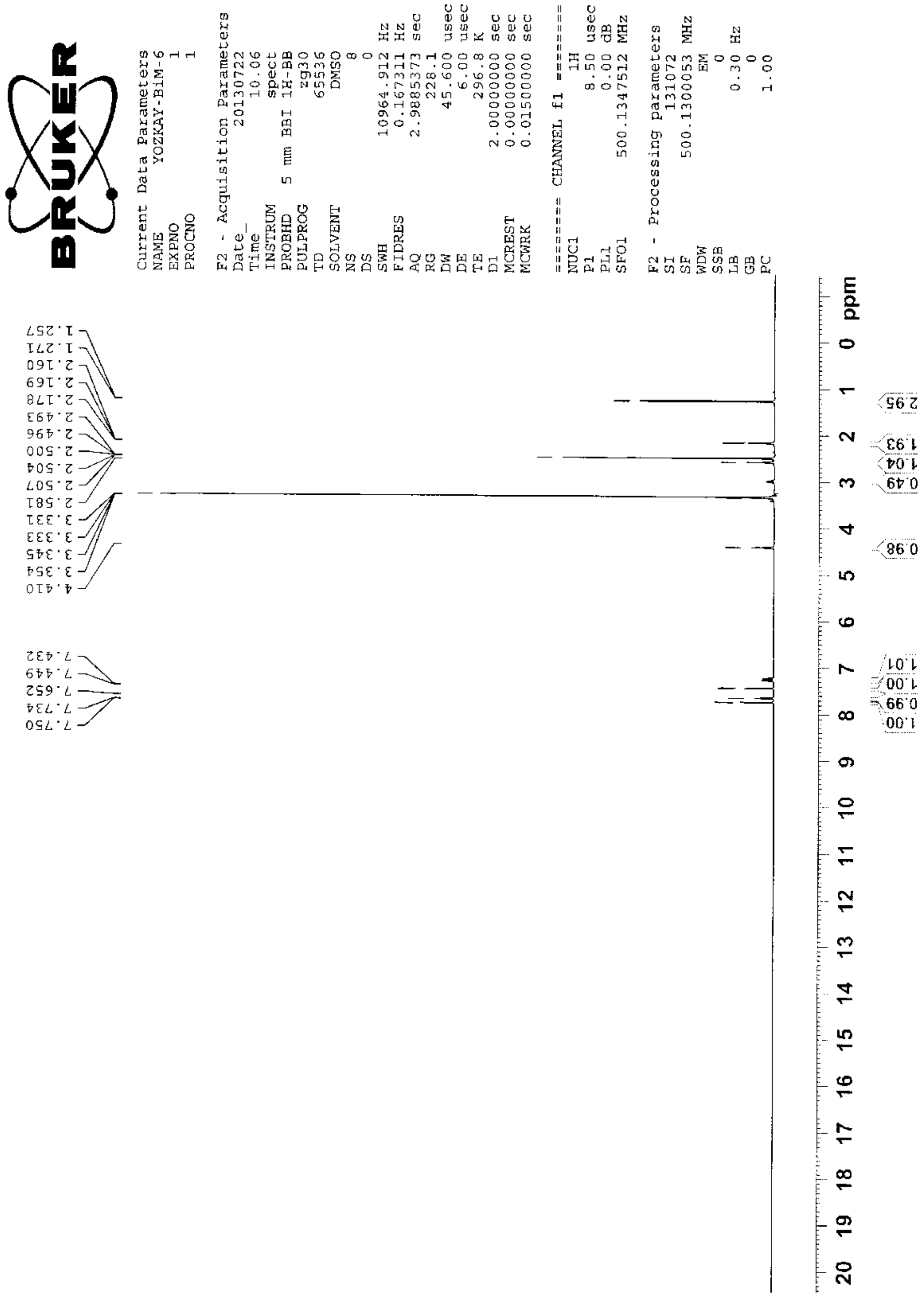

FTIR spectrum of *2-(4-Isopropylphenyl)-1-[2-(morpholin-4-yl)ethyl]-1H-benzimidazole* ***(2e)***

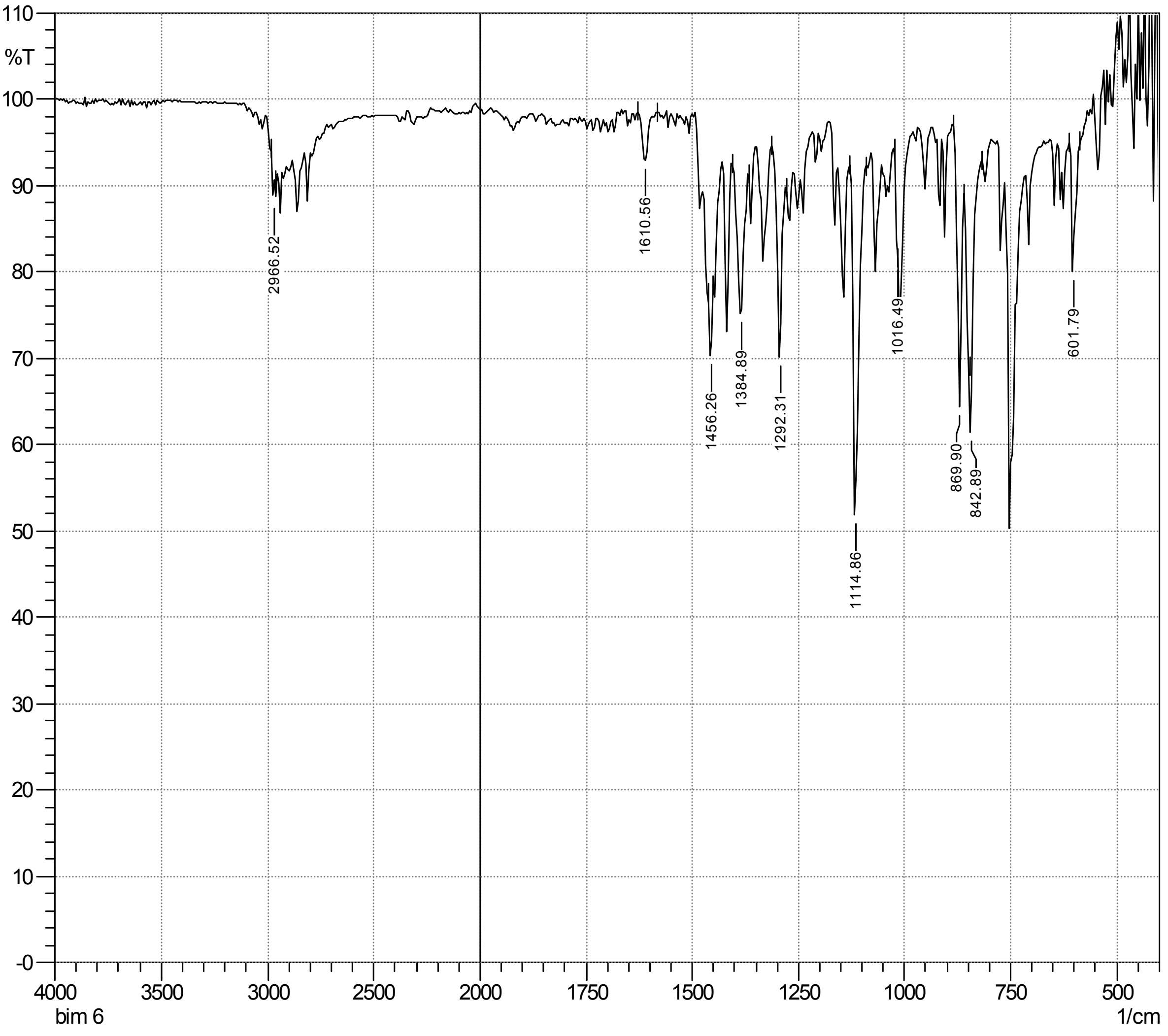

HRMS spectrum of *2-(4-Isopropylphenyl)-1-[2-(morpholin-4-yl)ethyl]-1H-benzimidazole* (***2e***)

Formula Predictor Report - Bim-6_14.lcd

Data File: C:\LabSolutions\ Data\Analiz\Bim serisi\ Bim-6_14.lcd

Elmt	Val.	Min	Max	Elmt	Val.	Min	Max	Elmt	Val.	Min	Max	Use Adduct
H	1	14	30	O	2	1	5	Cl	1	0	0	H
C	4	12	30	F	1	0	1					
N	3	3	4	S	2	0	0					

Error Margin (ppm): 10
HC Ratio: unlimited
Max Isotopes: 3
MSn Iso RI (%): 10.00

DBE Range: -2.0 - 1000.0
Apply N Rule: yes
Isotope RI (%): 1.00
MSn Logic Mode: AND

Electron Ions: both
Use MSn Info: no
Isotope Res: 10000
Max Results: 500

Event#: 1 MS(E+) Ret. Time : 5.280 -> 5.307 Scan# : 793 -> 797

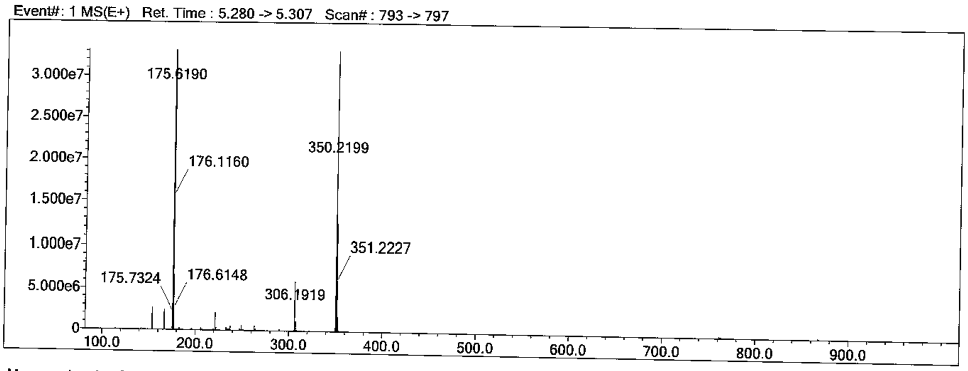

Measured region for 350.2199 m/z

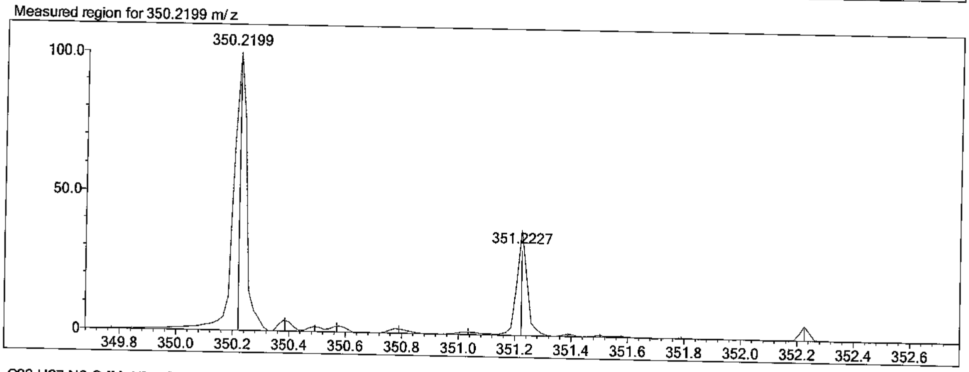

C22 H27 N3 O [M+H]+ : Predicted region for 350.2227 m/z

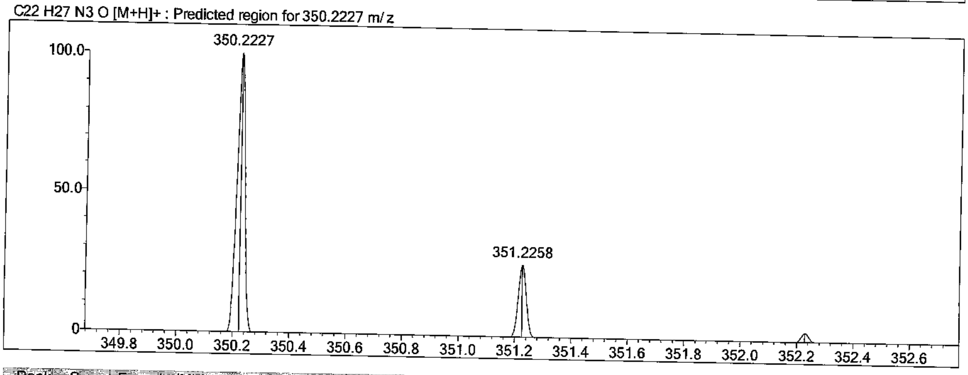

Rank	Score	Formula (M)	Ion	Meas. m/z	Pred. m/z	Df. (mDa)	Df. (ppm)	Iso	DBE
1	46.38	C22 H27 N3 O	[M+H]+	350.2199	350.2227	-2.8	-7.99	77.17	11.0

^{13}C-NMR spectrum of *2-(4-Benzyloxyphenyl)-1-[2-(morpholin-4-yl)ethyl]-1H-benzimidazole* (***2f***)

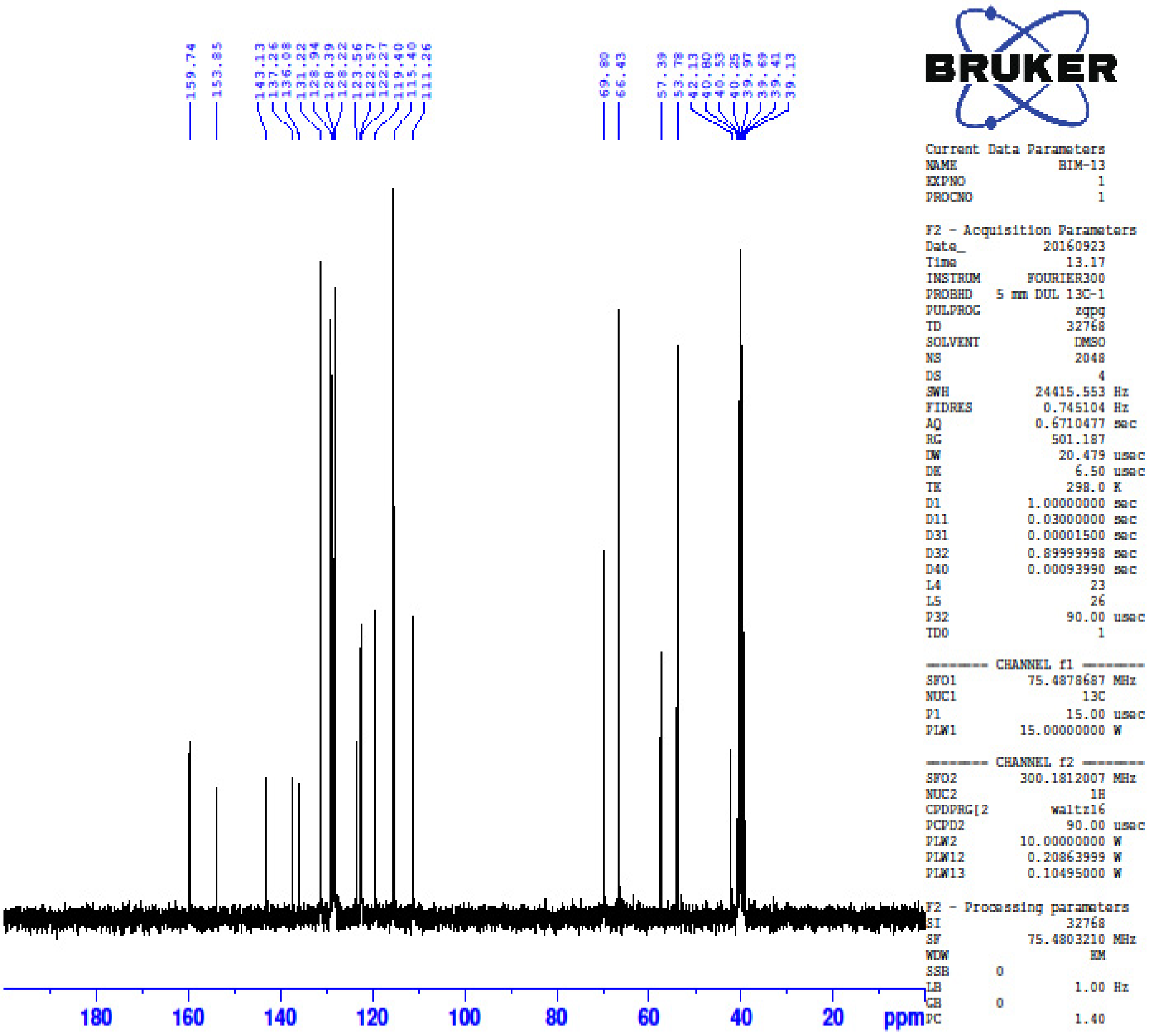

^{1}H-NMR spectrum of *2-(4-Benzyloxyphenyl)-1-[2-(morpholin-4-yl)ethyl]-1H-benzimidazole* (***2f***)

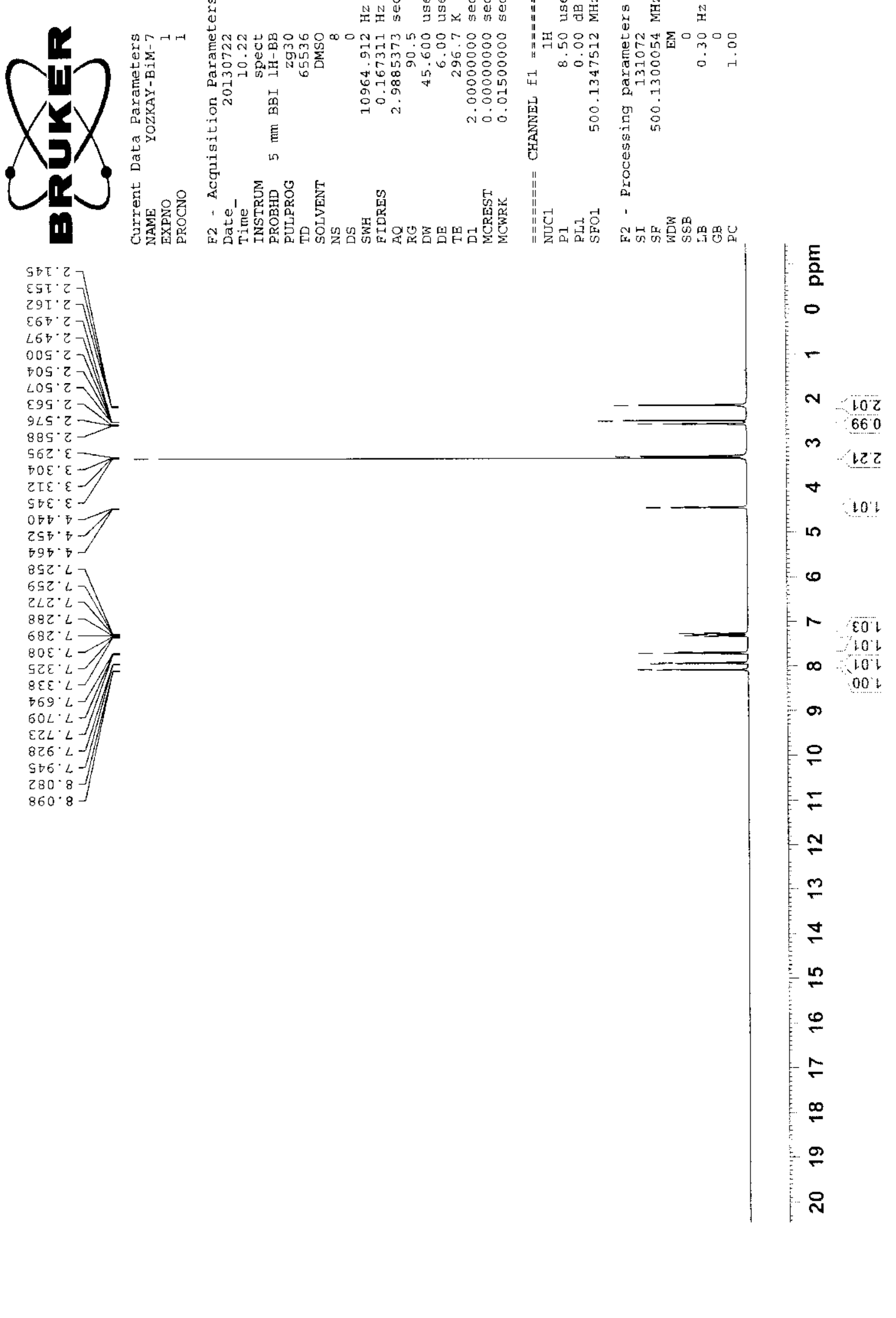

FTIR spectrum of *2-(4-Benzyloxyphenyl)-1-[2-(morpholin-4-yl)ethyl]-1H-benzimidazole* (***2f***)

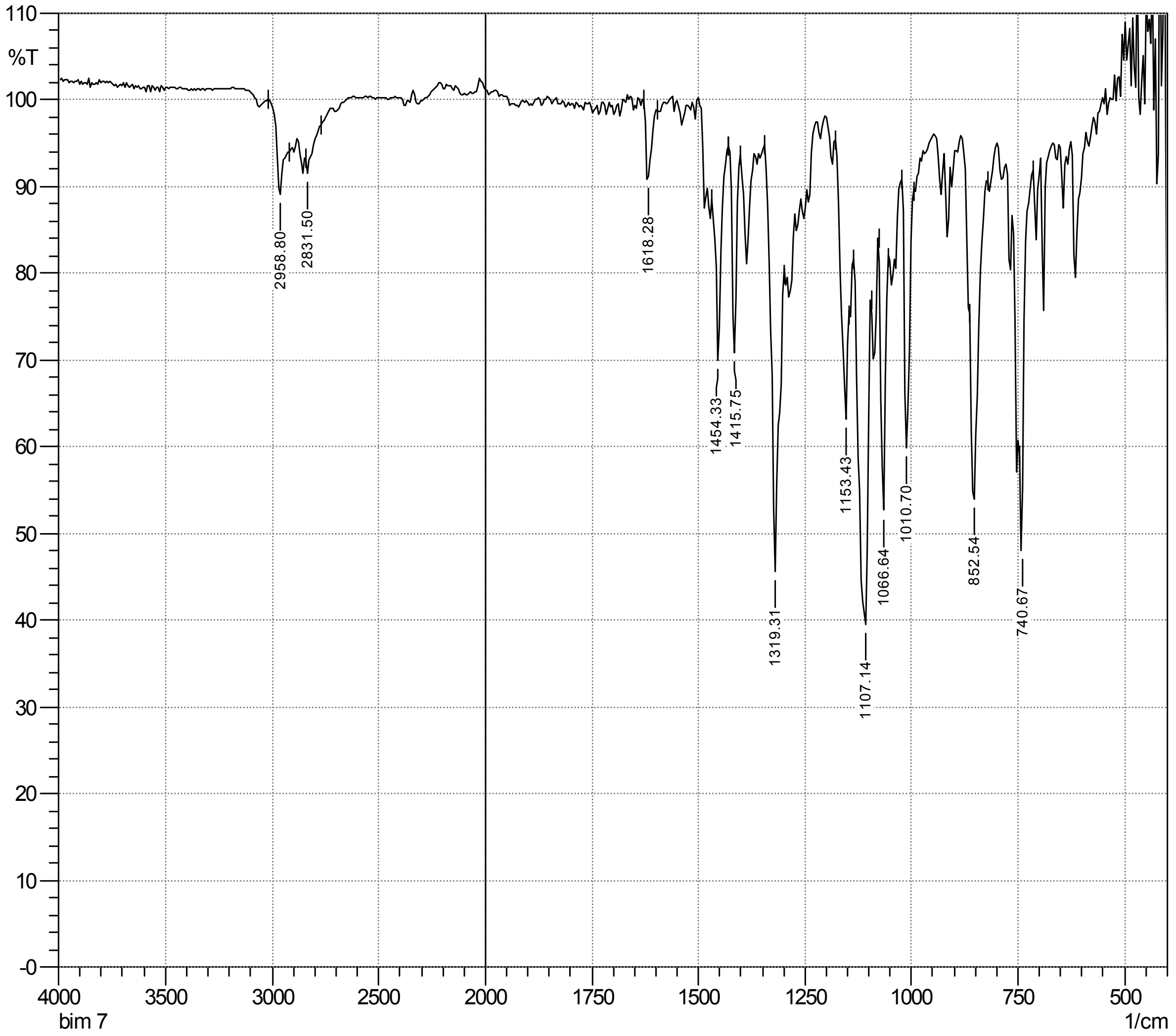

HRMS spectrum of *2-(4-Benzyloxyphenyl)-1-[2-(morpholin-4-yl)ethyl]-1H-benzimidazole (**2f**)*

Data File: C:\ LabSolutions\ Data\Analiz\Bim serisi\ Bim-7_15.lcd

Elmt	Val.	Min	Max	Elmt	Val.	Min	Max	Elmt	Val.	Min	Max	Use Adduct
H	1	14	30	O	2	1	5	Cl	1	0	0	H
C	4	12	30	F	1	0	3					
N	3	3	4	S	2	0	0					

Error Margin (ppm):	10	DBE Range:	-2.0 - 1000.0	Electron Ions:	both
HC Ratio:	unlimited	Apply N Rule:	yes	Use MSn Info:	no
Max Isotopes:	3	Isotope RI (%):	1.00	Isotope Res:	10000
MSn Iso RI (%):	10.00	MSn Logic Mode:	AND	Max Results:	500

Event#: 1 MS(E+) Ret. Time : 5.453 -> 5.493 Scan# : 819 -> 825

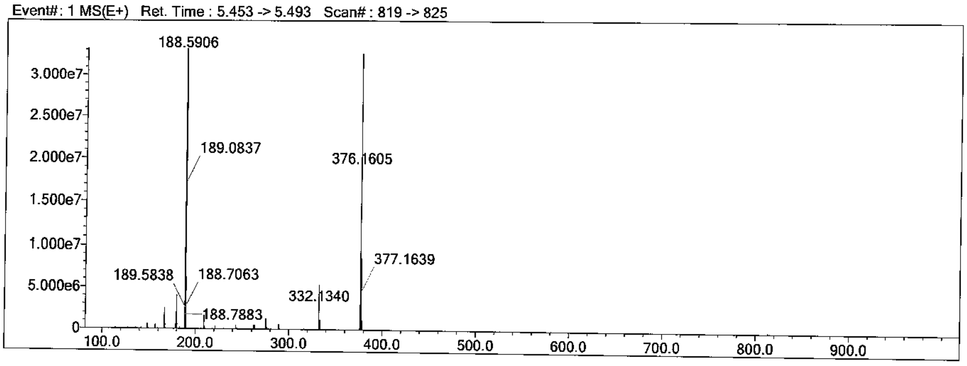

Measured region for 376.1605 m/z

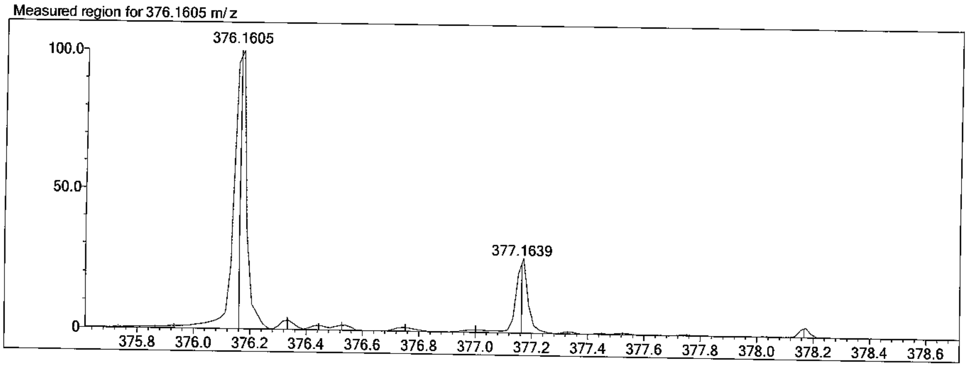

C20 H20 N3 O F3 [M+H]+ : Predicted region for 376.1631 m/z

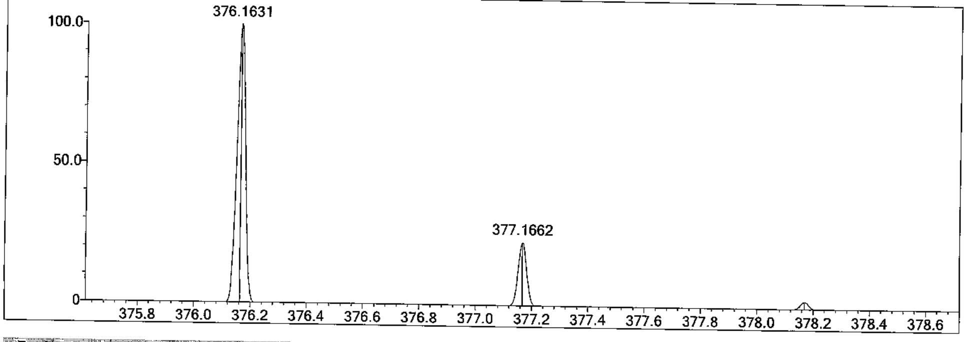

Rank	Score	Formula (M)	Ion	Meas. m/z	Pred. m/z	Df. (mDa)	Df. (ppm)	Iso	DBE
1	70.90	C20 H20 N3 O F3	[M+H]+	376.1605	376.1631	-2.6	-6.91	100.00	11.0

^{13}C-NMR spectrum of *2-(4-Bromophenyl)-1-[2-(morpholin-4-yl)ethyl]-1H-benzimidazole* (**2g**)

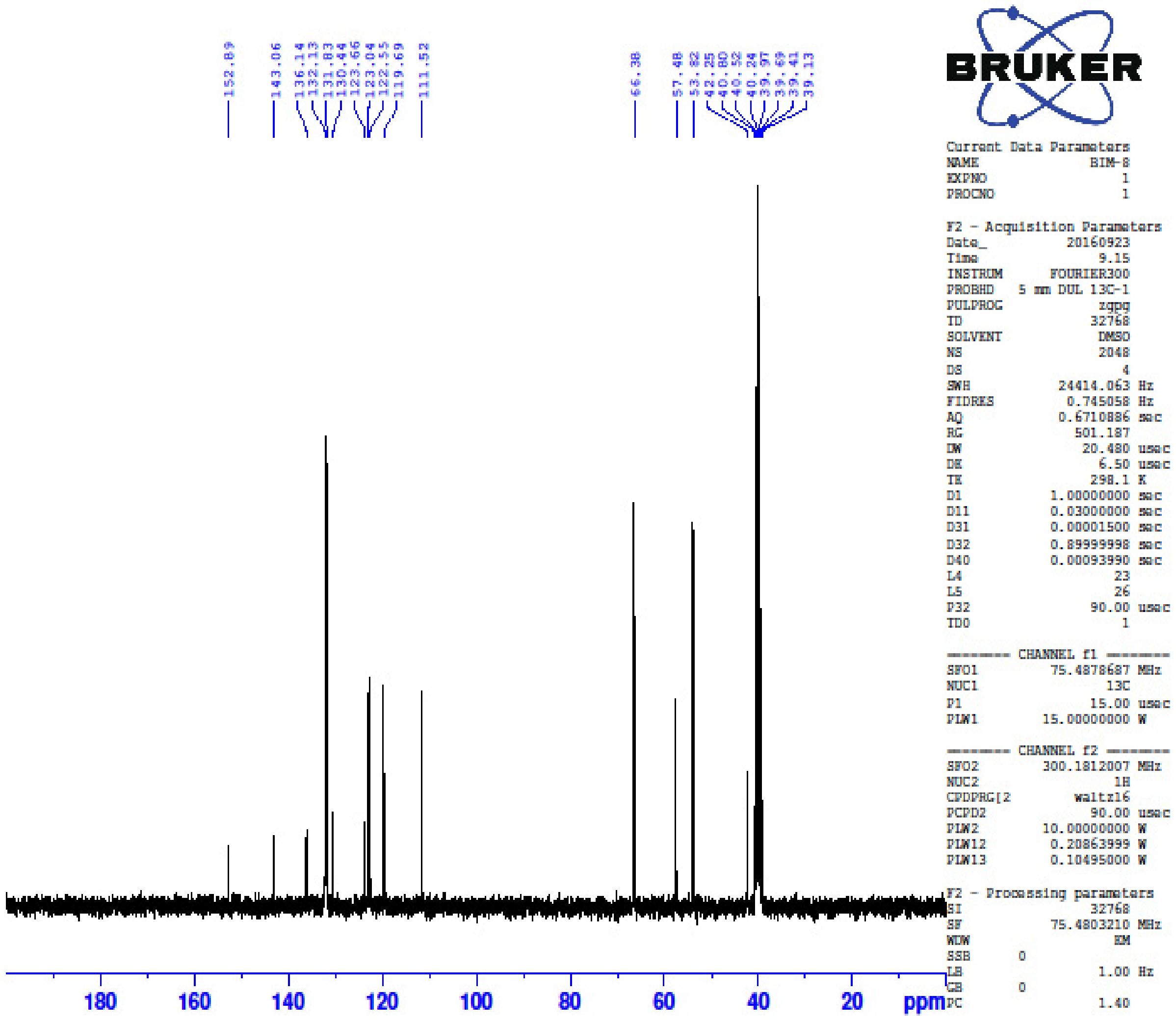

^{1}H-NMR spectrum of *2-(4-Bromophenyl)-1-[2-(morpholin-4-yl)ethyl]-1H-benzimidazole* (***2g)***

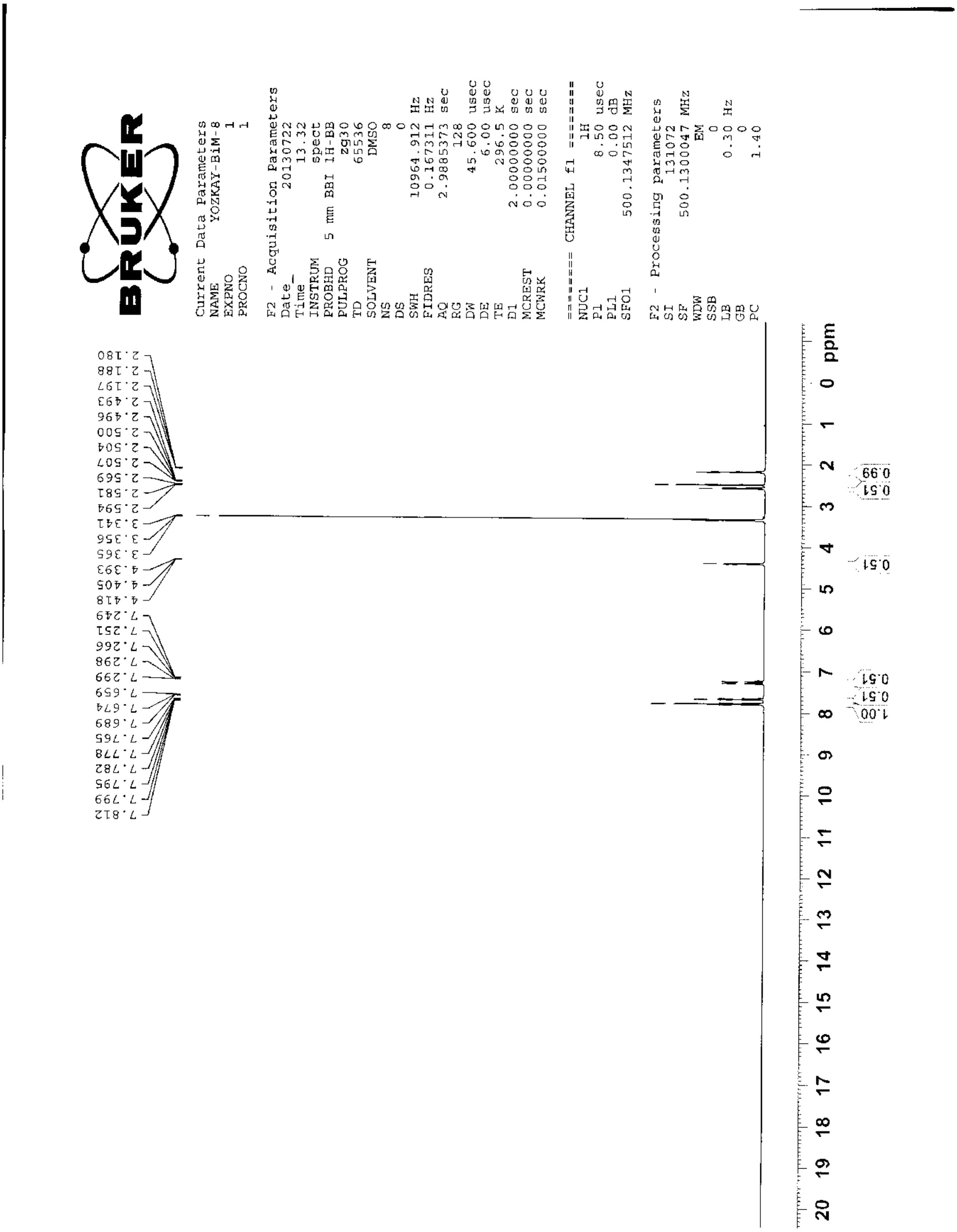

FTIR spectrum of *2-(4-Bromophenyl)-1-[2-(morpholin-4-yl)ethyl]-1H-benzimidazole* (**2g**)

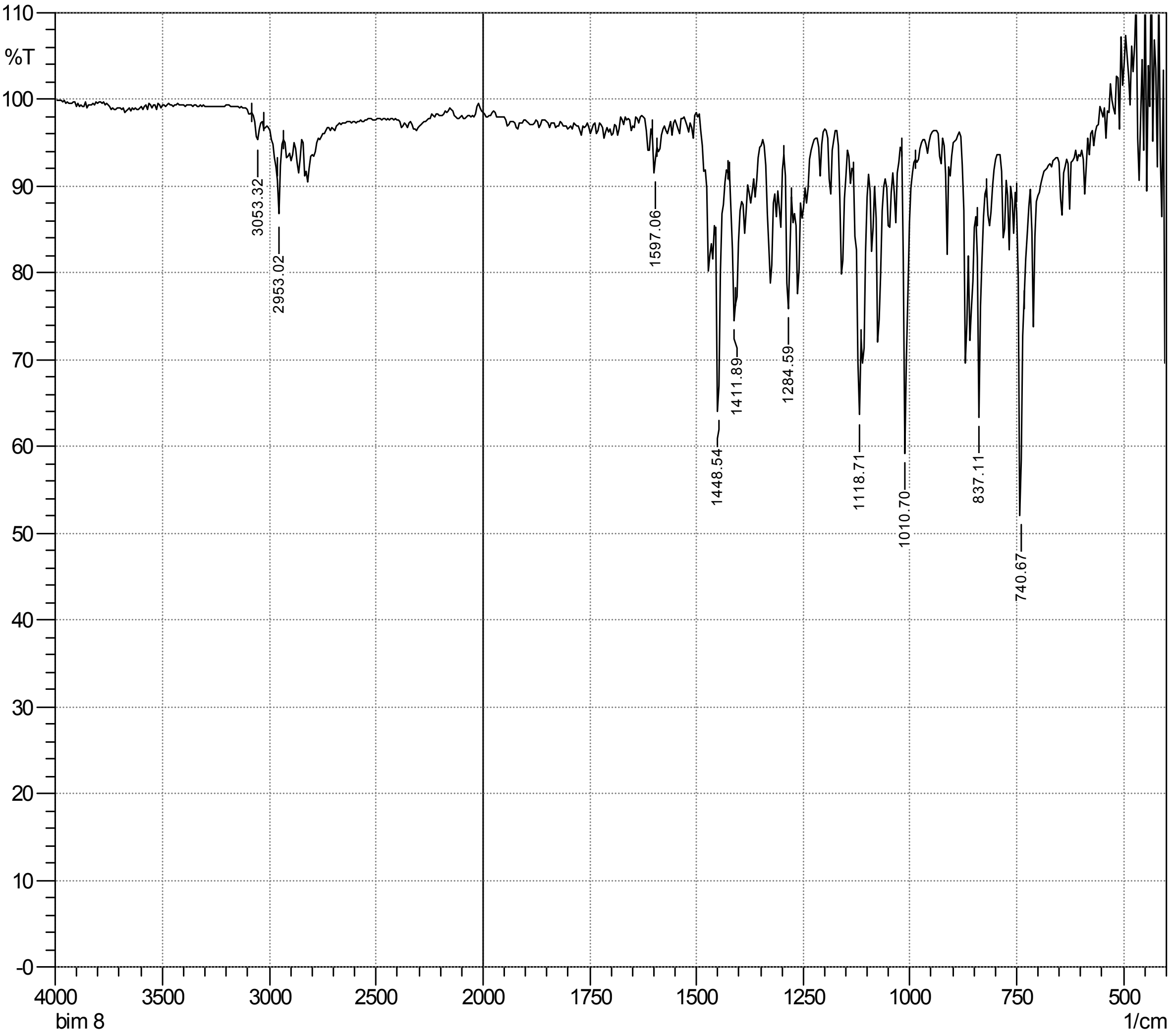

HRMS spectrum of *2-(4-Bromophenyl)-1-[2-(morpholin-4-yl)ethyl]-1H-benzimidazole* (***2g***)

Data File: C:\ LabSolutions\ Data\Analiz\Bim serisi\ Bim-8_16.lcd

Elmt	Val.	Min	Max	Elmt	Val.	Min	Max	Elmt	Val.	Min	Max	Use Adduct
H	1	14	30	O	2	1	5	Cl	1	0	0	H
C	4	12	30	F	1	0	3	Br	1	0	1	
N	3	3	4	S	2	0	0					

Error Margin (ppm):	10	DBE Range:	-2.0 - 1000.0	Electron Ions:	both
HC Ratio:	unlimited	Apply N Rule:	yes	Use MSn Info:	no
Max Isotopes:	3	Isotope RI (%):	1.00	Isotope Res:	10000
MSn Iso RI (%):	10.00	MSn Logic Mode:	AND	Max Results:	500

Event#: 1 MS(E+) Ret. Time : 4.987 -> 5.027 Scan# : 749 -> 755

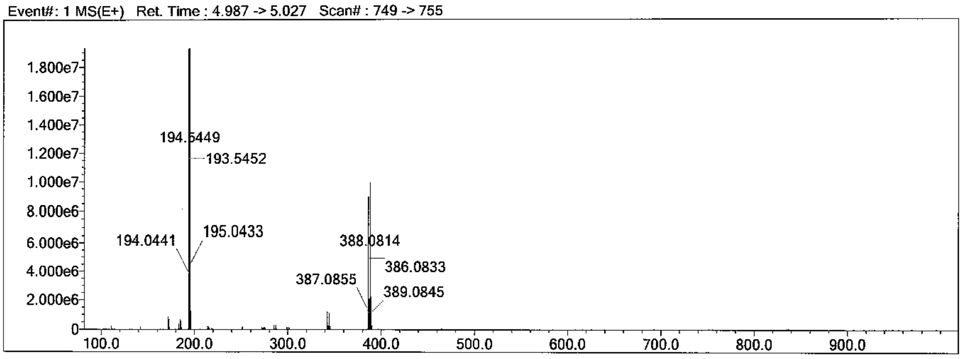

Measured region for 386.0833 m/z

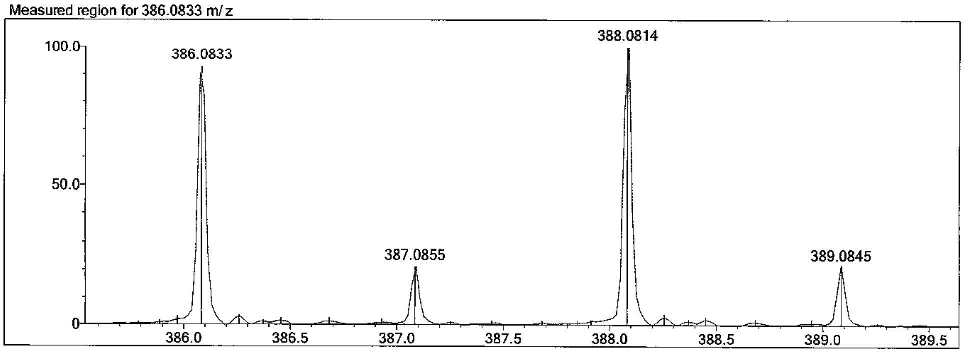

C19 H20 N3 O Br [M+H]+ : Predicted region for 386.0862 m/z

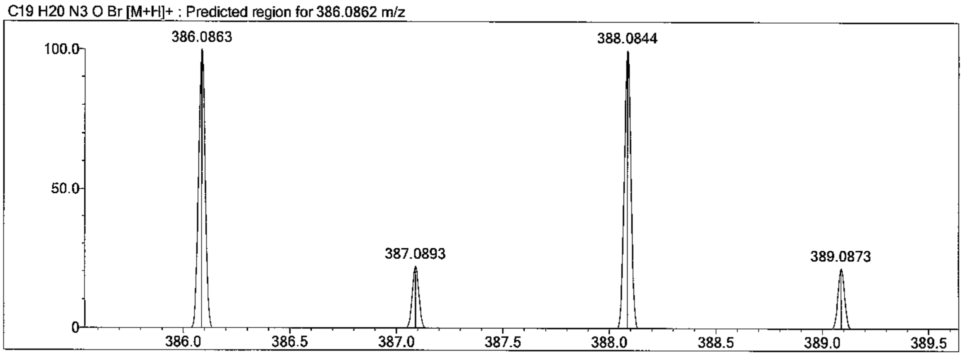

Rank	Score	Formula (M)	Ion	Meas. m/z	Pred. m/z	Df. (mDa)	Df. (ppm)	Iso	DBE
1	45.47	C19 H20 N3 O Br	[M+H]+	386.0833	386.0862	-2.9	-7.51	70.06	11.0

^{13}C-NMR spectrum of *2-(4-Diethylaminophenyl)-1-[2-(morpholin-4-yl) ethyl]-1H-benzimidazole* (***2h***)

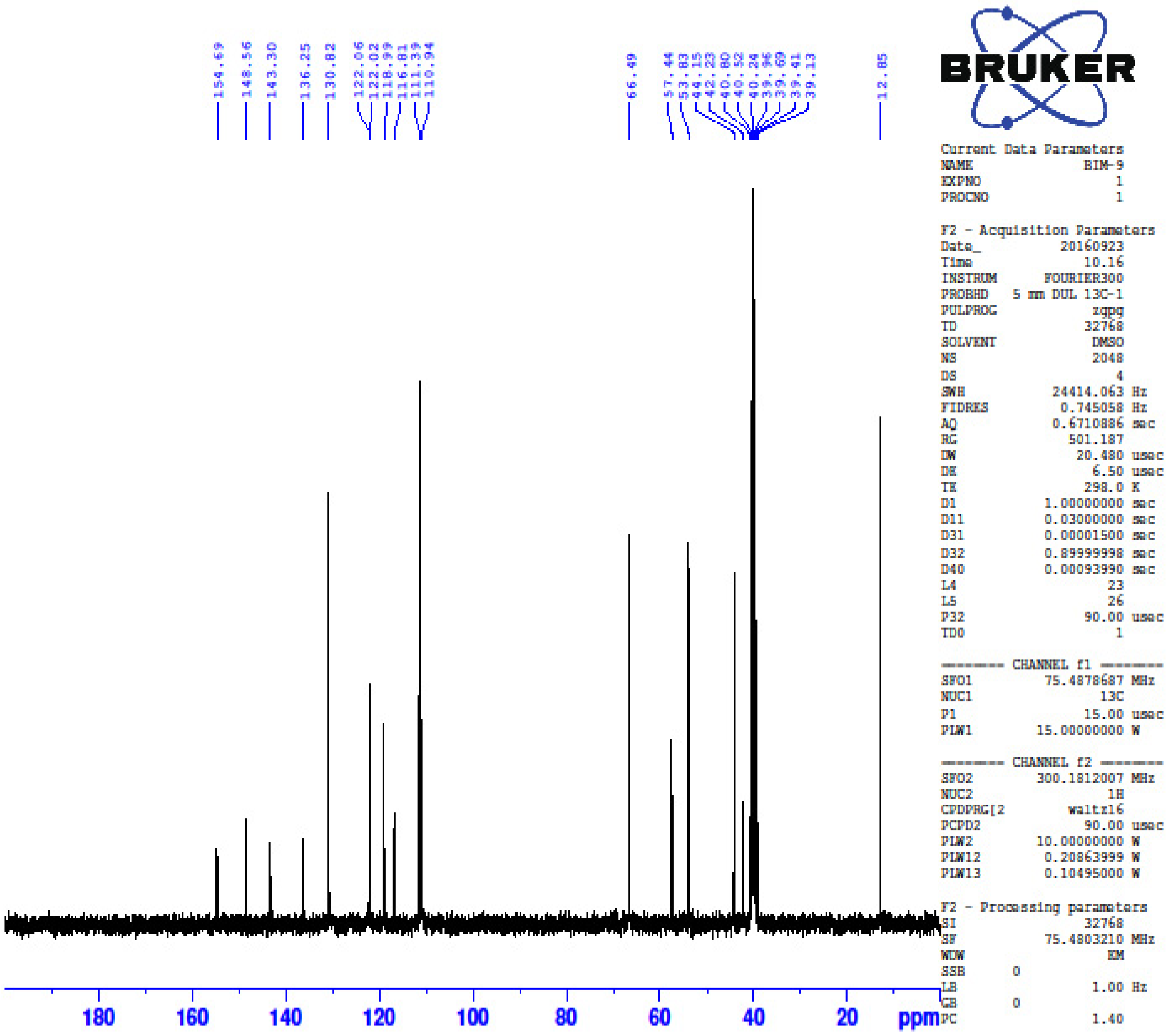

^{1}H-NMR spectrum of *2-(4-Diethylaminophenyl)-1-[2-(morpholin-4-yl) ethyl]-1H-benzimidazole (**2h**)*

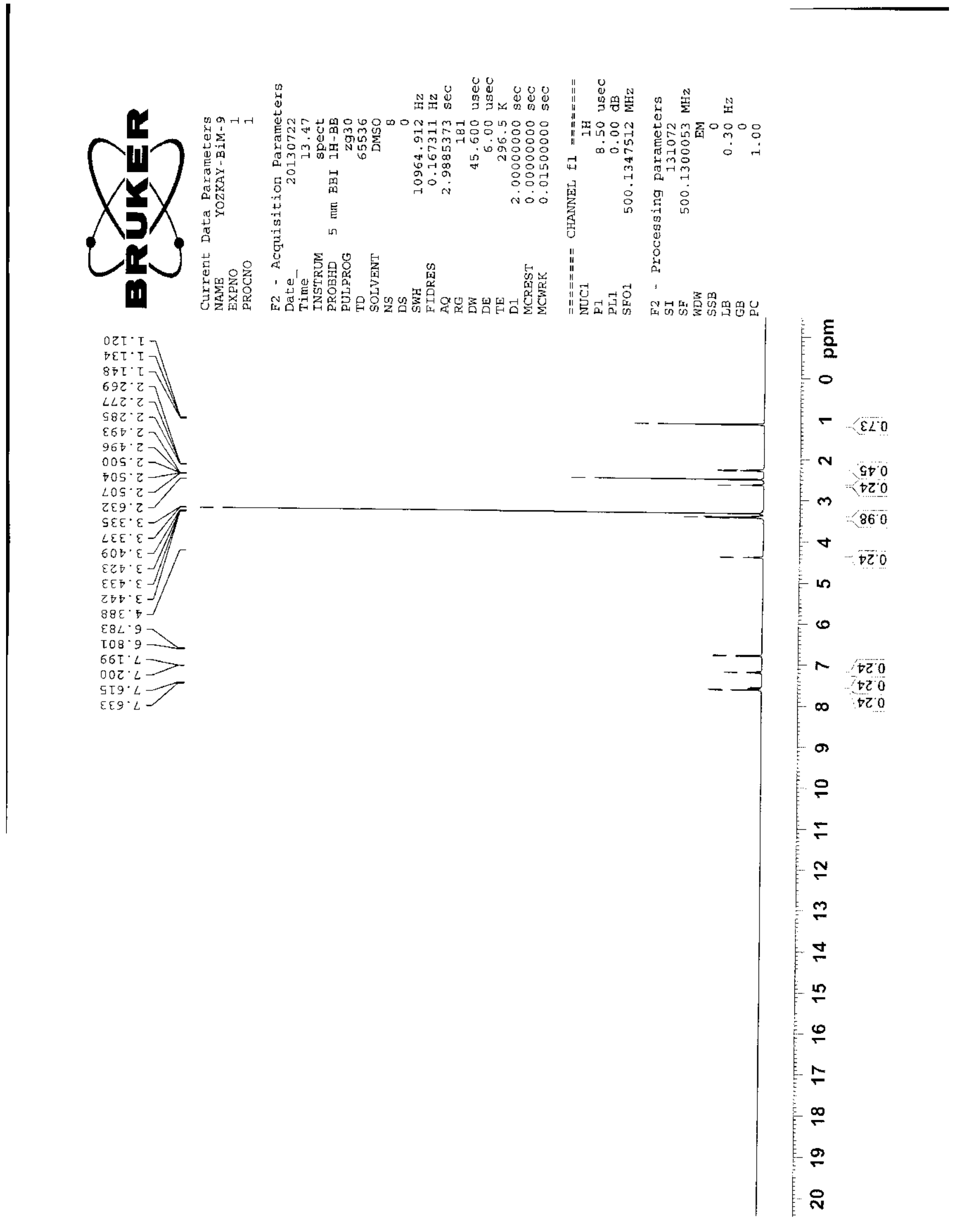

FTIR spectrum of *2-(4-Diethylaminophenyl)-1-[2-(morpholin-4-yl) ethyl]-1H-benzimidazole* ***(2h)***

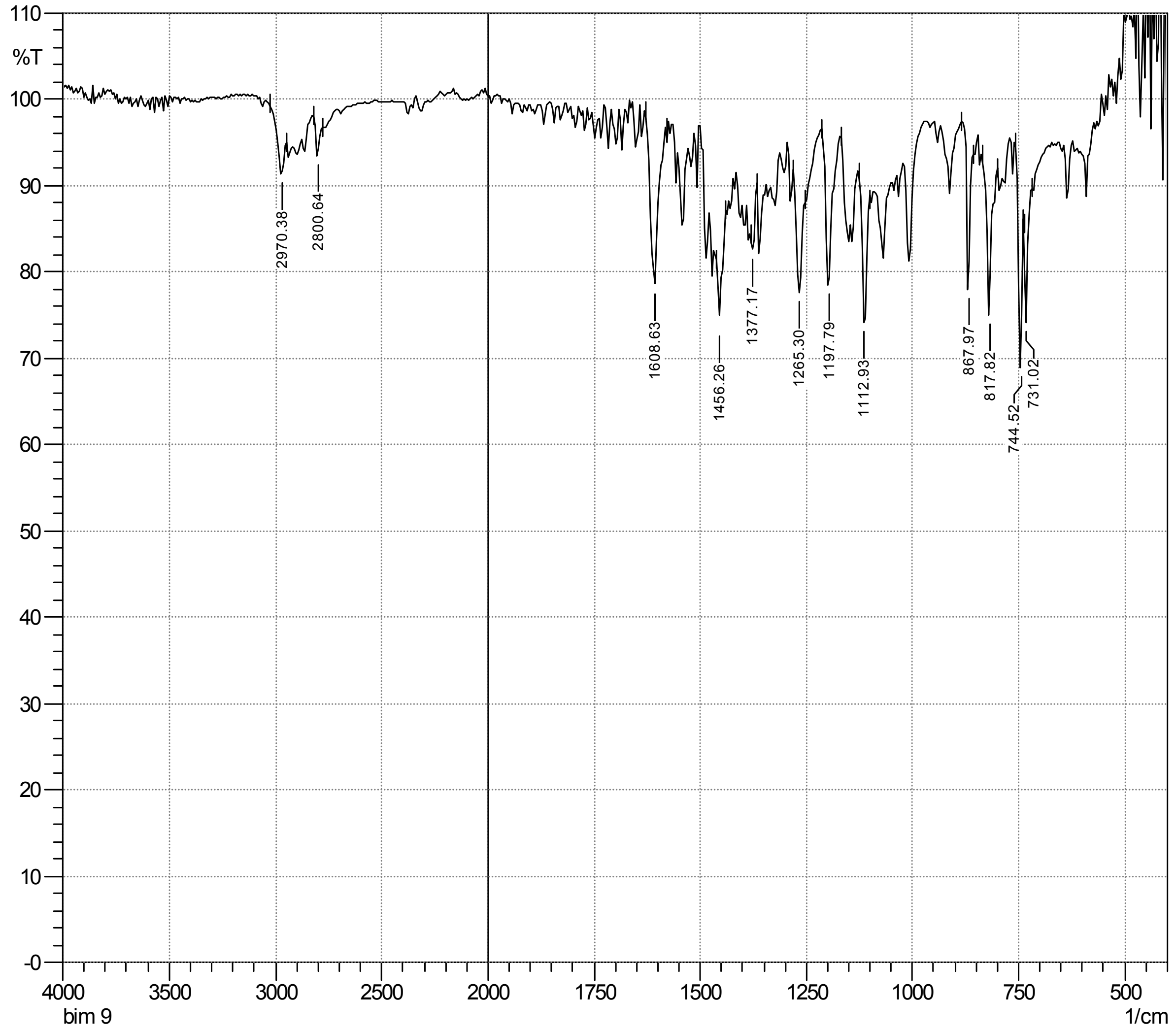

HRMS spectrum of *2-(4-Diethylaminophenyl)-1-[2-(morpholin-4-yl) ethyl]-1H-benzimidazole (**2h**)*

Data File: C:\LabSolutions\Data\Analiz\Bim serisi\Bim-9_17.lcd

Elmt	Val.	Min	Max	Elmt	Val.	Min	Max	Elmt	Val.	Min	Max	Use Adduct
H	1	14	30	O	2	1	5	Cl	1	0	0	H
C	4	12	30	F	1	0	3	Br	1	0	1	
N	3	3	4	S	2	0	0					

Error Margin (ppm):	10	DBE Range:	-2.0 - 1000.0	Electron Ions:	both
HC Ratio:	unlimited	Apply N Rule:	yes	Use MSn Info:	no
Max Isotopes:	3	Isotope RI (%):	1.00	Isotope Res:	10000
MSn Iso RI (%):	10.00	MSn Logic Mode:	AND	Max Results:	500

Event#: 1 MS(E+) Ret. Time : 5.040 -> 5.133 Scan# : 757 -> 771

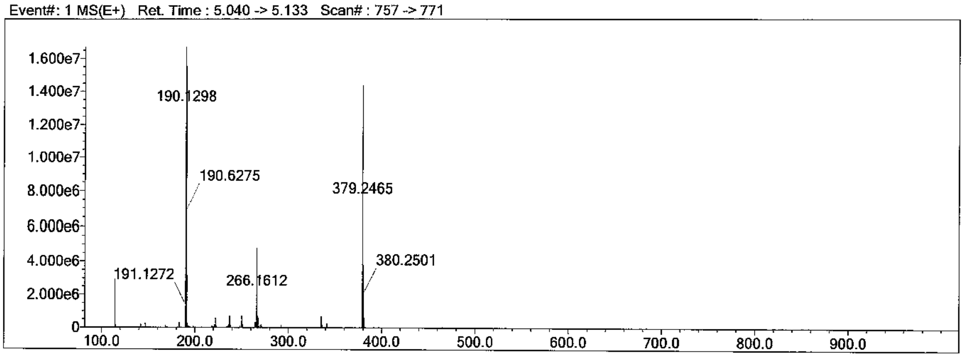

Measured region for 379.2465 m/z

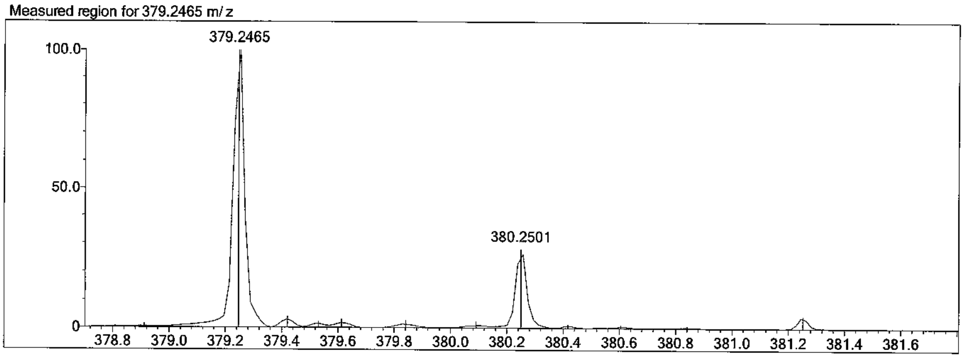

C23 H30 N4 O [M+H]+ : Predicted region for 379.2492 m/z

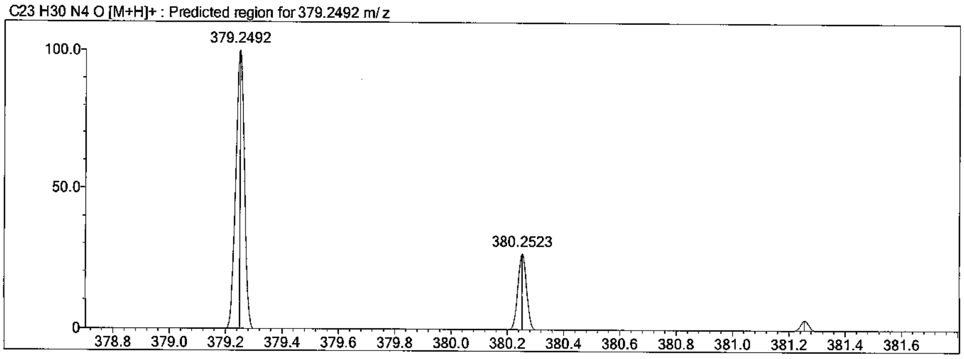

Rank	Score	Formula (M)	Ion	Meas. m/z	Pred. m/z	Df. (mDa)	Df. (ppm)	Iso	DBE
1	68.41	C23 H30 N4 O	[M+H]+	379.2465	379.2492	-2.7	-7.12	99.44	11.0

^{13}C-NMR spectrum of *2-(4-Dimethylaminophenyl)-1-[2-(morpholin-4-yl) ethyl]-1H-benzimidazole* ***(2i)***

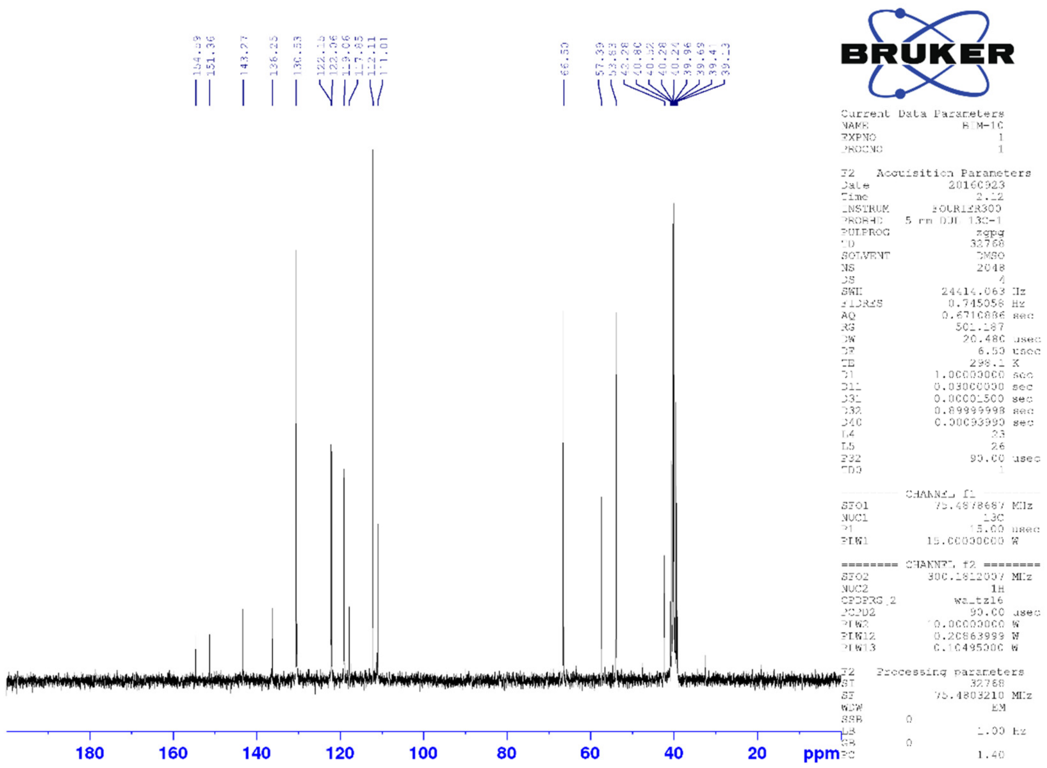

^{1}H-NMR spectrum of *2-(4-Dimethylaminophenyl)-1-[2-(morpholin-4-yl) ethyl]-1H-benzimidazole (2i)*

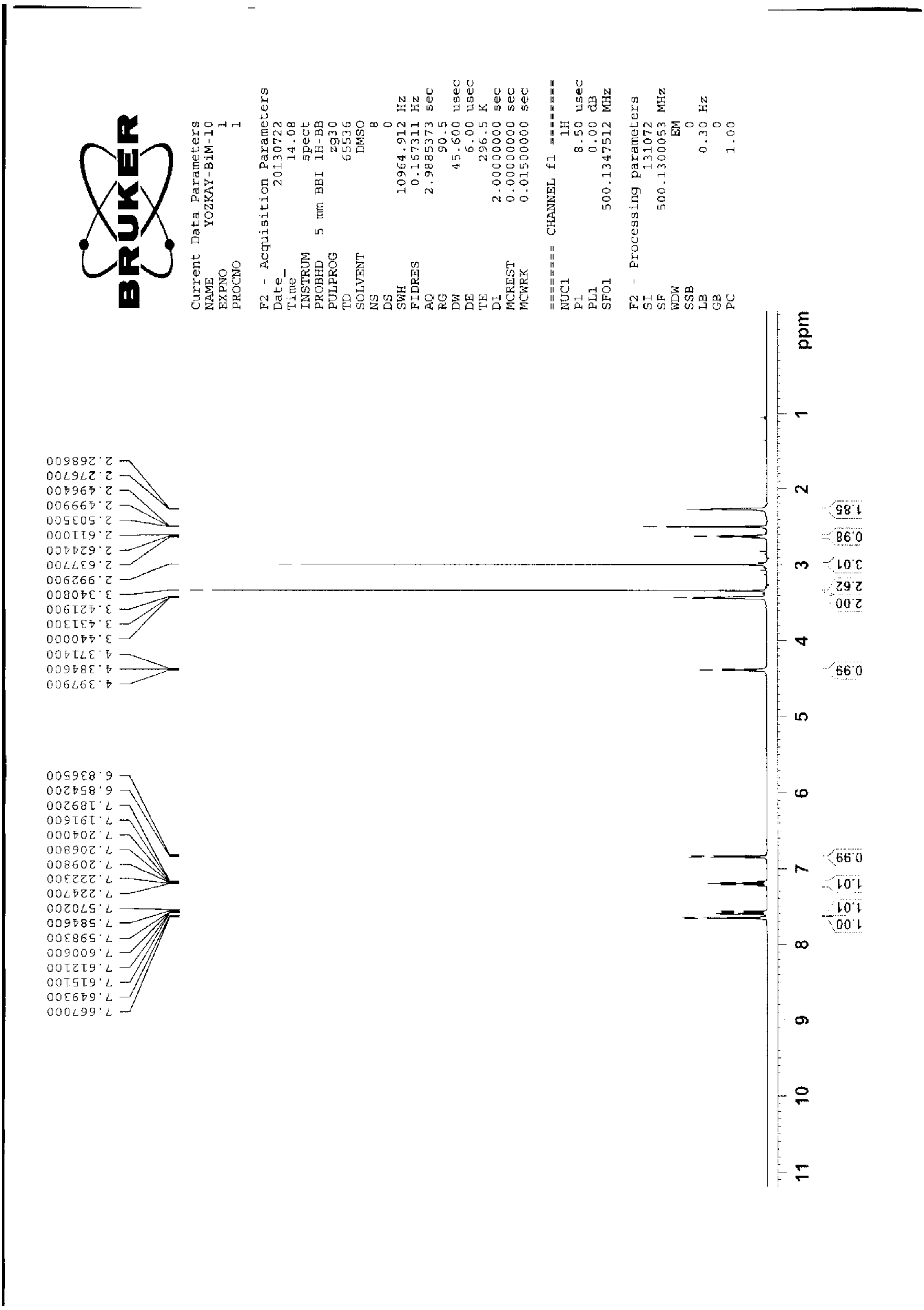

FTIR spectrum of *2-(4-Dimethylaminophenyl)-1-[2-(morpholin-4-yl) ethyl]-1H-benzimidazole (**2i**)*

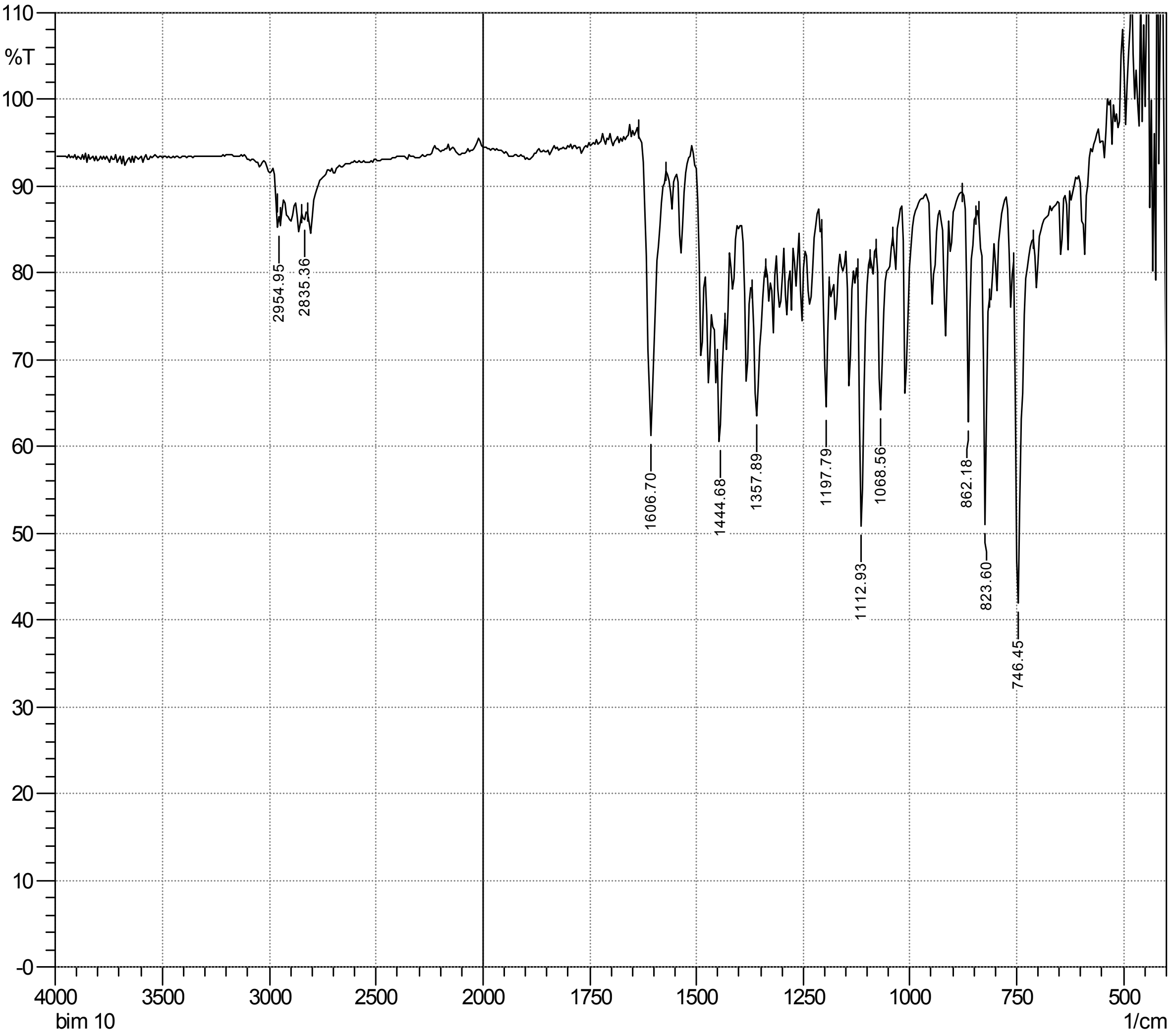

HRMS spectrum of *2-(4-Dimethylaminophenyl)-1-[2-(morpholin-4-yl) ethyl]-1H-benzimidazole* (***2i***)

Data File: C:\LabSolutions\Data\Analiz\Bim serisi\Bim-10_18.lcd

Elmt	Val.	Min	Max	Elmt	Val.	Min	Max	Elmt	Val.	Min	Max	Use Adduct
H	1	14	30	O	2	1	5	Cl	1	0	0	H
C	4	12	30	F	1	0	0	Br	1	0	1	
N	3	3	4	S	2	0	0					

Error Margin (ppm):	10	DBE Range:	-2.0 - 1000.0	Electron Ions:	both
HC Ratio:	unlimited	Apply N Rule:	yes	Use MSn Info:	no
Max Isotopes:	3	Isotope RI (%):	1.00	Isotope Res:	10000
MSn Iso RI (%):	10.00	MSn Logic Mode:	AND	Max Results:	500

Event#: 1 MS(E+) Ret. Time : 4.587 -> 4.613 Scan# : 689 -> 693

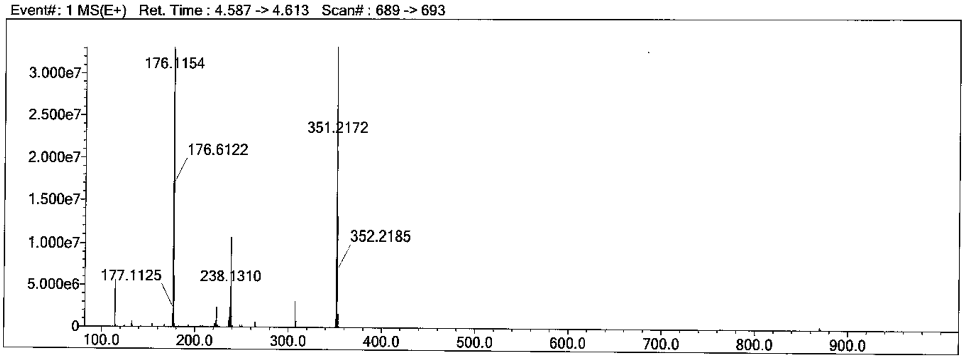

Measured region for 351.2172 m/z

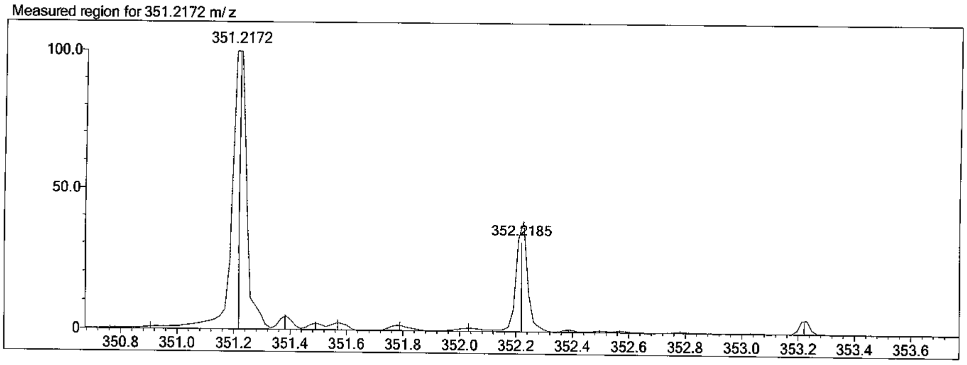

C21 H26 N4 O [M+H]+ : Predicted region for 351.2179 m/z

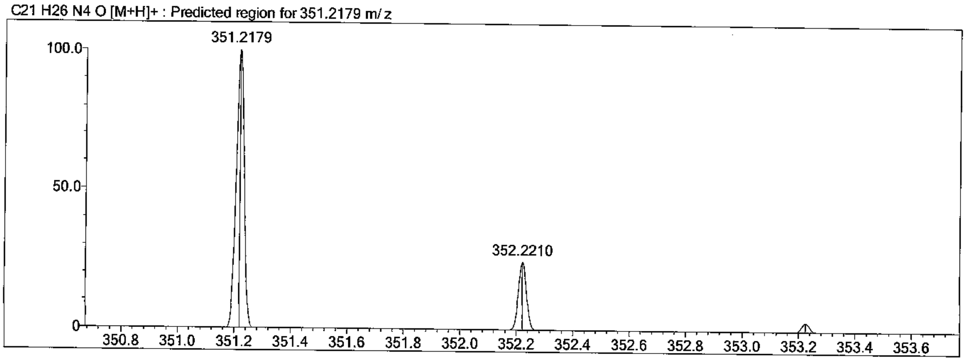

Rank	Score	Formula (M)	Ion	Meas. m/z	Pred. m/z	Df. (mDa)	Df. (ppm)	Iso	DBE
1	70.70	C21 H26 N4 O	[M+H]+	351.2172	351.2179	-0.7	-1.99	72.49	11.0

^{13}C-NMR spectrum of *2-(4-Ethoxyphenyl)-1-[2-(morpholin-4-yl) ethyl]-1H-benzimidazole* (***2j***)

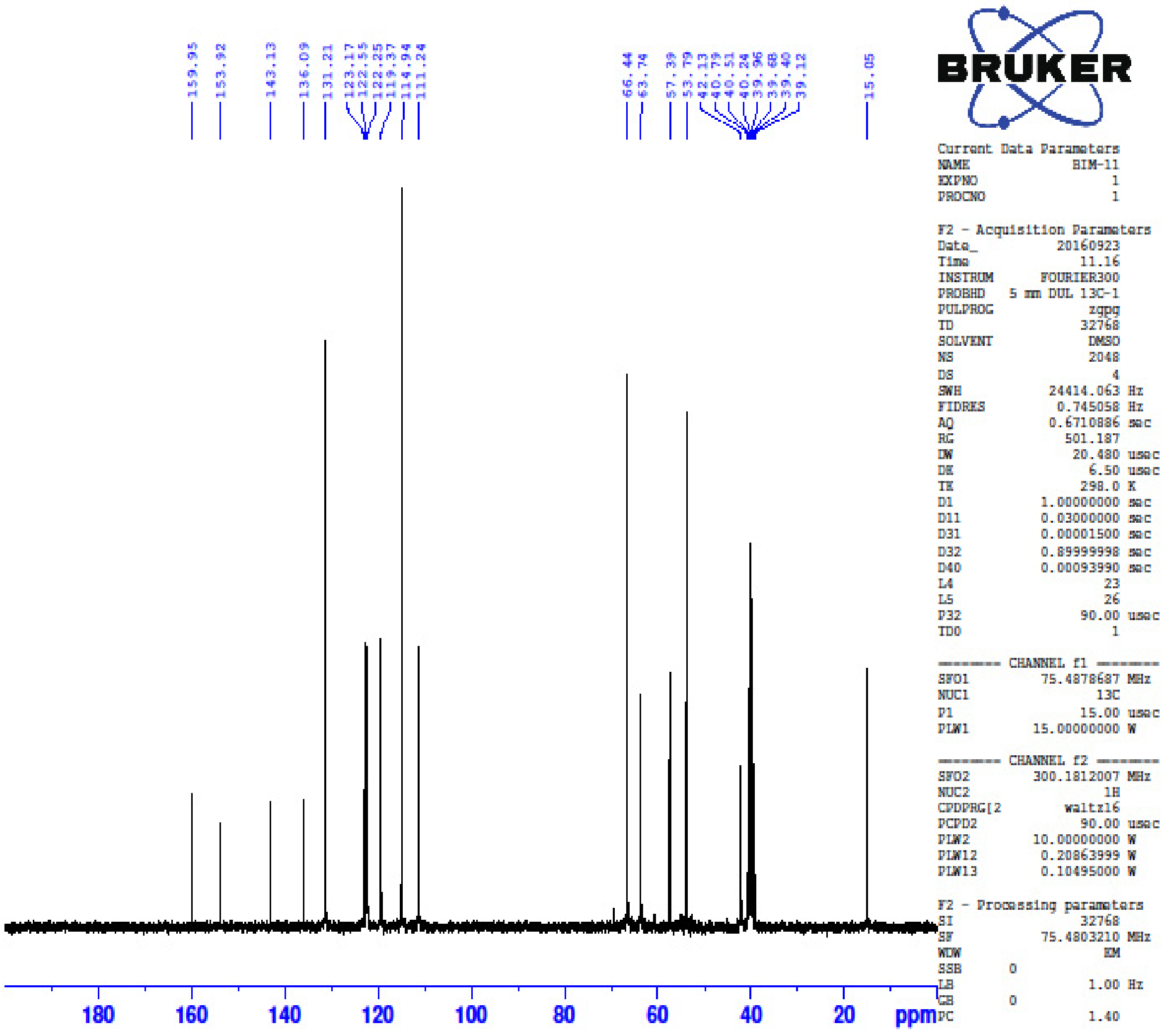

^{1}H-NMR spectrum of *2-(4-Ethoxyphenyl)-1-[2-(morpholin-4-yl) ethyl]-1H-benzimidazole* (***2j***)

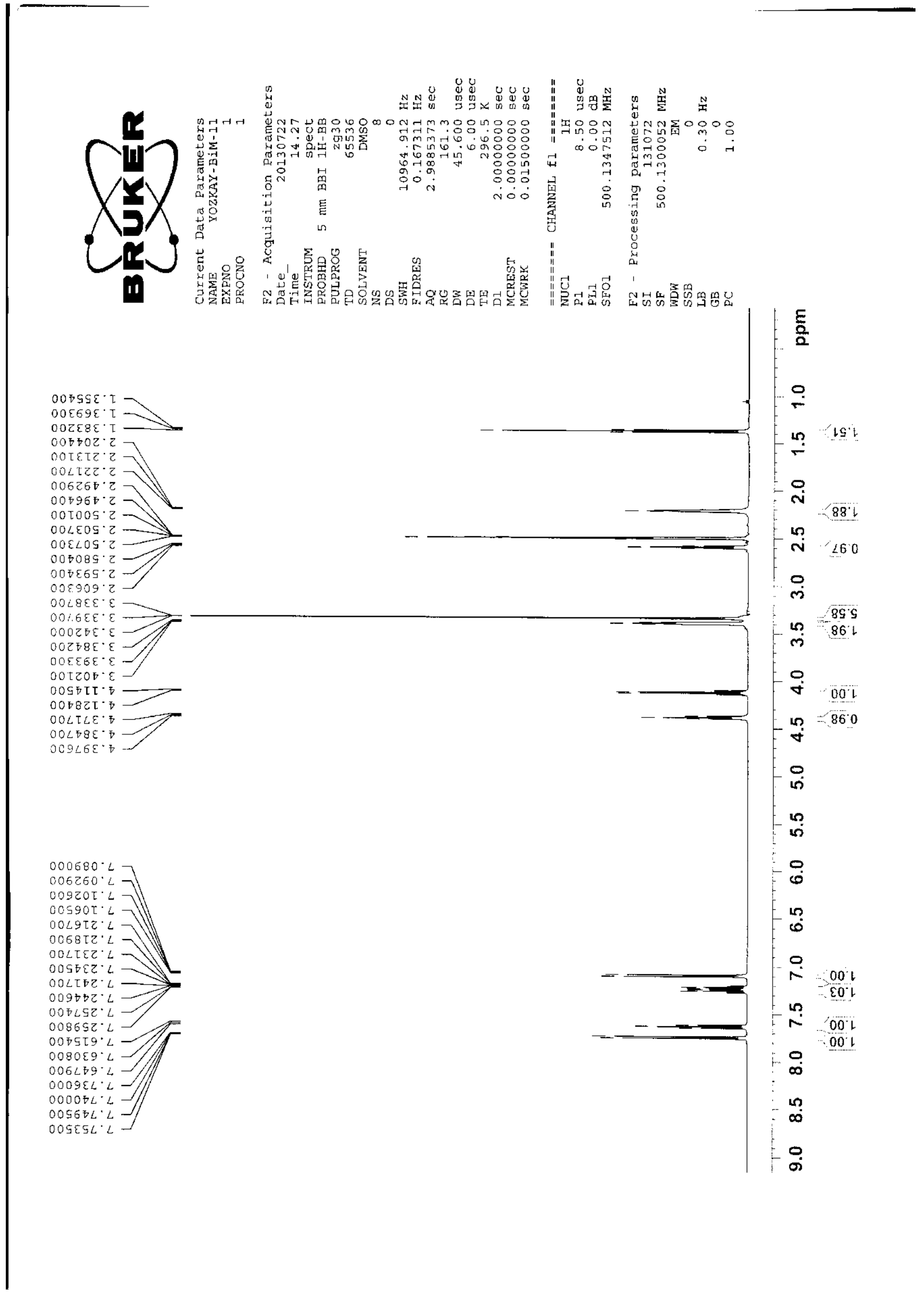

FTIR spectrum of *2-(4-Ethoxyphenyl)-1-[2-(morpholin-4-yl) ethyl]-1H-benzimidazole* (***2j***)

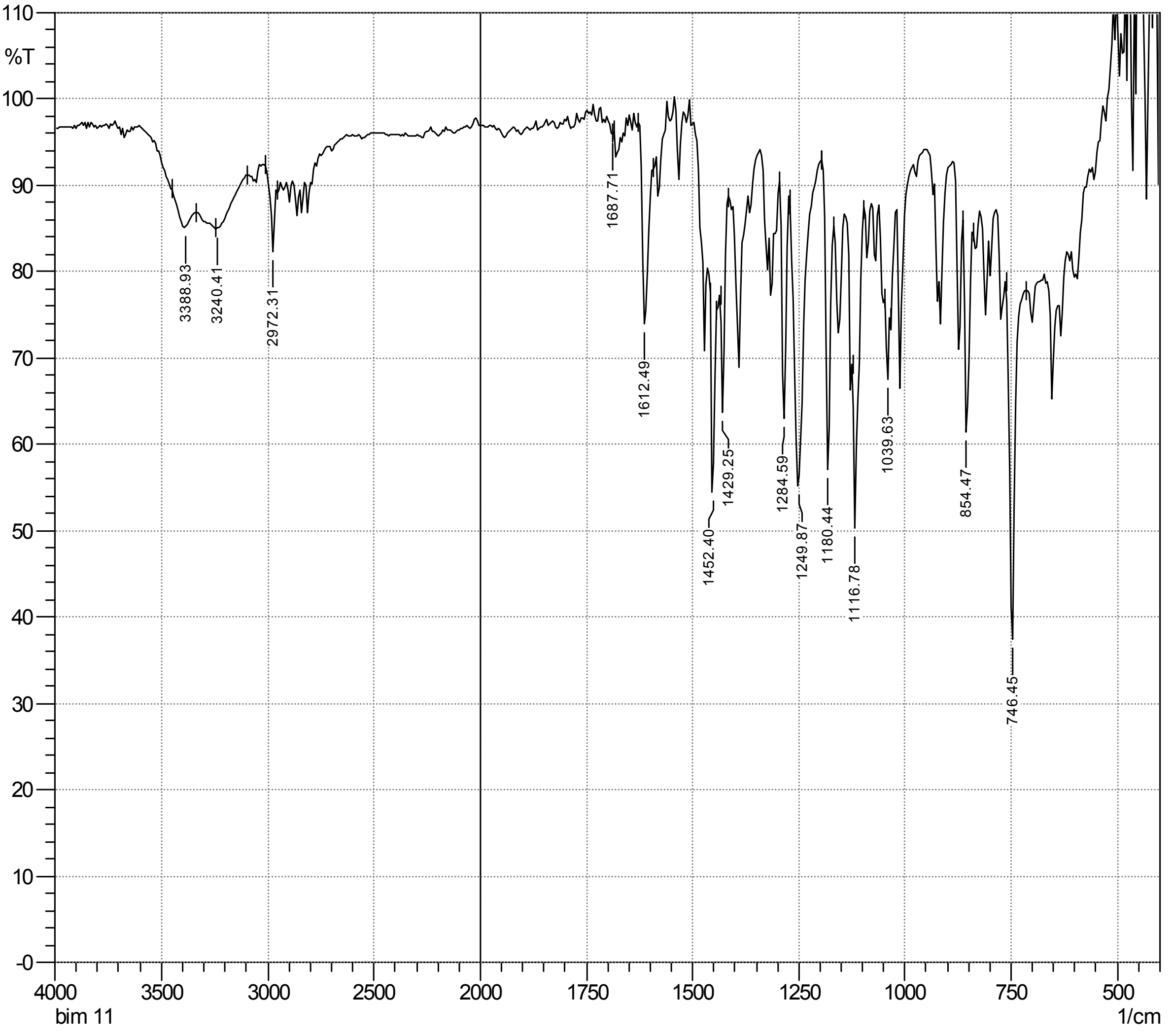

HRMS spectrum of *2-(4-Ethoxyphenyl)-1-[2-(morpholin-4-yl) ethyl]-1H-benzimidazole (**2j**)*

Data File: C:\ LabSolutions\ Data\Analiz\Bim serisi\ Bim-11_01.lcd

Elmt	Val.	Min	Max	Elmt	Val.	Min	Max	Elmt	Val.	Min	Max	Use Adduct
H	1	14	30	O	2	1	5	Cl	1	0	0	H
C	4	12	30	F	1	0	0	Br	1	0	1	
N	3	3	4	S	2	0	0					

Error Margin (ppm):	15	DBE Range:	-2.0 - 1000.0	Electron Ions:	both
HC Ratio:	unlimited	Apply N Rule:	yes	Use MSn Info:	no
Max Isotopes:	3	Isotope RI (%):	1.00	Isotope Res:	10000
MSn Iso RI (%):	10.00	MSn Logic Mode:	AND	Max Results:	500

Event#: 1 MS(E+) Ret. Time : 4.720 -> 4.800 Scan# : 709 -> 721

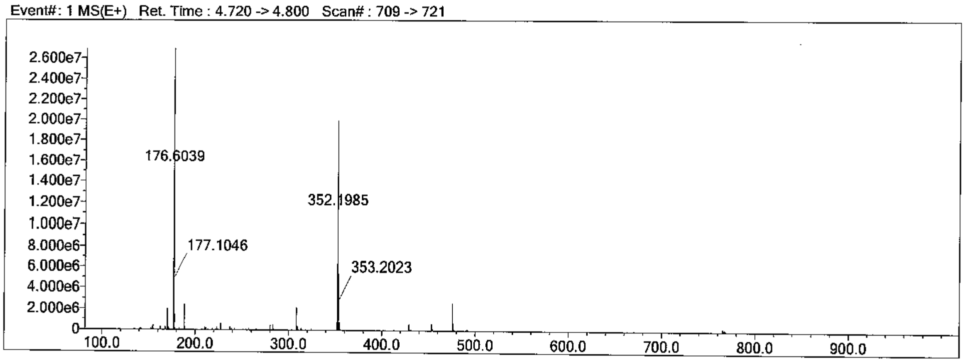

Measured region for 352.1985 m/z

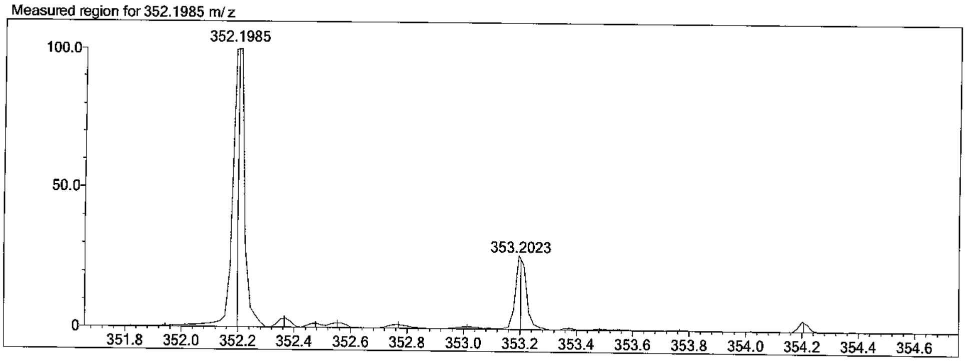

C21 H25 N3 O2 [M+H]+ : Predicted region for 352.2020 m/z

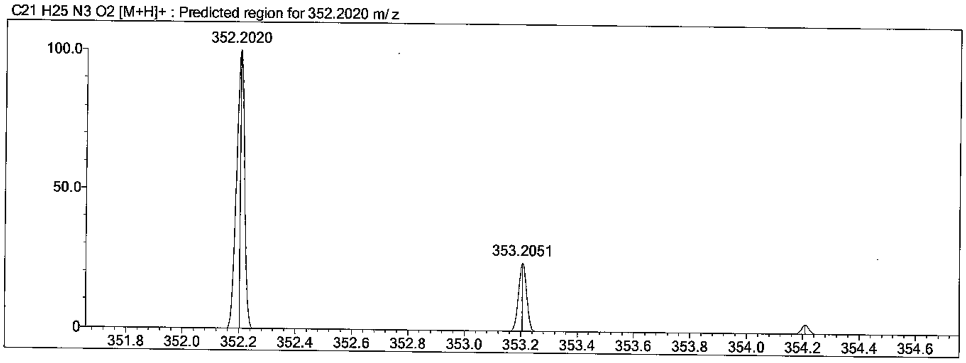

Rank	Score	Formula (M)	Ion	Meas. m/z	Pred. m/z	Df. (mDa)	Df. (ppm)	Iso	DBE
1	35.22	C21 H25 N3 O2	[M+H]+	352.1985	352.2020	-3.5	-9.94	86.76	11.0

^{13}C-NMR of *2-(4-Cyanophenyl)-1-[2-(morpholin-4-yl)ethyl]-1H-benzimidazole* (***2k***)

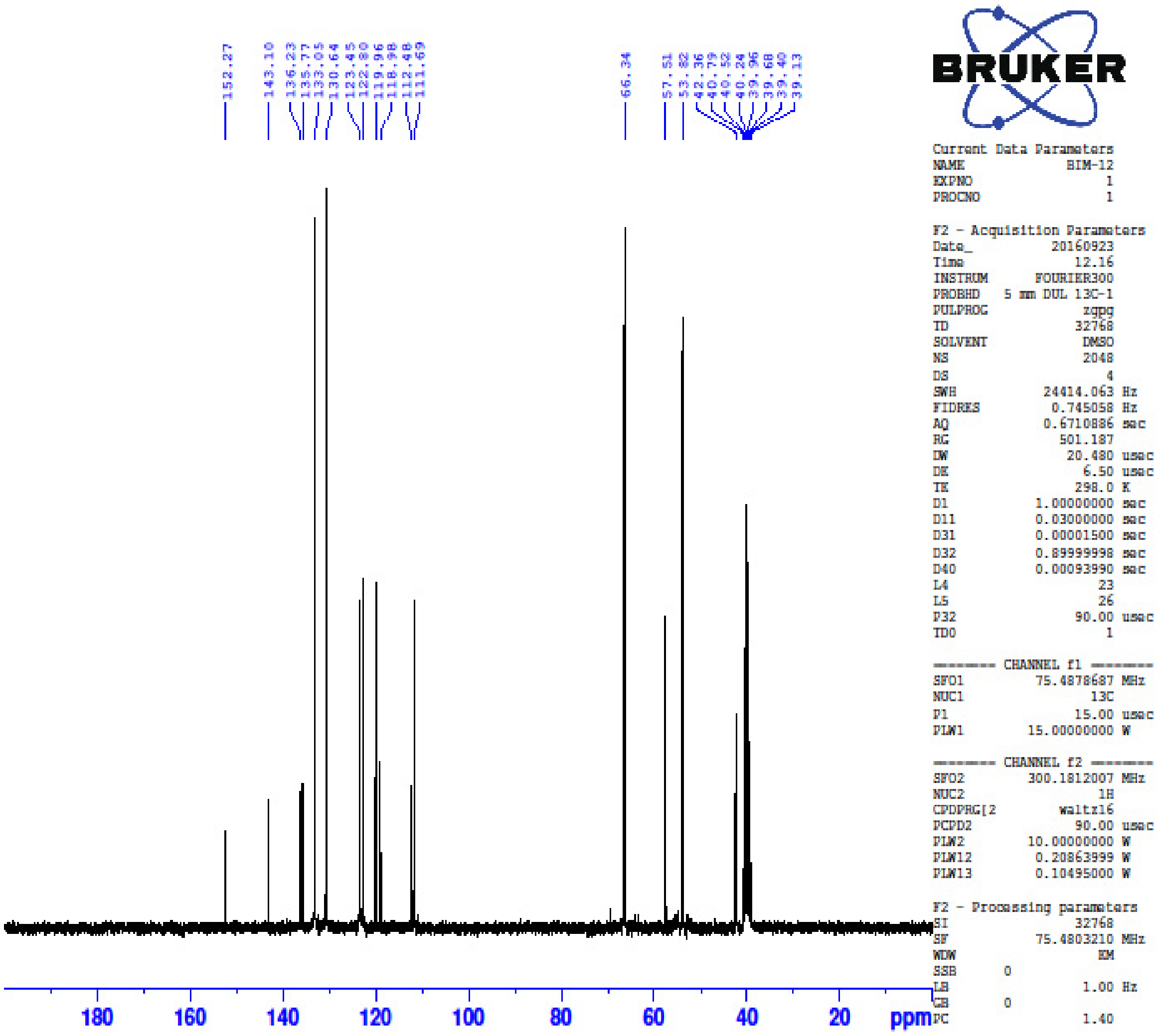

^{1}H-NMR spectrum of *2-(4-Cyanophenyl)-1-[2-(morpholin-4-yl)ethyl]-1H-benzimidazole* (**2k**)

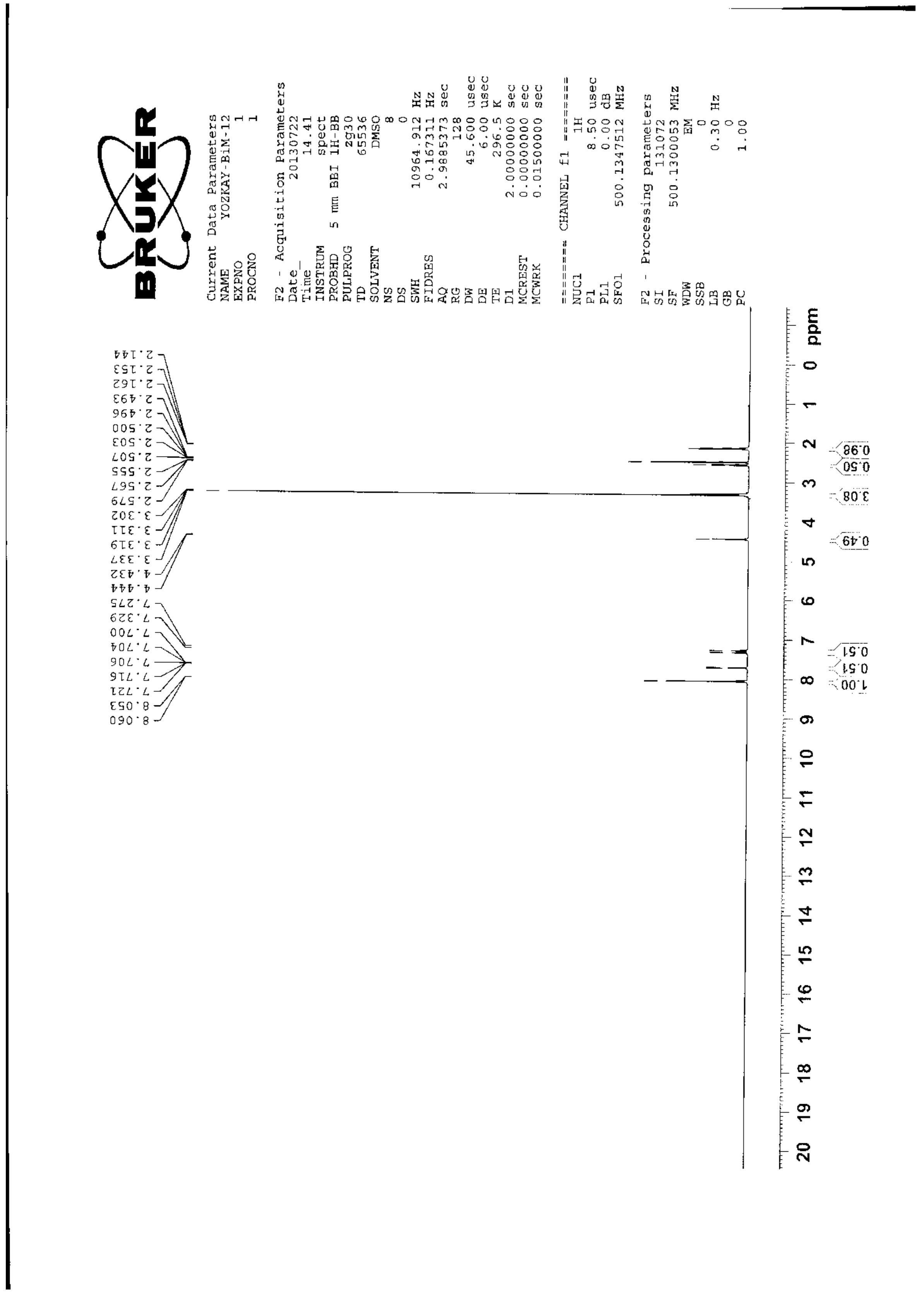

FTIR spectrum of *2-(4-Cyanophenyl)-1-[2-(morpholin-4-yl)ethyl]-1H-benzimidazole* (***2k***)

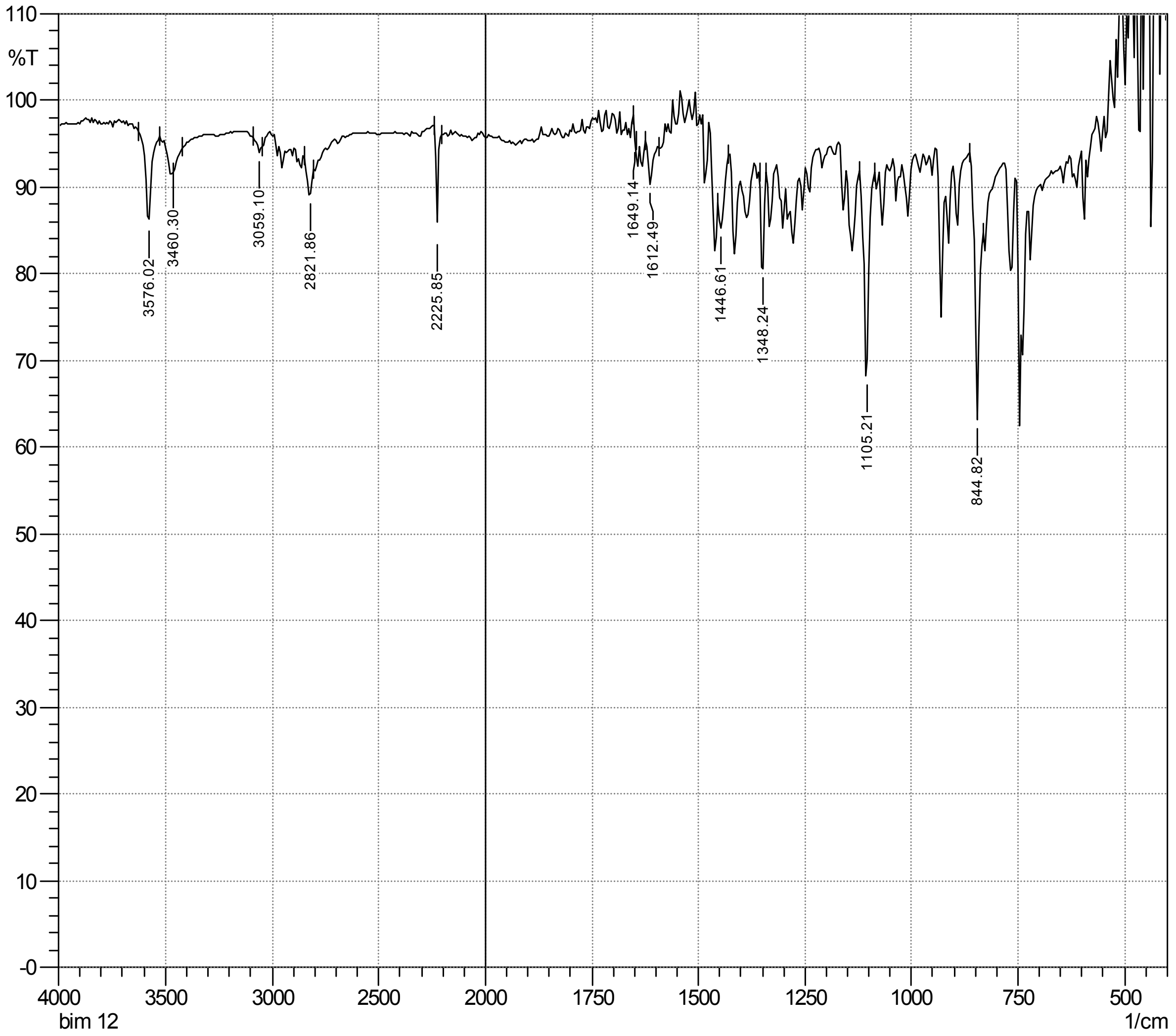

HRMS spectrum of *2-(4-Cyanophenyl)-1-[2-(morpholin-4-yl)ethyl]-1H-benzimidazole* (***2k***)

Formula Predictor Report - Bim-12_20.lcd

Data File: C:\ LabSolutions\ Data\Analiz\Bim serisi\ Bim-12_20.lcd

Elmt	Val.	Min	Max	Elmt	Val.	Min	Max	Elmt	Val.	Min	Max	Use Adduct
H	1	14	30	O	2	1	5	Cl	1	0	0	H
C	4	12	30	F	1	0	0	Br	1	0	1	
N	3	3	4	S	2	0	0					

Error Margin (ppm):	10	DBE Range:	-2.0 - 1000.0	Electron Ions:	both
HC Ratio:	unlimited	Apply N Rule:	yes	Use MSn Info:	no
Max Isotopes:	3	Isotope RI (%):	1.00	Isotope Res:	10000
MSn Iso RI (%):	10.00	MSn Logic Mode:	AND	Max Results:	500

Event#: 1 MS(E+) Ret. Time : 4.760 -> 4.813 Scan# : 715 -> 723

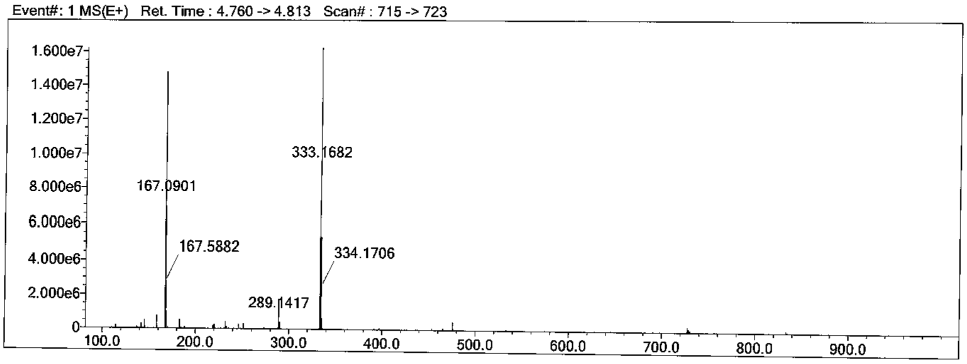

Measured region for 333.1682 m/z

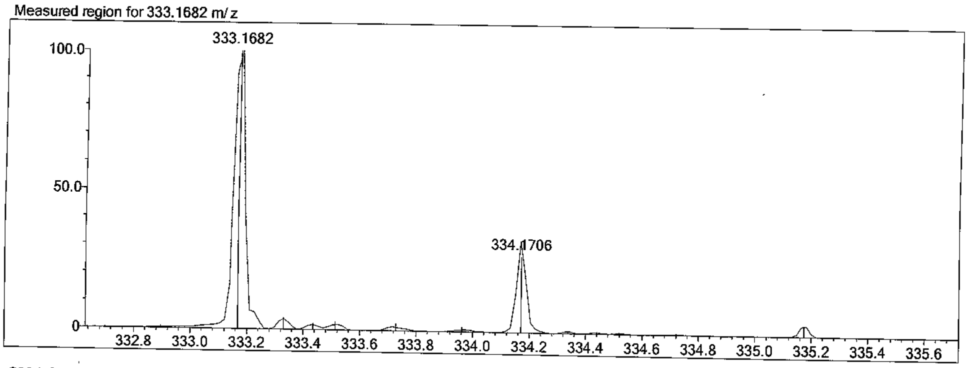

C20 H20 N4 O [M+H]+ : Predicted region for 333.1710 m/z

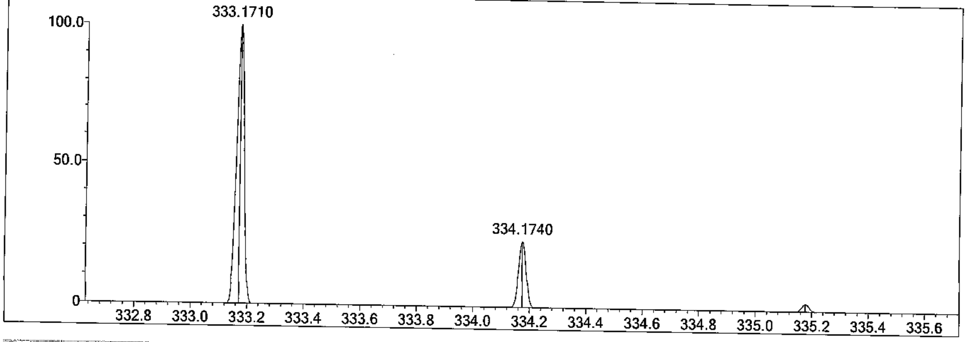

Rank	Score	Formula (M)	Ion	Meas. m/z	Pred. m/z	Df. (mDa)	Df. (ppm)	Iso	DBE
1	49.12	C20 H20 N4 O	[M+H]+	333.1682	333.1710	-2.8	-8.40	87.71	13.0

^{13}C-NMR spectrum of *2-(4-Trifluoromethylphenyl)-1-[2-(morpholin-4-yl)ethyl]-1H-benzimidazole* ***(2l)***

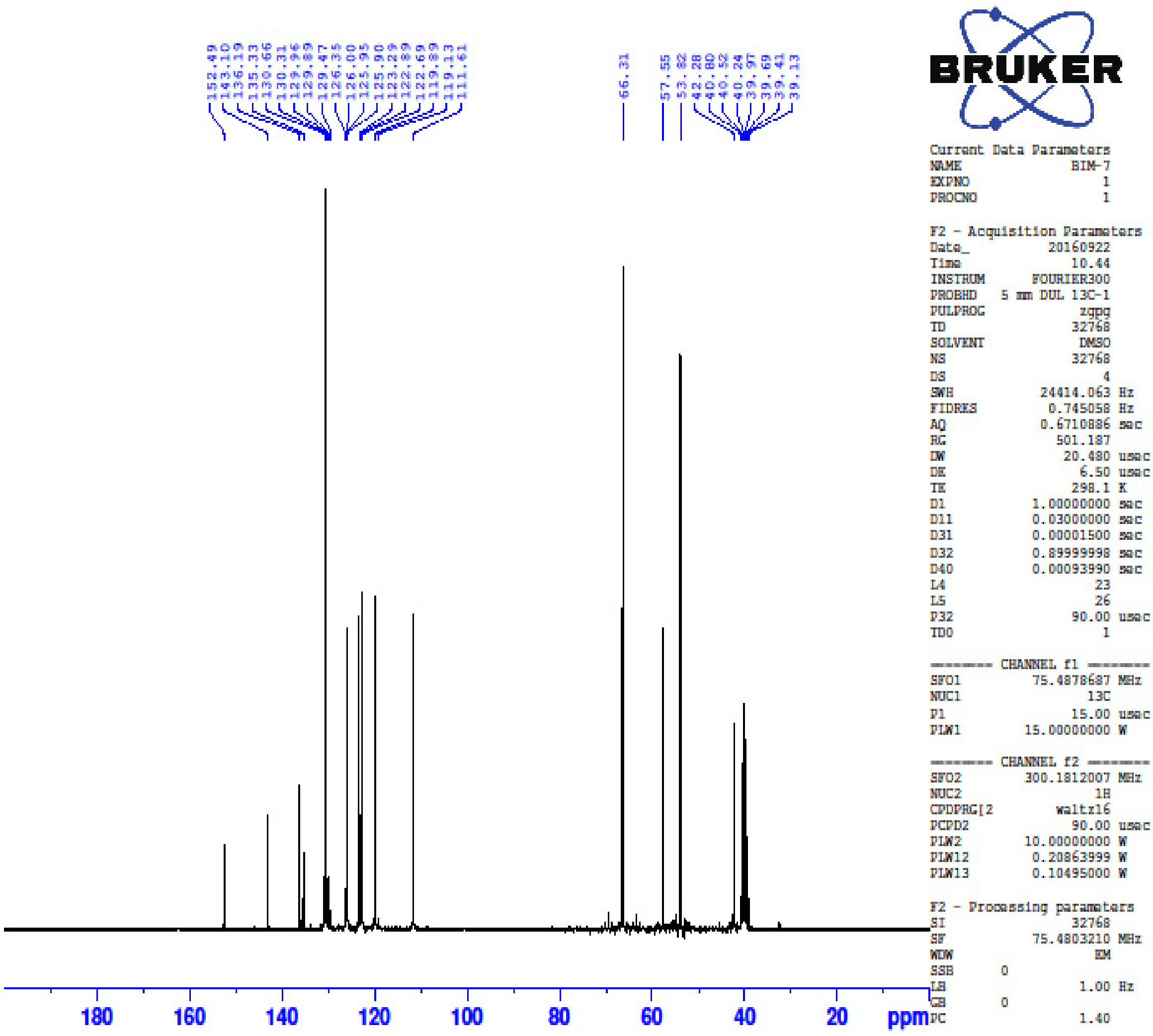

^{1}H-NMR spectrum of *2-(4-Trifluoromethylphenyl)-1-[2-(morpholin-4-yl)ethyl]-1H-benzimidazole (2l)*

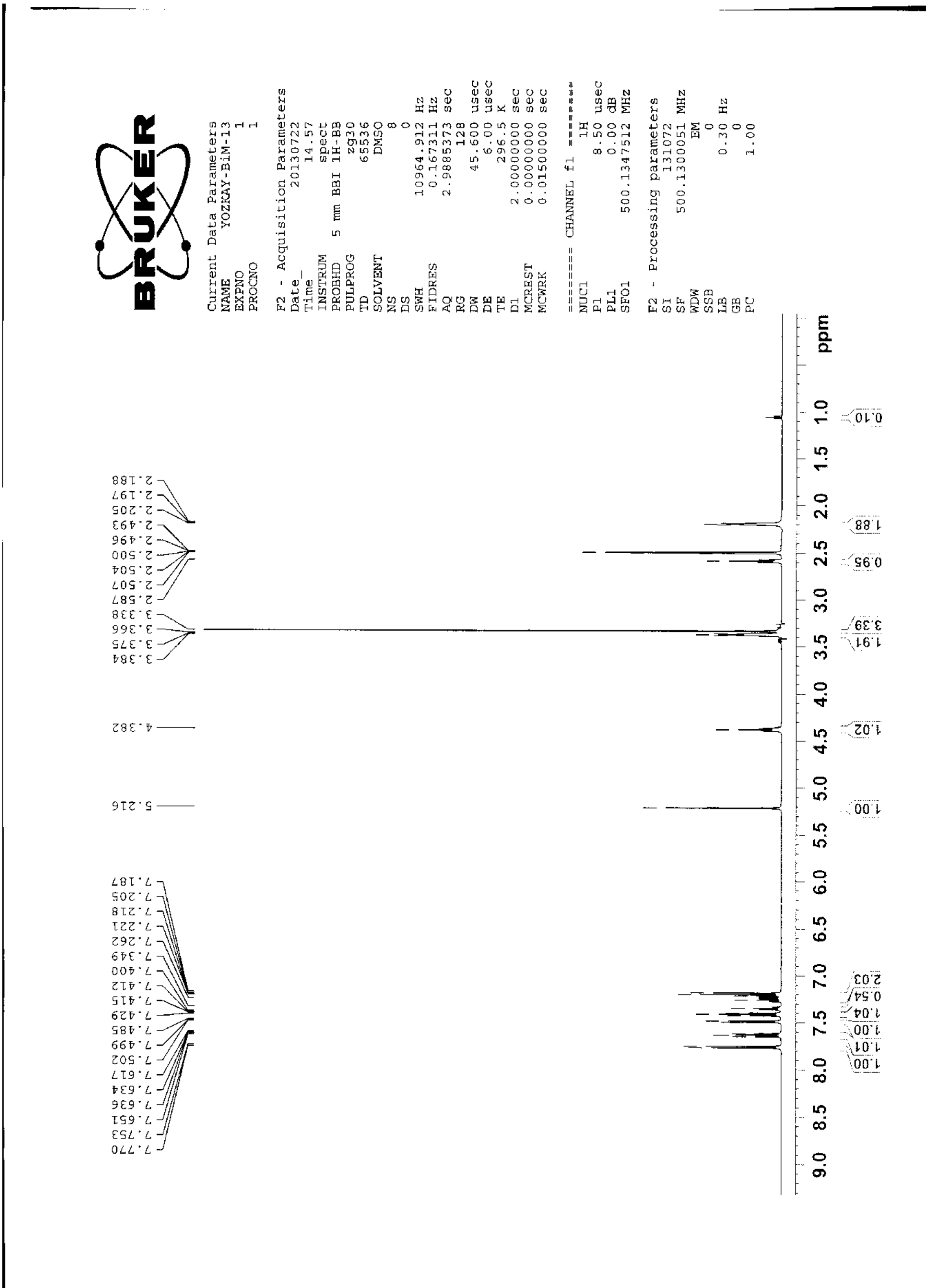

FTIR spectrum of *2-(4-Trifluoromethylphenyl)-1-[2-(morpholin-4-yl)ethyl]-1H-benzimidazole* ***(2l)***

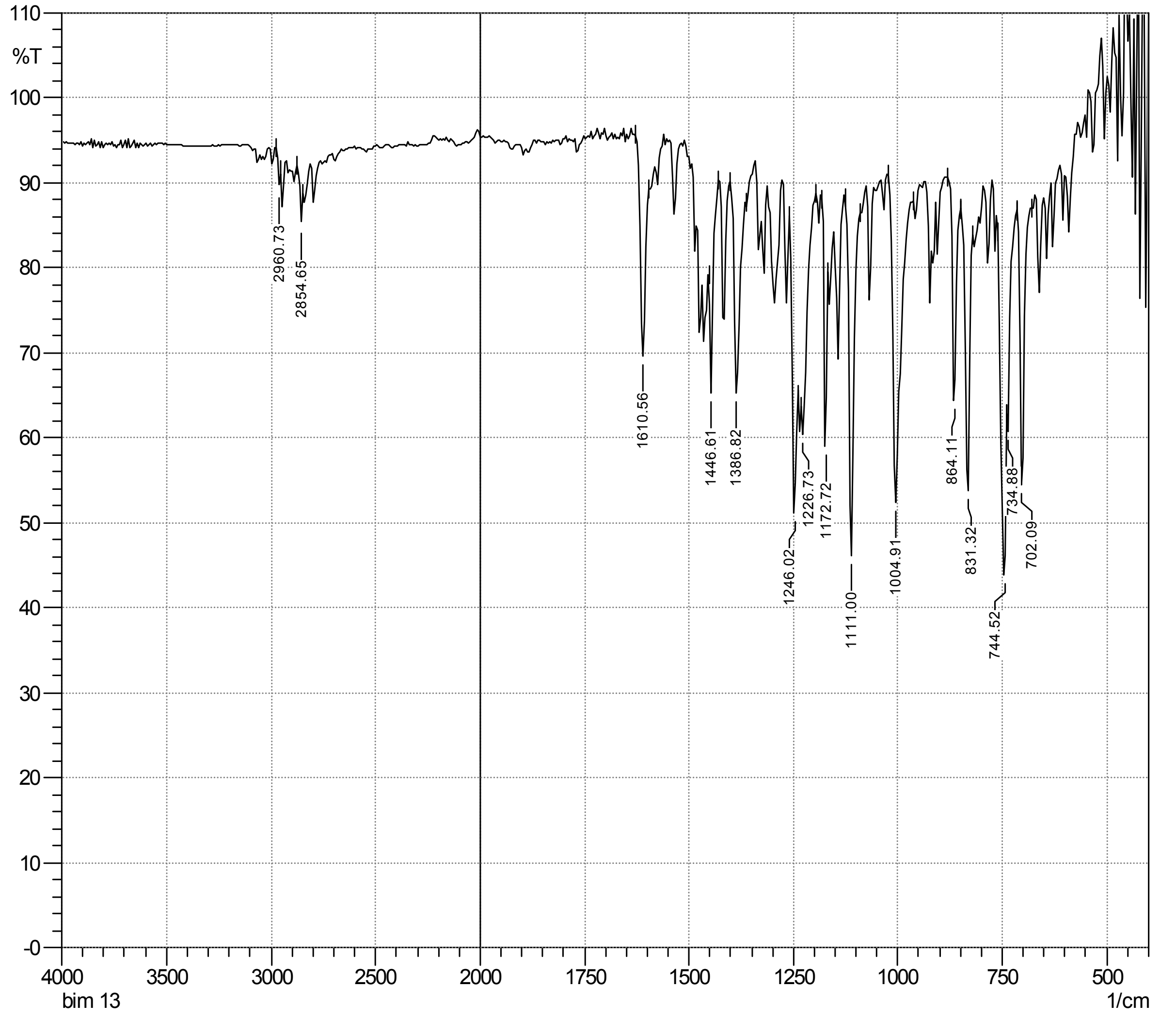

HRMS spectrum of *2-(4-Trifluoromethylphenyl)-1-[2-(morpholin-4-yl)ethyl]-1H-benzimidazole* (**2l**)

Data File: C:\ LabSolutions\ Data\Analiz\Bim serisi\ Bim-13_21.lcd

Elmt	Val.	Min	Max	Elmt	Val.	Min	Max	Elmt	Val.	Min	Max	Use Adduct
H	1	14	30	O	2	1	5	Cl	1	0	0	H
C	4	12	30	F	1	0	0	Br	1	0	1	
N	3	3	4	S	2	0	0					

Error Margin (ppm):	10	DBE Range:	-2.0 - 1000.0	Electron Ions:	both
HC Ratio:	unlimited	Apply N Rule:	yes	Use MSn Info:	no
Max Isotopes:	3	Isotope RI (%):	1.00	Isotope Res:	10000
MSn Iso RI (%):	10.00	MSn Logic Mode:	AND	Max Results:	500

Event#: 1 MS(E+) Ret. Time : 5.533 -> 5.613 Scan# : 831 -> 843

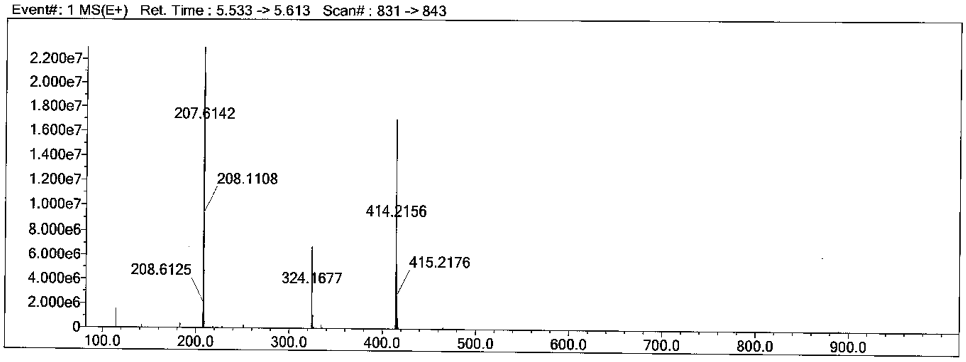

Measured region for 414.2156 m/z

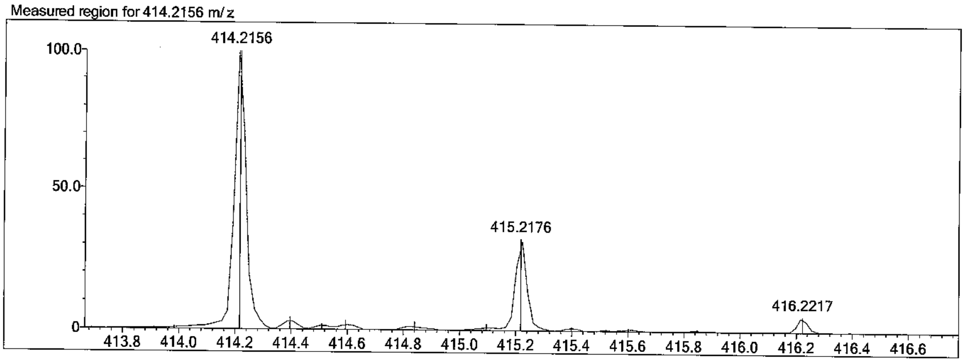

C26 H27 N3 O2 [M+H]+ : Predicted region for 414.2176 m/z

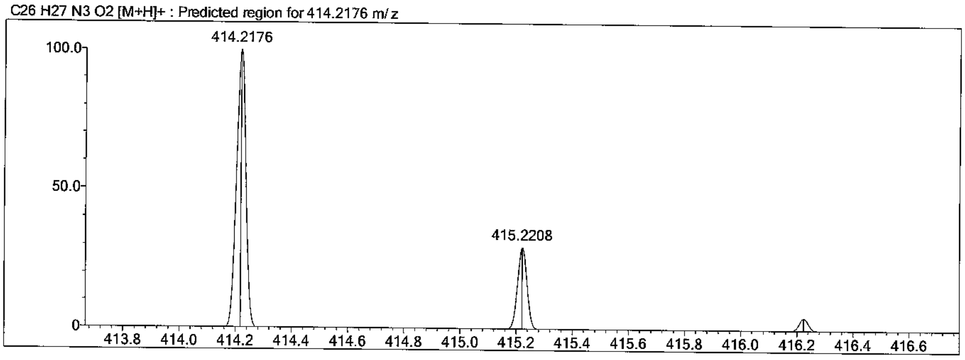

Rank	Score	Formula (M)	Ion	Meas. m/z	Pred. m/z	Df. (mDa)	Df. (ppm)	Iso	DBE
1	67.55	C26 H27 N3 O2	[M+H]+	414.2156	414.2176	-2.0	-4.83	74.71	15.0

^{13}C-NMR of *2-(4-Trifluoromethoxyphenyl)-1-[2-(morpholin-4-yl)ethyl]-1H-benzimidazole* (***2m***)

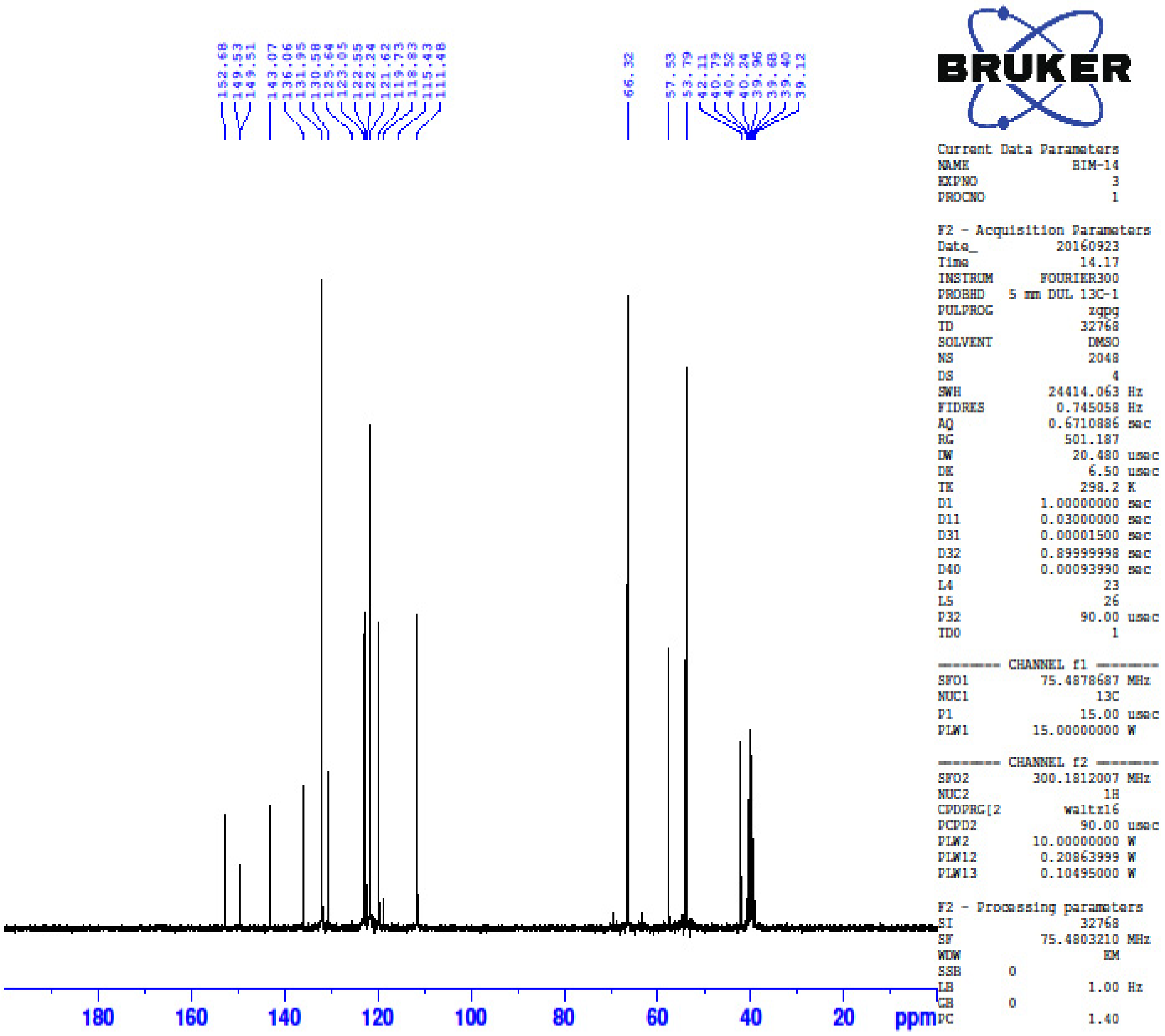

^{1}H-NMR spectrum of *2-(4-Trifluoromethoxyphenyl)-1-[2-(morpholin-4-yl)ethyl]-1H-benzimidazole* ***(2m)***

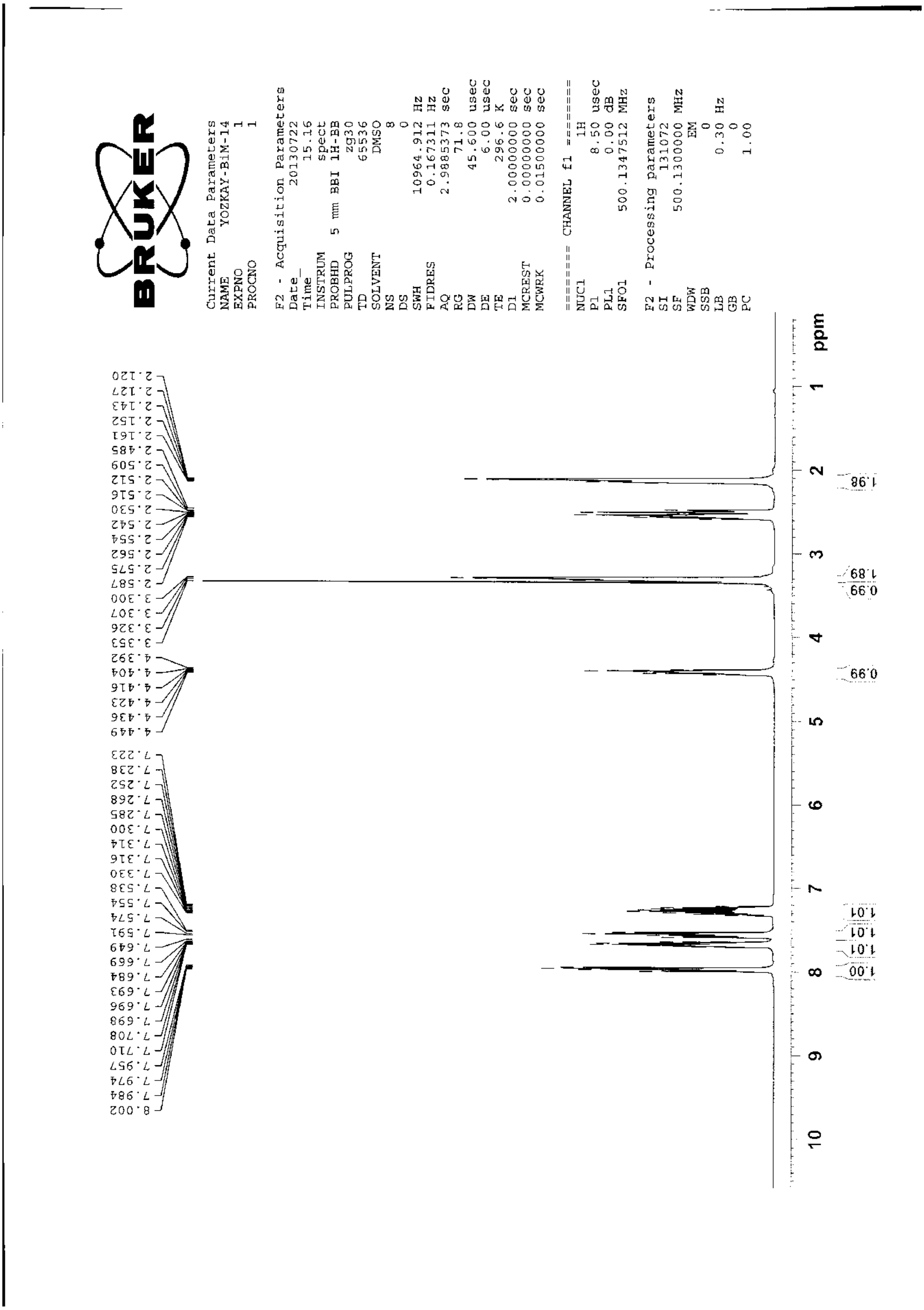

FTIR spectrum of *2-(4-Trifluoromethoxyphenyl)-1-[2-(morpholin-4-yl)ethyl]-1H-benzimidazole* ***(2m)***

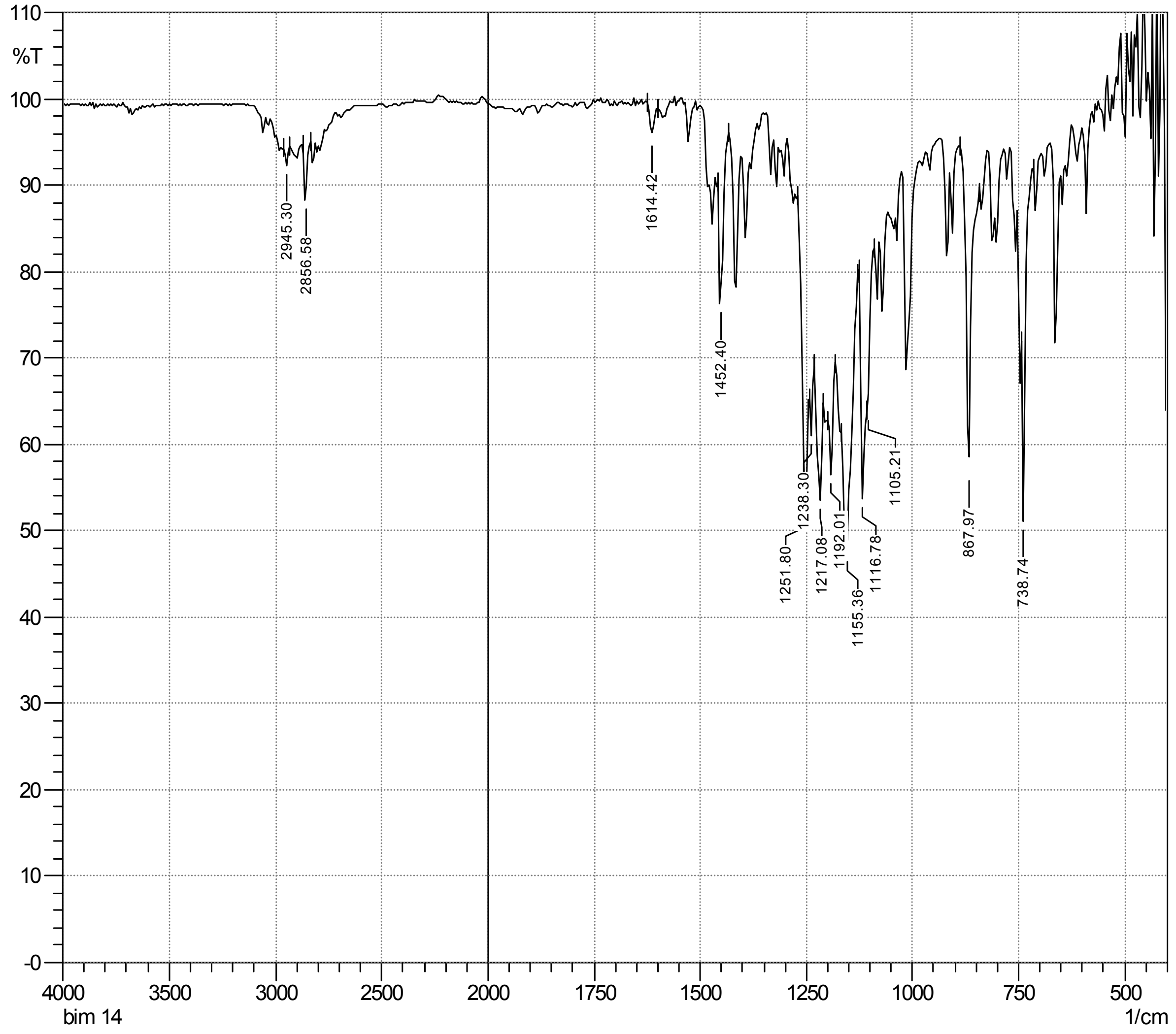

HRMS spectrum of *2-(4-Trifluoromethoxyphenyl)-1-[2-(morpholin-4-yl)ethyl]-1H-benzimidazole (2m)*

Data File: C:\ LabSolutions\ Data\Analiz\Bim serisi\ Bim-14_22.lcd

Elmt	Val.	Min	Max	Elmt	Val.	Min	Max	Elmt	Val.	Min	Max	Use Adduct
H	1	14	30	O	2	2	3	Cl	1	0	0	H
C	4	12	30	F	1	0	3	Br	1	0	0	
N	3	3	4	S	2	0	0					

Error Margin (ppm): 15
HC Ratio: unlimited
Max Isotopes: 3
MSn Iso RI (%): 10.00

DBE Range: -2.0 - 1000.0
Apply N Rule: yes
Isotope RI (%): 1.00
MSn Logic Mode: AND

Electron Ions: both
Use MSn Info: no
Isotope Res: 10000
Max Results: 500

Event#: 1 MS(E+) Ret. Time : 5.440 -> 5.493 Scan# : 817 -> 825

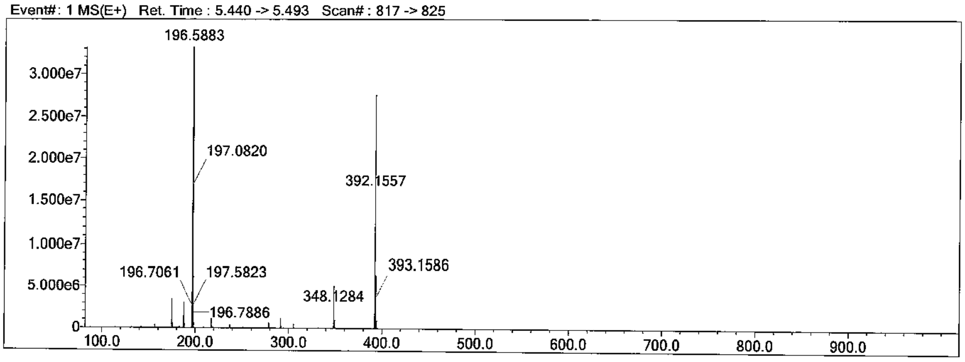

Measured region for 392.1557 m/z

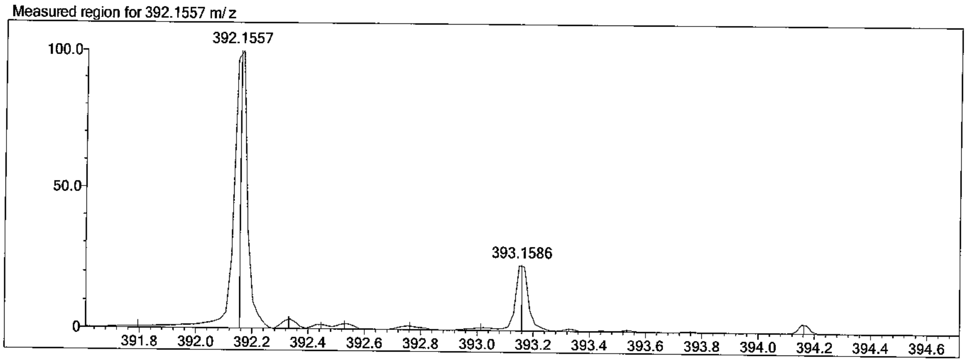

C20 H20 N3 O2 F3 [M+H]+ : Predicted region for 392.1580 m/z

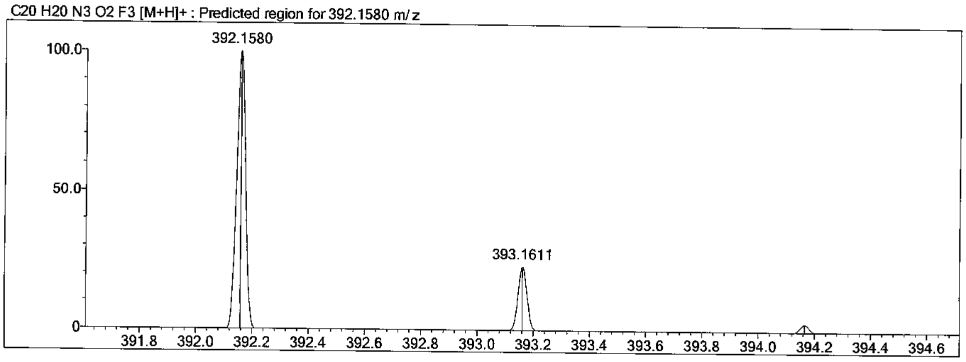

Rank	Score	Formula (M)	Ion	Meas. m/z	Pred. m/z	Df. (mDa)	Df. (ppm)	Iso	DBE
1	81.30	C20 H20 N3 O2 F3	[M+H]+	392.1557	392.1580	-2.3	-5.87	100.00	11.0

13C-NMR spectrum of *2-(4-Methylthiophenyl)-1- [2-(morpholin-4-yl) ethyl]-1H-benzimidazole* (***2n***)

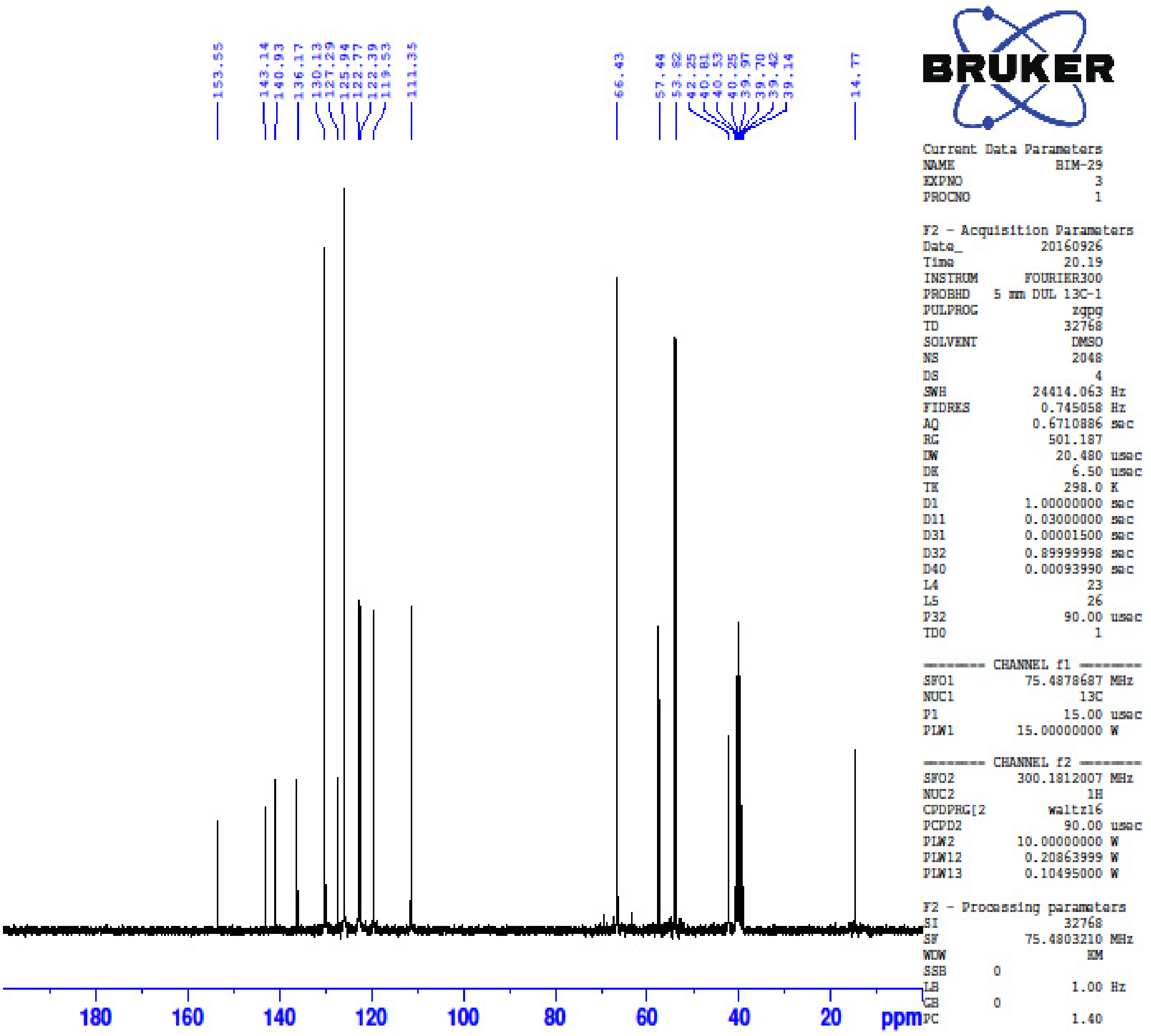

^{1}H-NMR spectrum of *2-(4-Methylthiophenyl)-1- [2-(morpholin-4-yl) ethyl]-1H-benzimidazole* ***(2n)***

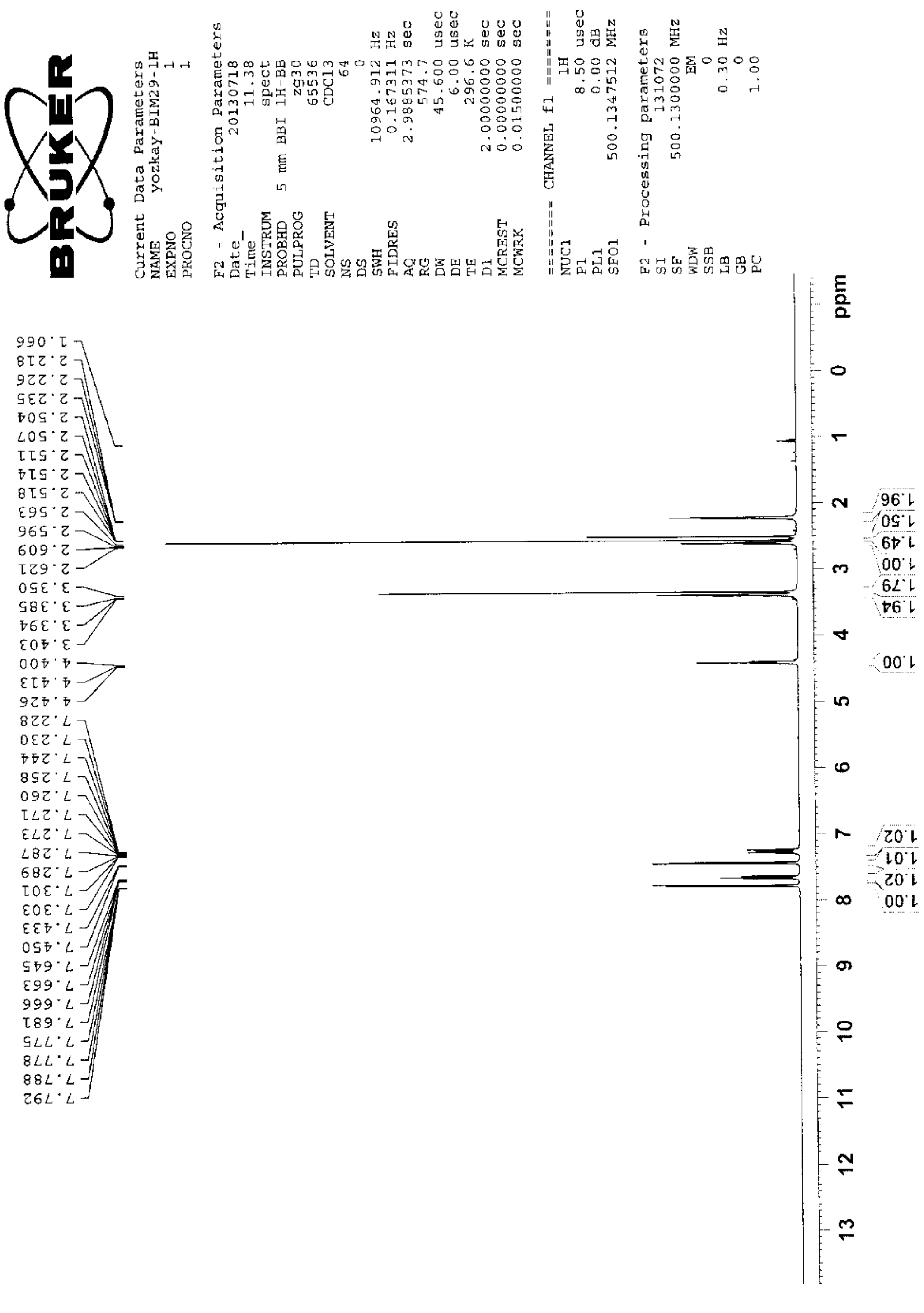

FTIR spectrum of *2-(4-Methylthiophenyl)-1- [2-(morpholin-4-yl) ethyl]-1H-benzimidazole (**2n**)*

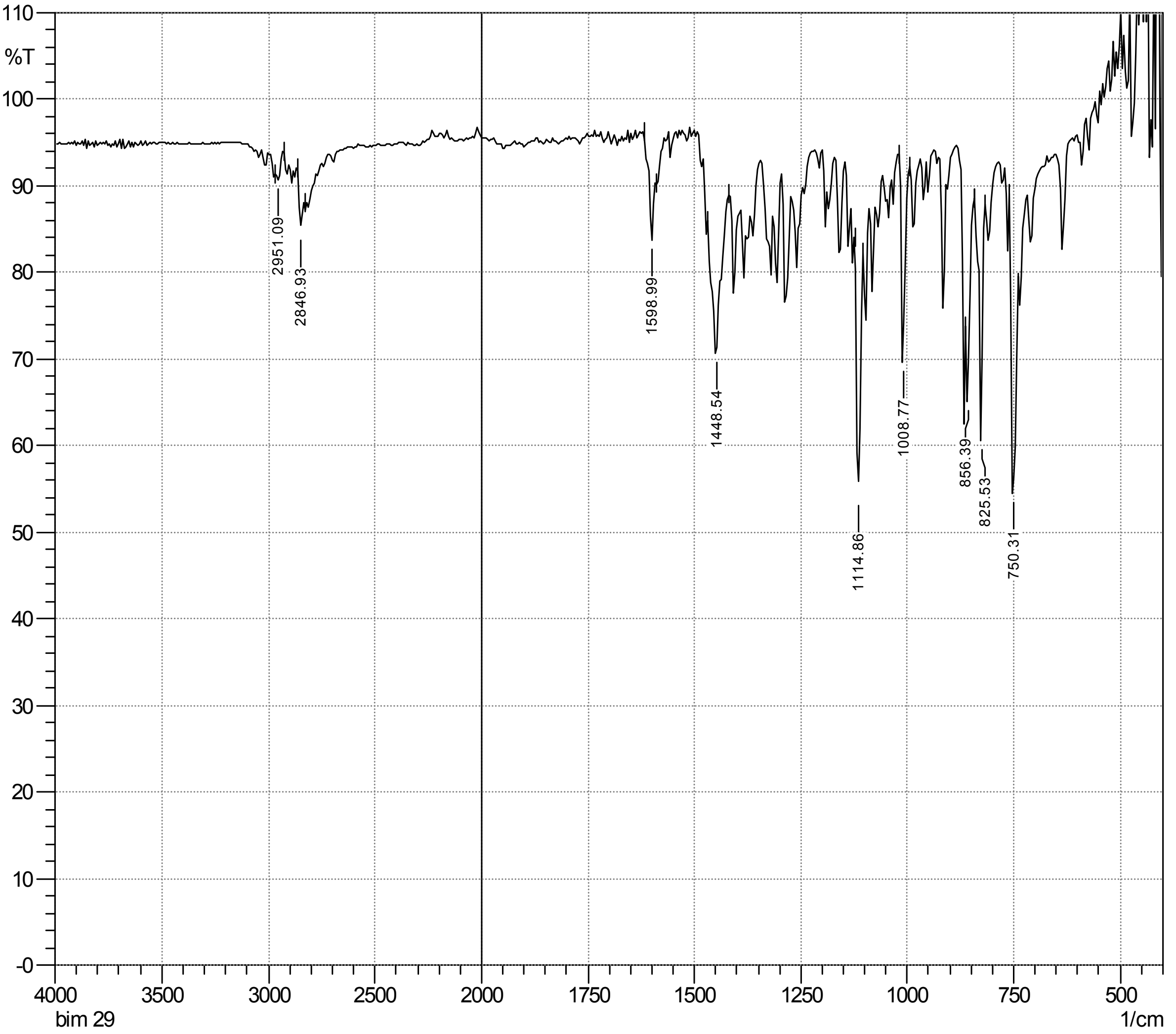

HRMS spectrum of *2-(4-Methylthiophenyl)-1- [2-(morpholin-4-yl) ethyl]-1H-benzimidazole* (**2n**)

Formula Predictor Report - Bim-29_01.lcd

Data File: C:\ LabSolutions\ Data\Analiz\Bim serisi\ Bim-29_01.lcd

Elmt	Val.	Min	Max	Elmt	Val.	Min	Max	Elmt	Val.	Min	Max	Use Adduct
H	1	14	30	O	2	1	3	Cl	1	0	0	H
C	4	12	30	F	1	0	0	Br	1	0	0	
N	3	3	4	S	2	1	1					

Error Margin (ppm): 15
HC Ratio: unlimited
Max Isotopes: 3
MSn Iso RI (%): 10.00

DBE Range: -2.0 - 1000.0
Apply N Rule: yes
Isotope RI (%): 1.00
MSn Logic Mode: AND

Electron Ions: both
Use MSn Info: no
Isotope Res: 10000
Max Results: 500

Event#: 1 MS(E+) Ret. Time : 4.800 -> 4.800 Scan# : 721 -> 721

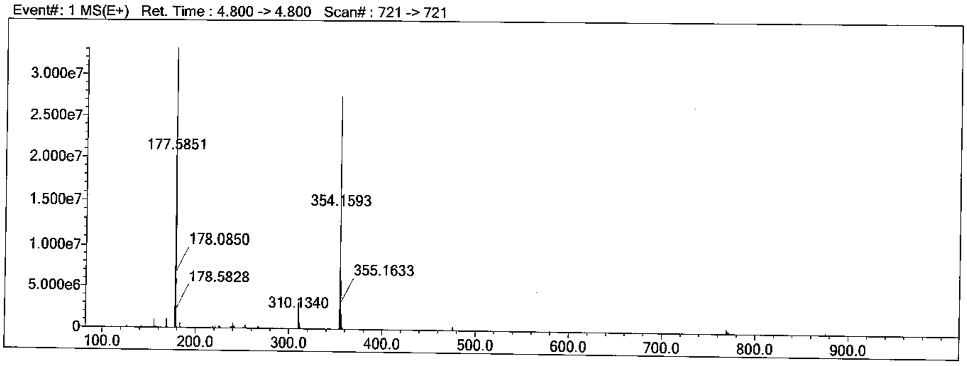

Measured region for 354.1593 m/z

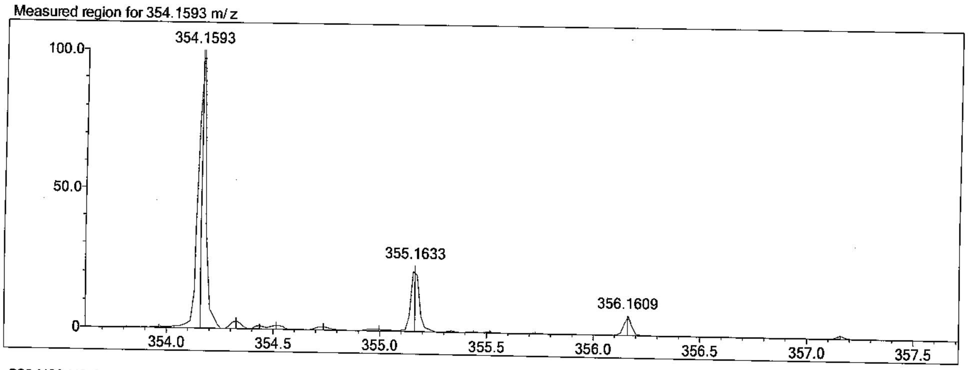

C20 H23 N3 O S [M+H]+ : Predicted region for 354.1635 m/z

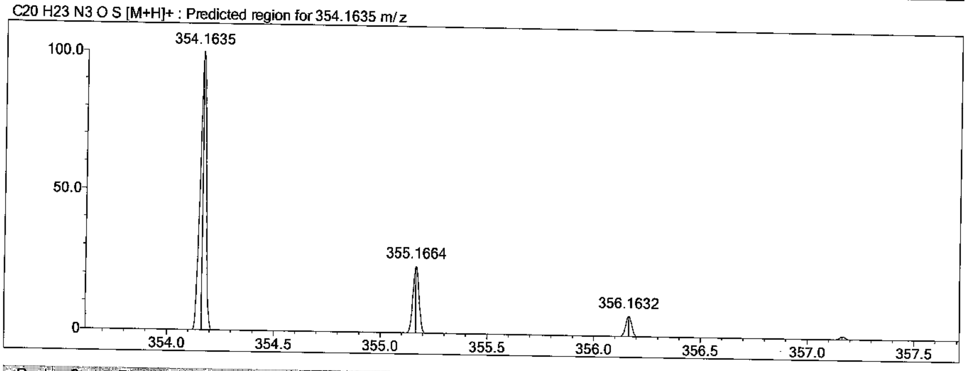

Rank	Score	Formula (M)	Ion	Meas. m/z	Pred. m/z	Df. (mDa)	Df. (ppm)	Iso	DBE
1	29.29	C20 H23 N3 O S	[M+H]+	354.1593	354.1635	-4.2	-11.86	83.58	11.0

^{13}C-NMR spectrum of *2-(1,1'-Biphenyl)-1-[2-(morpholin-4-yl)ethyl]-1H-benzimidazole* (**2o**)

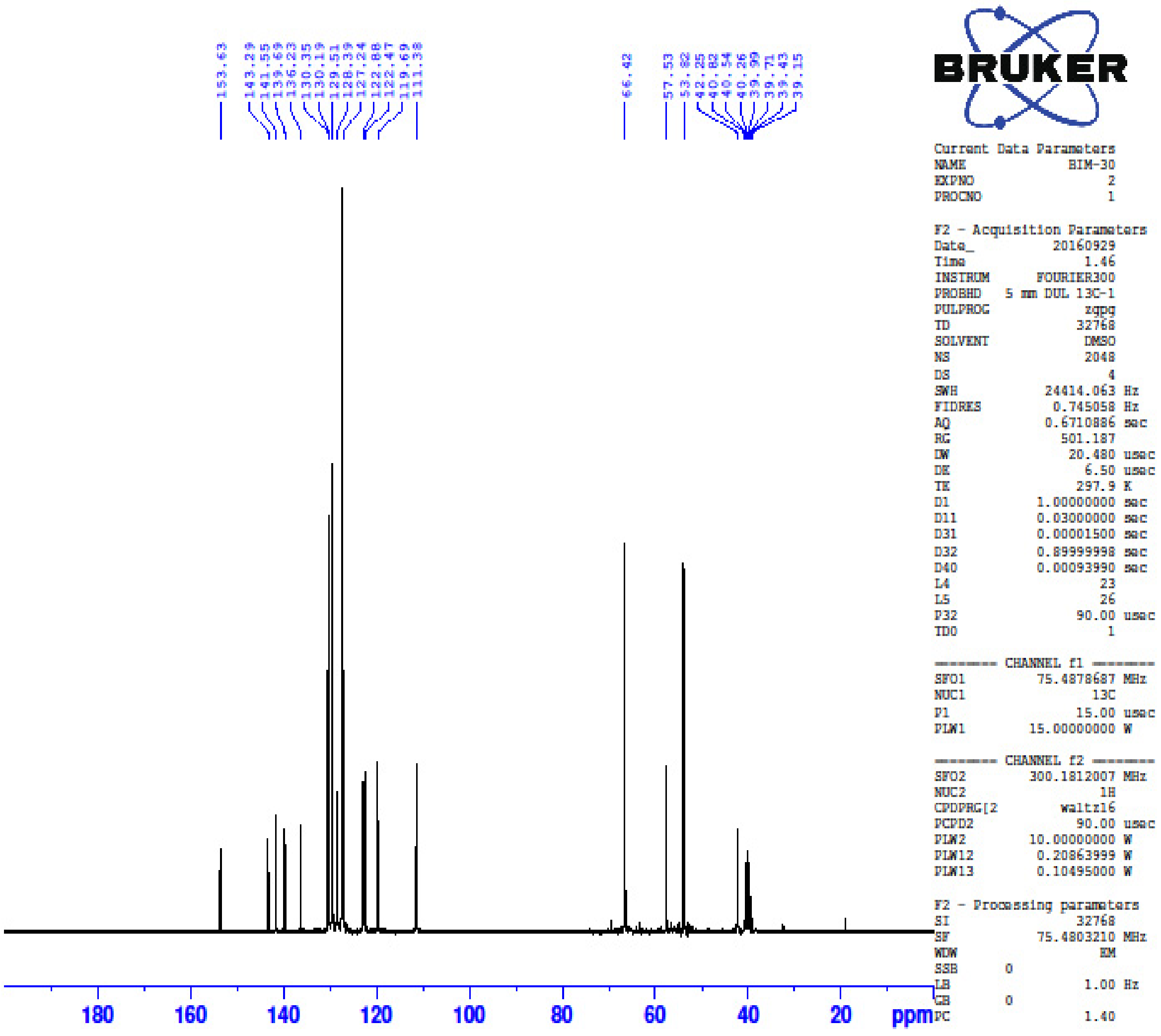

^{1}H-NMR spectrum of *2-(1,1'-Biphenyl)-1-[2-(morpholin-4-yl)ethyl]-1H-benzimidazole (**2o**)*

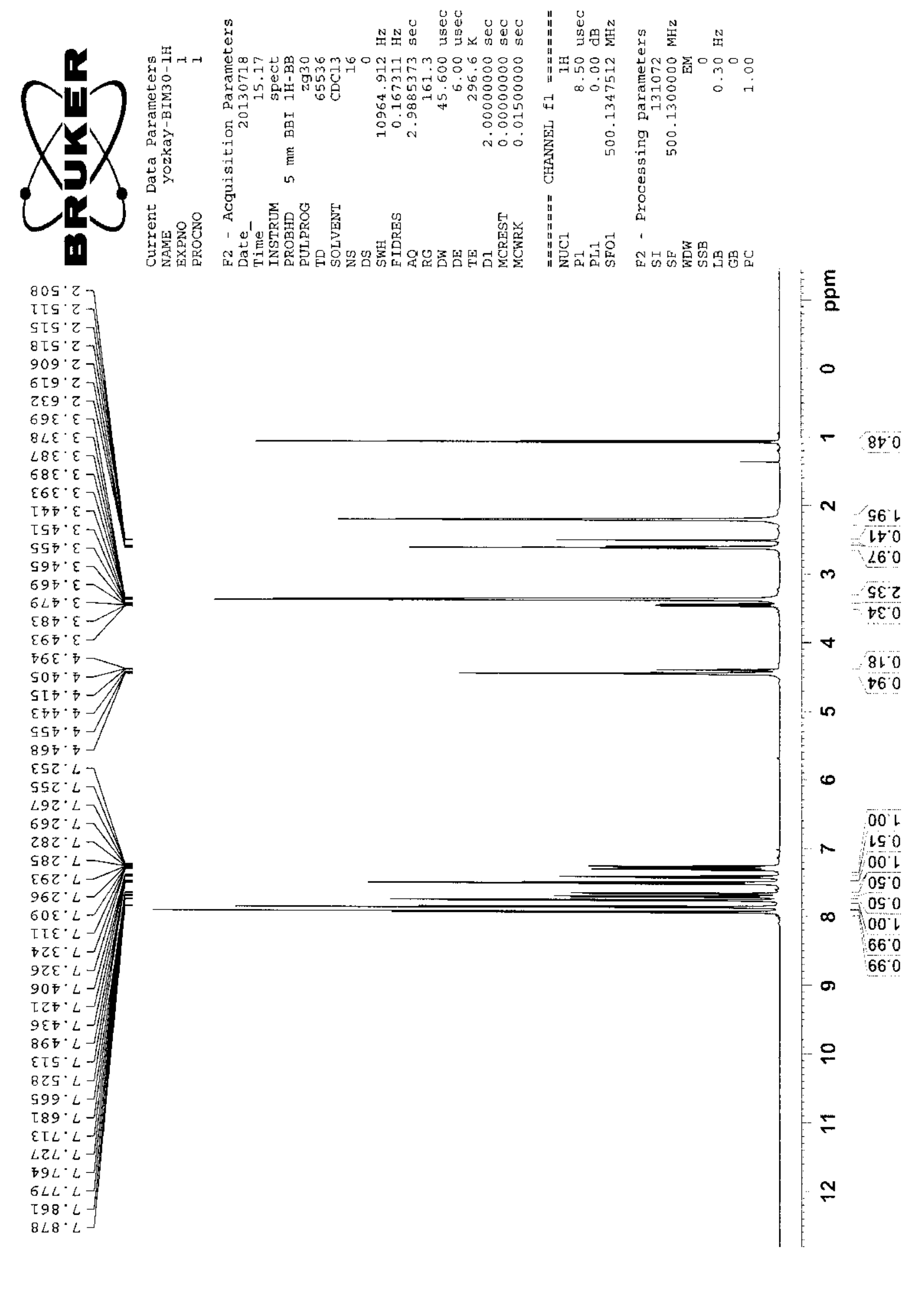

FTIR spectrum of *2-(1,1'-Biphenyl)-1-[2-(morpholin-4-yl)ethyl]-1H-benzimidazole* ***(2o)***

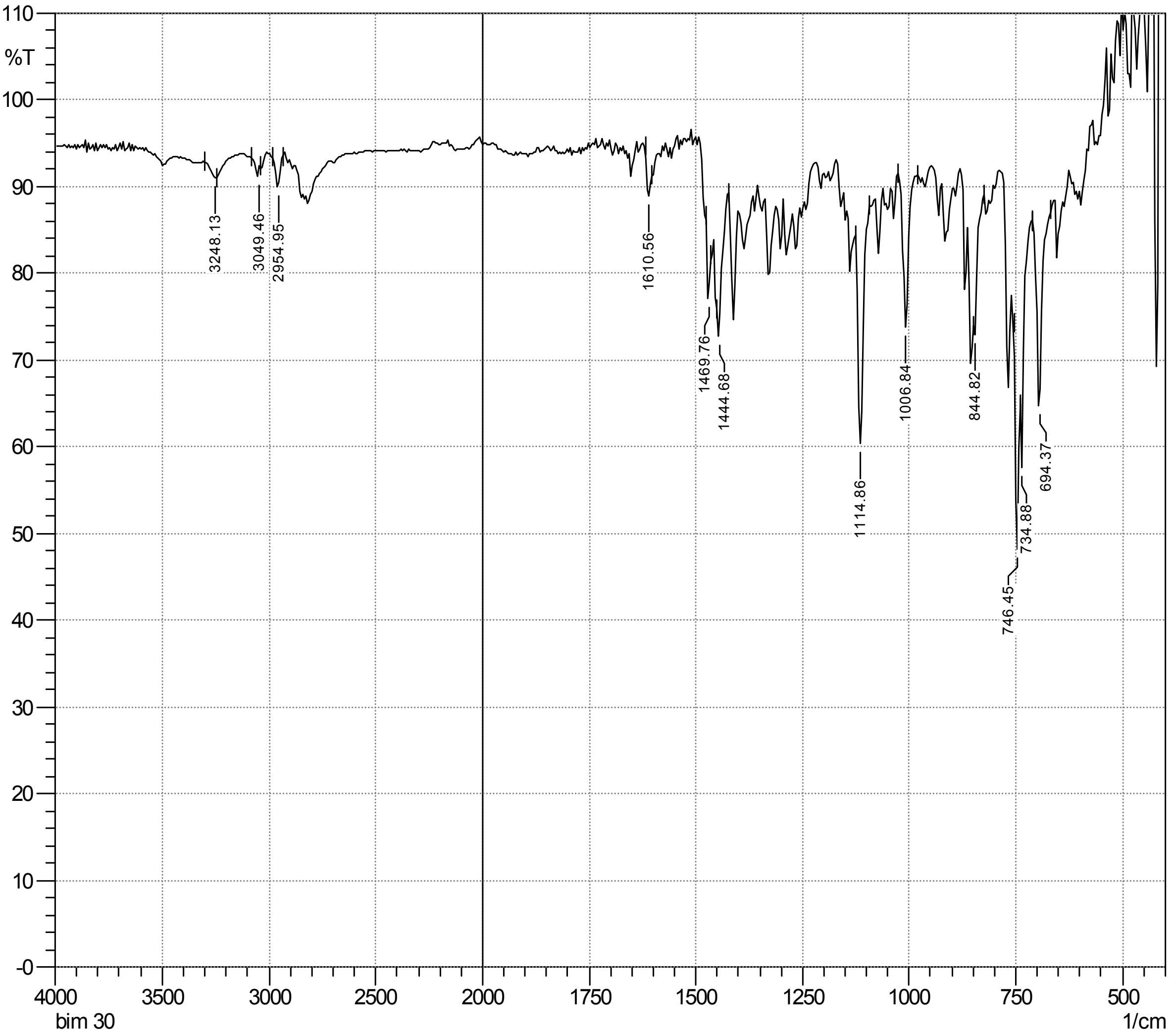

HRMS spectrum of *2-(1,1'-Biphenyl)-1-[2-(morpholin-4-yl)ethyl]-1H-benzimidazole* (**2o**)

Formula Predictor Report - Bim-30_38.lcd

Data File: C:\ LabSolutions\ Data\Analiz\Bim serisi\ Bim-30_38.lcd

Elmt	Val.	Min	Max	Elmt	Val.	Min	Max	Elmt	Val.	Min	Max	Use Adduct
H	1	14	30	O	2	1	3	Cl	1	0	0	H
C	4	12	30	F	1	0	0	Br	1	0	0	
N	3	3	4	S	2	0	0					

Error Margin (ppm): 15
HC Ratio: unlimited
Max Isotopes: 3
MSn Iso RI (%): 10.00

DBE Range: -2.0 - 1000.0
Apply N Rule: yes
Isotope RI (%): 1.00
MSn Logic Mode: AND

Electron Ions: both
Use MSn Info: no
Isotope Res: 10000
Max Results: 500

Event#: 1 MS(E+) Ret. Time : 5.333 -> 5.413 Scan# : 801 -> 813

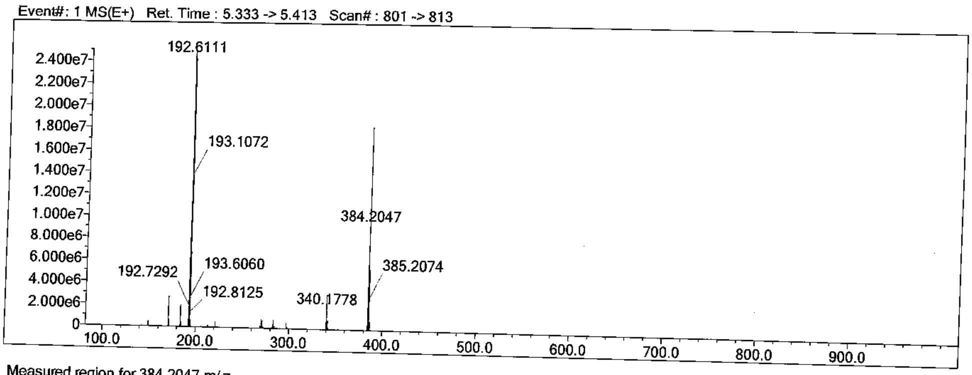

Measured region for 384.2047 m/z

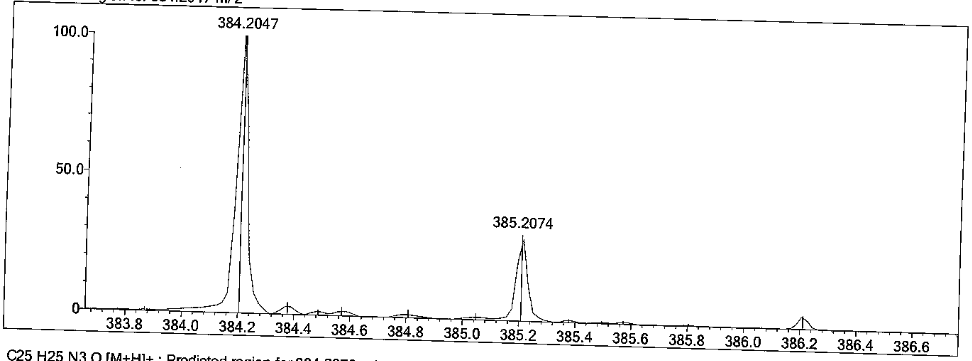

C25 H25 N3 O [M+H]+ : Predicted region for 384.2070 m/z

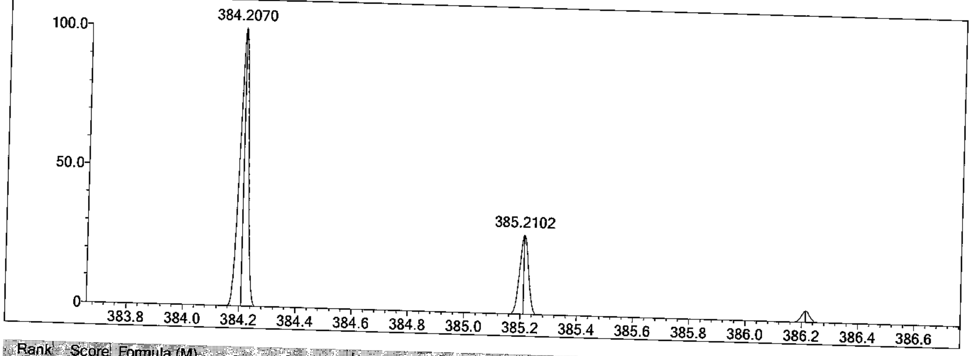

Rank	Score	Formula (M)	Ion	Meas. m/z	Pred. m/z	Df. (mDa)	Df. (ppm)	Iso	DBE
1	51.26	C25 H25 N3 O	[M+H]+	384.2047	384.2070	-2.3	-5.99	64.00	15.0
